# Towards a functional hypothesis relating anti-islet cell autoimmunity to the dietary impact on microbial communities and butyrate production

**DOI:** 10.1186/s40168-016-0163-4

**Published:** 2016-04-26

**Authors:** David Endesfelder, Marion Engel, Austin G. Davis-Richardson, Alexandria N. Ardissone, Peter Achenbach, Sandra Hummel, Christiane Winkler, Mark Atkinson, Desmond Schatz, Eric Triplett, Anette-Gabriele Ziegler, Wolfgang zu Castell

**Affiliations:** Scientific Computing Research Unit, Helmholtz Zentrum München, Munich, Germany; Department of Microbiology and Cell Science, Institute of Food and Agricultural Sciences, University of Florida, Munich, USA; Institute of Diabetes Research, Helmholtz Zentrum München, and Forschergruppe Diabetes, Klinikum rechts der Isar, Technische Universität München, Munich, Germany; Department of Pediatrics, University of Florida, Gainesville, FL USA; Department of Mathematics, Technische Universität München, Munich, Germany

**Keywords:** Gut microbiome, Type 1 diabetes, Islet immunity, Mucin degradation, Interaction networks, Butyrate

## Abstract

**Background:**

The development of anti-islet cell autoimmunity precedes clinical type 1 diabetes and occurs very early in life. During this early period, dietary factors strongly impact on the composition of the gut microbiome. At the same time, the gut microbiome plays a central role in the development of the infant immune system. A functional model of the association between diet, microbial communities, and the development of anti-islet cell autoimmunity can provide important new insights regarding the role of the gut microbiome in the pathogenesis of type 1 diabetes.

**Results:**

A novel approach was developed to enable the analysis of the microbiome on an aggregation level between a single microbial taxon and classical ecological measures analyzing the whole microbial population. Microbial co-occurrence networks were estimated at age 6 months to identify candidates for functional microbial communities prior to islet autoantibody development. Stratification of children based on these communities revealed functional associations between diet, gut microbiome, and islet autoantibody development. Two communities were strongly associated with breast-feeding and solid food introduction, respectively. The third community revealed a subgroup of children that was dominated by *Bacteroides* abundances compared to two subgroups with low *Bacteroides* and increased *Akkermansia* abundances. The *Bacteroides*-dominated subgroup was characterized by early introduction of non-milk diet, increased risk for early autoantibody development, and by lower abundances of genes for the production of butyrate via co-fermentation of acetate. By combining our results with information from the literature, we provide a refined functional hypothesis for a protective role of butyrate in the pathogenesis of type 1 diabetes.

**Conclusions:**

Based on functional traits of microbial communities estimated from co-occurrence networks, we provide evidence that alterations in the composition of mucin degrading bacteria associate with early development of anti-islet cell autoimmunity. We hypothesize that lower levels of *Bacteroides* in favor of increased levels of *Akkermansia* lead to a competitive advantage of acetogens compared to sulfate reducing bacteria, resulting in increased butyrate production via co-fermentation of acetate. This hypothesis suggests that butyrate has a protective effect on the development of anti-islet cell autoantibodies.

**Electronic supplementary material:**

The online version of this article (doi:10.1186/s40168-016-0163-4) contains supplementary material, which is available to authorized users.

## Background

The human holobiont is defined as the human host together with all its associated microorganisms [1] colonizing various regions of the human body. By far, the largest community of microorganisms resides in the large intestine where an estimated number of 10^12^ bacteria per gram can be found in stool samples [2]. The microbial community of the gastrointestinal tract serves several functions including fermentation of unabsorbed nutrients, partnering the human immune system, or providing barrier against pathogenic attacks [2, 3]. Constituting an important ecosystem within the human body, gut microorganisms are increasingly considered to play a crucial role in the development of autoimmune diseases [4, 5] such as type 1 diabetes (T1D).

T1D is an autoimmune disease that results in destruction of insulin producing cells in the islets of Langerhans which is preceded by the development of islet autoantibodies. Thus, seroconversion to islet autoantibodies is considered to be an important checkpoint in pathogenesis of T1D [6]. Interestingly, in individuals with a high risk background for T1D, incidences of seroconversion to autoantibody positivity peak within the period of 9 months to 2 years of age and a second, less prominent peak has been observed at approximately 8 years of age [6]. Beyond this, the first months of life are a distinguished period for the development of the human immune system. Thus, major switches in the transformation of mucosal barrier function in the gut reside within this time period [7]. While it is generally agreed on that genetic background constitutes approximately 60 % of T1D risk [8], the remaining propensity to disease has been attributed to several environmental factors including diet, early infections, or mode of delivery [9, 10]. Among these, early dietary factors along with early programming of the immune system can be associated with microorganisms colonizing the body. Being the organ where nutritional components have to pass the epithelial barrier, as well as a site of high metabolic activity, the gut ecosystem has the highest density of immune cells within the human body [11]. This said, reports on T1D risk being associated with gut bacteria do not yet provide a clear picture [5, 12, 13].

Several studies have shown an association between altered gut microbial communities and autoimmunity [14–19]. However, although several researchers analyzed the gut microbiome after autoantibody development [14, 16, 19] or T1D onset [20, 21], few studies focus on the period prior to autoantibody development [15, 17, 18]. Among the first, Giongo et al. [17] reported a shift in the ratio of Bacteroidetes and Firmicutes in a group of eight Finnish children. Recently, higher abundances of *Bacteroides dorei* prior to autoantibody seroconversion have been associated with increased risk of islet autoantibodies [15]. This finding aligns with earlier reports on increases of abundances of *Bacteroides* spp. subsequent to islet autoantibodies and/or T1D disease onset [14, 20, 21]. Brown et al. [16] presented a metagenomics analysis showing lower portions of butyrate-producing and mucin-degrading bacteria in autoantibody positive children. In the German BABYDIET study, differences on the level of the single bacterium could not be seen, while the overall microbial community structure was compromised in individuals who later developed islet autoantibodies [18].

Early dietary effects, such as breast-feeding, have been reported to influence T1D development. However, contradictory results have been published. In general, breast-feeding seems to exhibit a protective role for T1D [22] in retrospective analyses, while prospective cohorts [10, 23] have not been able to confirm this observation. Rather, there is increasing evidence that increased risk for T1D is associated with early introduction of complex diet, in particular gluten and cereals [10, 24, 25], or fruits and berries [23, 26].

To our knowledge, the German BABYDIET cohort [18, 27] is currently the largest prospective cohort providing detailed dietary protocols as well as longitudinal microbial 16S rRNA amplicon sequencing data and information about the development of islet autoantibodies. Thus, this cohort provides a unique opportunity to analyze the association between infant diet and gut microbial communities with respect to the development of islet autoantibodies. Being a unique source, we reanalyzed the publicly available 16S rRNA amplicon data with regard to the impact of the gut microbiome on the development of islet autoimmunity prior to seroconversion. Our aim hereby was to derive a functional hypothesis which may provide guidelines for future design of cohorts.

When addressing what key functionalities stabilize the gut microbial community and balance its interaction with the host, neither the properties on the level of the community as a whole nor the taxonomic identities provide sufficient information. Thus, our objective was to provide a level for functional analysis that reaches beyond a single microbial taxon and at the same time being finer than classical measures on the community level such as microbial diversity. Through applying community analysis on co-occurrence networks of bacteria, we show that three communities can be identified representing functional groups of microbial genera. In particular, this intermediate level of observation allows dissecting the “dietary age” of the children. The latter turned out to have much stronger influence on the gut microbial community than the actual biological age. Stratification of children on the level of microbial communities unravels alterations in the ensemble of mucin degrading bacteria in the intestinal flora of children who later developed islet autoantibodies. Specifically, lower abundances of *Akkermansia* in favor of *Bacteroides,* together with a functional shift in butyrate metabolism, associate with early introduction of non-milk diet, in particular meat, as well as with higher risk of seroconversion early in life. Summarizing, our analysis suggests that instead of the biological age, the dietary age should be considered for the analysis of the gut microbiome with respect to autoantibody development. Due to substantial variations regarding the biological age at seroconversion, accounting for diet is of particular importance when dealing with associations between microbial factors and seroconversion.

## Methods

### BABYDIET study and 16S rRNA gene sequencing

In total 298 stool samples from 44 children participating in the BABYDIET study were used for microbiome analysis. These included 147 samples from 22 children who developed persistent anti-islet cell autoantibodies at a median age of 1.54 years (IQR 0.90 years and maximum 2.45 years) and 151 samples from 22 children who remained anti-islet cell autoantibody negative. These latter subjects were also matched for date of birth. On average 6.8 stool samples per child were taken from age 0.24 to 3.2 years. To analyze microbial communities before the development of the first autoantibody, we used samples from children that had at least one probe between age 3 and 9 months. The probe closest to 6 months of age was used if several probes per child were available. This resulted in a total of 40 children, including 19 autoantibody positive and 21 autoantibody negative children. None of these children had already developed their first positive autoantibody. A detailed description of the sample collection, the questionnaire on dietary intake, and the BABYDIET cohort can be found in [18] and [27]. In brief, data on breast-feeding, the duration of breast-feeding, and the introduction of solid food (gluten-free and gluten-containing cereals, vegetables, fruits, potato, and meat) were taken from daily food records completed by the child’s parents. Written informed consent was obtained from the parents. The study was approved by the ethics committee of the Ludwig-Maximilian-University, Munich, Germany (Ethikkommission der Medizinischen Fakultät der Ludwig-Maximilians Universität No. 329/00). Stool sample collection and 16S rRNA gene sequencing was performed as described in [18]. In brief, PCR was performed at an initial denaturation temperature of 94 °C for 3 min, followed by 20 cycles of 94 °C for 45 s, 50 °C for 30 s, and 65 °C for 90 s. A final elongation step at 65 °C was run for 10 min. PCR products were purified using the Qiagen™ PCR purification kit following the manufacturer’s protocol [18]. The V4 region of the 16S rRNA genes was used, and bacterial 16S genes were amplified using the primers 515F and 806R. Deviating from the procedure described in [18], sequences were aligned to the Greengenes 13.8 database [28] at 97 % identity using the USEARCH program version 6.022 and low quality reads were trimmed as described in [15]. The Illumina 16S sequences are available from NCBI’s short read archive (accession number SRP063271). To account for the influence of sequencing artifacts, operational taxonomic units (OTUs) that were not present with ≥50 reads in ≥10 samples were excluded from all further analyses.

### Statistical analysis

**Fig. 1:**
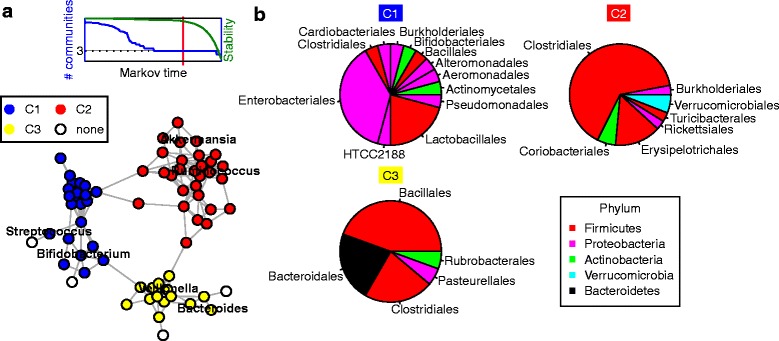
Description of co-occurrence network-based microbial communities. **a** Co-occurrence network of microbial genera at age 6 months. Each node represents one microbial genus and colors (*blue*, *red*, *yellow*) indicate microbial communities. Genera that had one or no connection to other genera were not assigned to communities (*white nodes*) (**b**) Taxonomic composition of the communities C1, C2, and C3. The segments of the pies show the percentage of bacterial genera in a community that can be summarized on order level. Similarly, the colors show the percentage of bacterial genera in a community that can be summarized on phylum level

The aim of this study was to develop a method for the identification of stable gut microbial communities from interaction networks prior to the development of anti-islet autoantibodies, and thus, providing a novel opportunity to analyze gut microbial communities on a level beyond the single bacterium and the microbial community as a whole. In a first step, we estimated microbial communities based on co-occurrence networks. Next, microbial communities were validated by analyzing their association with dietary factors and last but not least microbial communities were tested for associations with the development of anti-islet autoantibodies. In total, 1048 OTUs were detected, and classified OTUs (*N* = 563) were aggregated on genus level using the Greengenes 13.8 database [28]. To avoid bias due to sequencing artifacts, genera with less than 0.01 % abundance within the total number of reads were neglected for community analysis. The CCREPE [29] method was used to estimate Spearman’s rank correlations (*ρ*) with *P* values corrected for compositional data. An edge was set between two bacterial genera if the *P* < 0.05 and *ρ* > 0.4. For further network analysis, the largest connected component of the network was selected. Community analysis was applied to the network using the Markov Dynamics clustering algorithm by [30] implemented in MATLAB^®^. This algorithm allows identification of clique-like communities within a continuous range of a parameter (i.e., Markov time), capturing dynamic characteristics of processes on the network. To determine the number of communities, we chose a number larger than two showing longest stability with respect to Markov time (see Fig. 1a). To analyze the association of the identified gut microbial communities with diet and autoantibody development, children were stratified based on genera in each of the communities separately. Based on UniFrac distance [31], clustering of children was performed with the Partitioning Around Medoids method [32], and the number of clusters was determined by the Calinski-Harabasz method implemented in the R package *fpc* [33]. The number of food ingredients (potato, meat, vegetables, fruit, and formula milk) was used to determine a score for the complexity of the diet for each individual. *P* values for the differences in breast-feeding frequencies between subgroups of children were obtained by comparing the number of children who were still breast-fed vs. children who were no longer breast-fed by two-sided Fisher’s exact tests. Similarly, *P* values for food complexity were obtained by comparing individuals who were already fed >3 vs. individuals who were fed ≤3 different food ingredients by two-sided Fisher’s exact tests. Finally, the PICRUSt method [34] was used to infer in silico metagenomes based on the KEGG database [35, 36] from the 16S OTU table. To compare subgroups of children regarding their genetic potential to produce butyrate via the phosphotransbutyrylase and butyrate kinase pathway (K00634 and K00929) or the butyryl CoA:acetate CoA transferase pathway (K01034 and K01035), we compared the relative abundances of the KEGG genes (KOs) required for the last steps in these pathways. All statistical analyses were performed with R version 3.0.2 and MATLAB^®^ 2012b. The full documentation of all statistical analyses can be found in Additional file 1, and the required OTU and KO abundance tables can be found in Additional files 2 and 3. If not mentioned otherwise, two-sided *P* values were used throughout the manuscript.

## Results

### Co-occurrence network-based bacterial communities at age 6 months

The analysis of microbial co-occurrence patterns can provide valuable insights into factors driving the assembly of functional microbial communities. Co-occurrence networks were analyzed at 6 months of age as the focus of our approach was on the functional role of the gut microbiome with regard to the development of the infant immune system prior to autoantibody seroconversion. Furthermore, at this age, diet has a substantial impact on the microbial community as changes from breast-feeding to more complex food ingredients often occur at approximately 6 months of age. Using an a priori criterion for stability based on Markov time (see the “Methods” section), three clique-like stable communities (C1, C2, and C3) could be clearly identified in the association network of genera determined in samples of 6 months of age (Fig. 1; Additional file 4: Figure S1). Community C1 consisted to a significantly larger part of the taxonomic orders Enterobacteriales (38 %, *P* < 0.00001) and Lactobacillales (21 %, *P* = 0.002, Fig. 1b) when compared to C2 and C3 and was the only community that included genera from the order Bifidobacteriales. Community C2 constituted mainly of Clostridiales (65 %, *P* < 0.00001) and Erysipelotrichales (15 %, *P* = 0.02, Fig. 1b), both from the phylum Firmicutes. Thus, this group contained several specialists that are characteristic for an adult-like community, such as *Ruminococcus*, *Blautia*, or *Akkermansia* (Fig. 1b). Community C3 included a large proportion of Bacteroidetes (22 %, *P* = 0.002, Fig. 1b). Although Firmicutes were also providing the majority within this community (67 %), there were much less Clostridiales (22 %) present within this group compared to a significant portion of *Bacillales* (44 %, *P* = 0.00002). Also *Veillonella* and some other Veillonellaceae fell within the third community. Next, we analyzed the temporal development of abundances of genera in the communities using the extended longitudinal data set (see the “Methods” section) gained from samples taken over 2 years. While the majority among dominant bacteria in communities C1 and C2 showed decreasing (C1) or increasing (C2) abundances, respectively, community C3 consisted of both, bacteria expressing increasing as well as decreasing abundances over time (Additional file 4: Figure S2).

### Microbial communities are associated with dietary factors

**Fig. 2:**
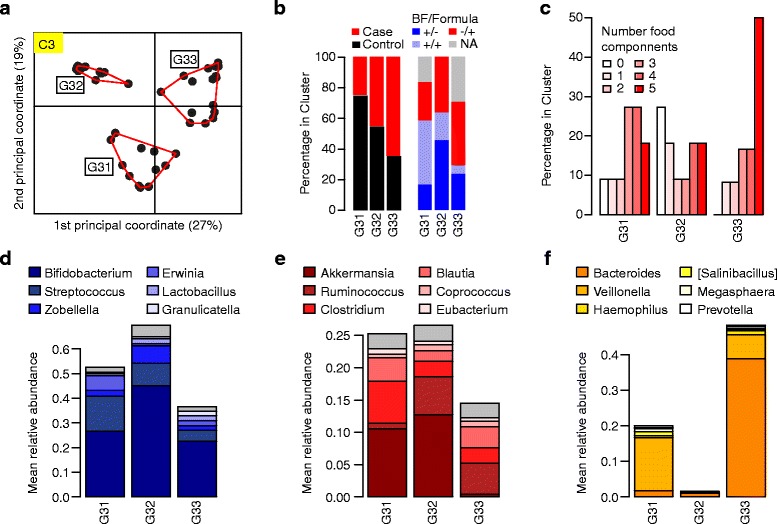
Stratification of children based on genera in C3. **a** The PCoA plot shows three clusters (G31, G32, and G33) of children identified by PAM clustering of UniFrac distances from abundances of genera in C3. **b** Percentages of autoantibody positive and autoantibody negative, and breast-fed vs. formula-fed infants in each subgroup (*+/−*: breast feeding without formula feeding; *+/+*: breast feeding and formula feeding; *−/+*: formula feeding without breast feeding; *NA* no data on breast-feeding available). **c** Percentage of the number of solid food components (vegetables + potatoes + fruits + meat + formula) in each subgroup. **d** Mean relative abundance of the six dominant genera in community C1. **e** Mean relative abundance of the six dominant genera in community C2. **f** Mean relative abundance of the six dominant genera in community C3. Being mainly found in salt lakes [69] *Salinibacillus* might not be expected in the gut habitat. We therefore performed a refined clustering for sequences of OTUs annotated to *Salinibacillus* using ARB [70]. The resulting clustering of these sequences showed closer similarity to *Bacillus* (data not shown), suggesting that *Salinibacillus* was wrongly annotated

The associations of the gut microbial composition with breast-feeding and the introduction of solid food have been extensively described in the literature [37]. We therefore analyzed the association of diet with the three microbial communities as a validation of our approach to identify functional groups of bacteria on a community level. The taxonomic composition of communities C1 and C2 already suggested that diet might be a major factor being reflected through bacteria in these communities. In contrast, no obvious dietary pattern was seen for community C3, suggesting that this group characterizes additional factors influencing community composition. Stratification of children based on abundances of genera in each community (Additional file 5: Table S1) confirmed that diet associated with abundances in communities C1 and C2. While community C1 showed increased abundances in breast-fed individuals (G11 and G12 in Additional file 4: Figure S3B, *P* = 0.012) and decreased abundances in children who were fed a more complex diet (G13 and G14 in Additional file 4: Figure S3C, *P* = 0.00006), community C2 revealed the opposite pattern with decreased abundances in breast-fed children (G21 and G23 in Additional file 4: Figure S4B, P = 0.011) and increased abundances in children that were given a more complex diet (G22 in Additional file 4: Figure S4C, *P* = 0.010). While children in the *Bifidobacterium*-dominated subgroup G11 were mostly breast-fed without being fed formula milk in addition (Additional file 4: Figure S3B and S3D), subgroup G12 had almost equal abundances in *Bifidobacterium*, *Streptococcus*, and some Proteobacteria. At the same time, this subgroup included more children that were fed formula in addition to breast milk (Additional file 4: Figure S3B and S3D). In contrast, children showing higher abundances of *Akkermansia*, *Ruminococcus*, *Clostridium*, and *Blautia* (G13 in Additional file 4: Figure S3C, S3E and G22 in Additional file 4: Figure S4C and S4E) reflect the fact that the complexity of the food taken by those individuals was clearly higher. Although stratification of children based on abundances in community C3 showed a tendency for increased food complexity in a subgroup dominated by *Bacteroides* (G33 in Fig. 2), no significant associations of the subgroups could be observed with breast-feeding or food complexity (Fig. 2b, c).

### Association of a *Bacteroides*-dominated community with anti-islet autoantibody development

**Fig. 3:**
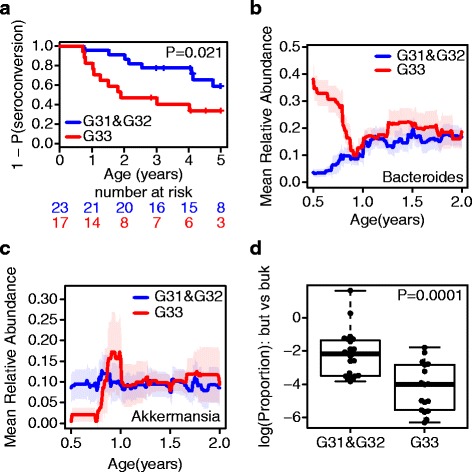
Probability for autoantibody development, temporal development, and butyrate production in community C3. **a** Kaplan-Meier plot comparing the probability for early autoantibody development in subgroups G33 (*red*) vs. G31 and G32 (*blue*). **b** *Bacteroides* abundances vs. age in subgroups G33 (*red*) vs. G31 and G32 (*blue*). **c** *Akkermanisa* abundances vs. age in subgroups G33 (*red*) vs G31 and G32 (*blue*). **d** Comparison of the ratio but-genes/buk-genes in subgroup G33 vs. subgroups G31 and G32

After successfully validating our approach for microbial community detection in terms of their functional association with infant diet, microbial communities at 6 months of age were analyzed for their association with anti-islet autoantibody development. Stratification of children based on communities C1 and C2 did not reveal associations with autoantibody development (Additional file 4: Figure S3B and S4B). Clustering of children with respect to the abundances in community C3 resulted in three clusters (Fig. 2a, Additional file 5: Table S1). While microbial ensembles derived from children in subgroup G33 were dominated by the genus *Bacteroides* and showed almost no abundances in *Akkermansia*, samples from subgroups G31 and G32 had low *Bacteroides* and increased *Akkermansia* abundances (Fig. 2e, f). In line with reported associations of increased *Bacteroides* abundances in children that developed anti-islet autoimmunity or T1D, subgroup G31 comprised significantly more autoantibody negative children, compared to subgroup G33 (Fig. 2b, *P* = 0.041, one-sided Fisher’s exact test). Strikingly, compared to both, G31 and G32, subgroup G33 showed a significantly increased risk of early autoantibody development (Fig. 3a, *P* = 0.021, HR = 2.8). The increased abundances of *Bacteroides* in G33 observed at age 6 months could not be observed at later time points (Fig. 3b). Similarly, *Akkermansia* abundances between the subgroups became more similar at later time points (Fig. 3c). Interestingly, *Bacteroides* showed significantly increased abundances in children that already had meat in their diet (*P* = 0.007, data not shown).

To identify indications of possible functional differences of genera belonging to community C3, we compared functional traits predicted via imputed metagenome data. We observed differences in the production of butyrate via the classical buk-pathway (indicated by the presence of phosphotransbutyrylase and butyrate kinase genes; K00634 and K00929) and the potential to produce butyrate by co-fermentation of acetate (indicated by the presence of butyryl CoA:acetate CoA transferase genes; K01034 and K01035), further on referred to as but-pathway. One can note that the route via co-fermentation yields higher butyrate outcome compared to the buk-pathway [38]. Interestingly, the ratio of but- vs. buk-genes was significantly lower in subgroup G33 (Fig. 3d, *P* = 0.00015) compared to the other two communities. As a whole, clustering based on community C3 revealed a significant association of early autoantibody development in a subgroup with high *Bacteroides* and low *Akkermansia* abundances.

## Discussion

The analysis of co-occurrence patterns clearly allows disentangling dietary effects on gut community composition. Children showing higher abundances in community C1 are still in a state of prevailing milk diet. We can identify two dietary sub-patterns through analyzing this community. There is a *Bifidobacterium*-dominated pattern (Additional file 4: Figure S3D, subgroup G11) that associates with higher percentages of breast-fed individuals. Moving towards an intermediate state of mixed diet including breast-milk and formula is reflected in a microbial composition which shows a more balanced community composition with lactic acid bacteria and Proteobacteria (Additional file 4: Figure S3D, subgroup G12). Likewise, community C2 comprises Clostridiaceae, Ruminococcaceae, and Lachnospiraceae, reflecting the ability of the microbial consortium to degrade an increasing variety of dietary sources, such as complex polysaccharides. This is commonly accompanied with rising abundances of genera from the phylum Firmicutes, primarily of the order Clostridiales [39]. Thus, abundances of genera belonging to community C2 indicate an advanced dietary age of the host (Additional file 4: Figure S4E and C, subgroup G22). Overall, communities C1 and C2 validate the assumption that the chosen community approach is a suitable way to identify functional groups of bacteria, reflecting adaptation of the microbial ensembles to dynamic changes in their host-defined habitat.

**Fig. 4:**
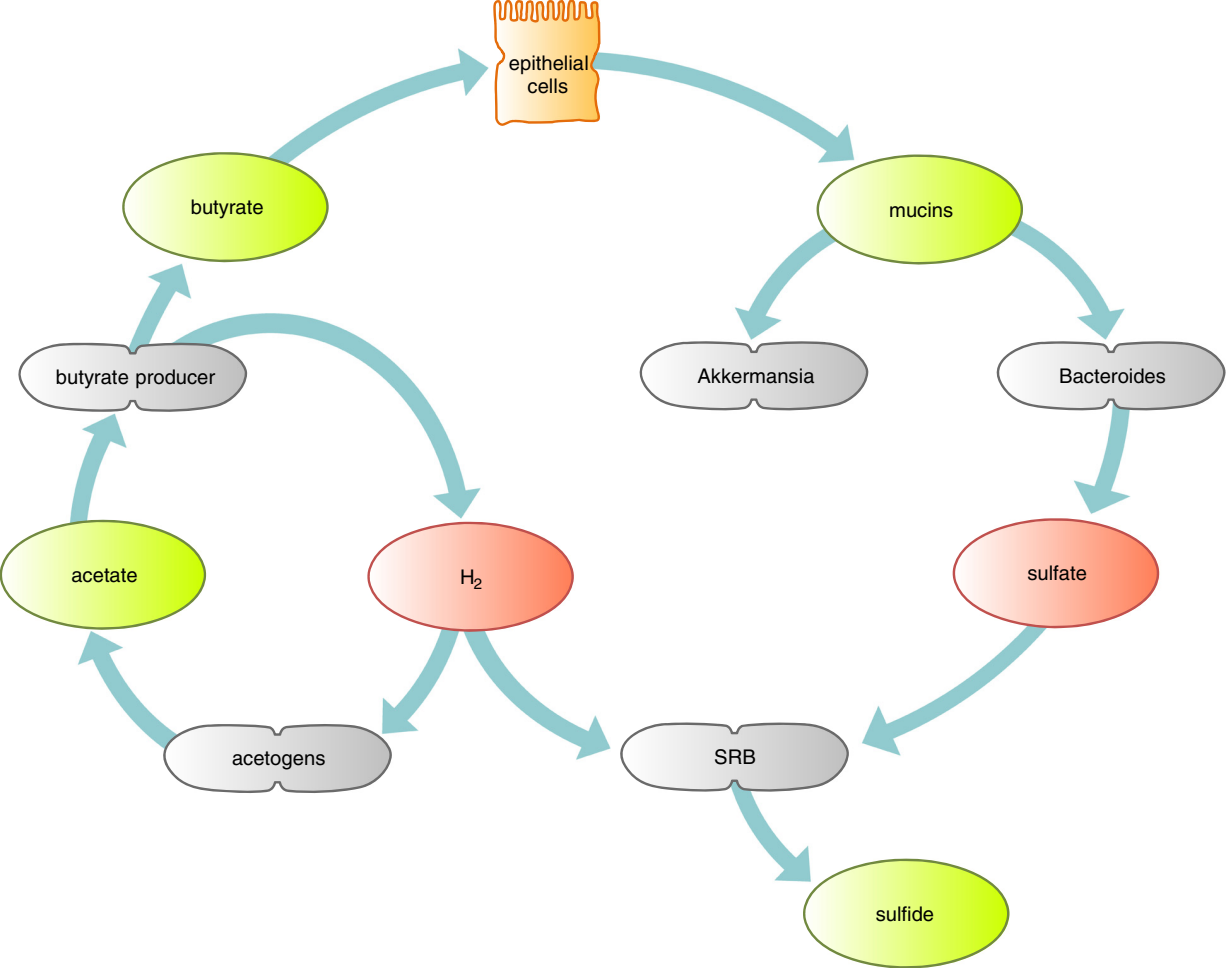
Model for the indirect influence of *Bacteroides* or *Akkermansia* on butyrate production. Incomplete degradation of mucins in microbial communities dominated by *Bacteroides* leads to increased levels of sulfate. In contrast, *Akkermansia* can fully degrade mucins and use sulfate in an assimilatory manner. Concerning hydrogenotrophs, acetogens might outperform sulfate-reducing bacteria in microbial communities with increased *Akkermansia* abundances. Furthermore, acetate production by acetogens enhances butyrate production via co-fermentation of acetate (but-pathway)

Turning away from such direct dietary effects, community C3 dissects alterations with respect to the risk for developing anti-islet autoantibodies. There is a significant increase of autoantibody positive cases in subgroup G33 of children showing a community pattern with increased abundances of *Bacteroides* (Fig. 2f, b). Considering progression to autoimmunity, the effect becomes even more pronounced (Fig. 3a). Linking these observations to dietary patterns, the number of children exposed to complex diet (i.e., with five food components) is higher in subgroup G33 compared to G31 and G32 (Fig. 2c). Additionally, *Bacteroides* abundances were significantly increased in children that had meat in their diet, an observation which has also been made in another study [40]. Overall, we support observations made in the prospective cohorts, that early introduction of higher food complexity increases the risk for autoimmunity [23–25, 41]. Asking what compensates for lower abundances of *Bacteroides* in subgroups G31 and G32, it is striking that both subgroups have higher levels of *Akkermansia* (Fig. 2e). *Akkermansia muciniphila* is characterized by its potential to grow on mucin [42]. Within this habitat, *Akkermansia* markedly represents a specialist with a genome containing a high number of enzymes for degradation of human-derived mucins [43]. Unlike *Bacteroides* species, which are also able to utilize mucins, *Akkermansia* cannot switch to carbohydrate fermentation derived from luminal content [42, 44]. Generally, higher abundances of *Akkermansia* have been associated with a healthy gut community in several studies [45, 46]. Most mucin degraders such as *Clostridium*, *Bifidobacterium*, and *Bacteroides* are not able to fully degrade mucins [47]. Therefore, mucin-degrading bacteria are frequently associated with sulfate-reducing bacteria (SRB), which use sulfate for gain of energy, thereby releasing sulfide [47]. Indeed, mucin fermentation in the colon has been demonstrated to increase the amount of released sulfate [47]. In contrast, *Akkermansia* are able to fully degrade mucins and its genetic content enables assimilatory utilization of sulfate [48]. Functionally, SRB as well as methanogens compete with acetogens such as *Blautia* and *Ruminococcus* for H_2_ produced during carbohydrate fermentation. Since hydrogen inhibits further production of short chain fatty acids (SCFAs) [39], the hydrogen gradient is a sensible driver for SCFA balance. In the presence of sulfate, SRB would typically outperform methanogens and acetogens in H_2_ utilization [49]. On the other side, H_2_ utilization by acetogens raises levels of acetate which then fosters higher efficiency in butyrate production via the but-pathway [38]. Butyrate has several health promoting effects. First of all, it is the major energy source for intestinal epithelial cells [50, 51]. Consequently, butyrate has been shown to increase mucus production [52, 53]. Indeed, we observed that in contrast to children in the *Bacteroides*-dominated subgroup (G33), children in the *Akkermansia*-dominated subgroups (G31 and G32) harbor a microbiome that seems to prefer butyrate-production through co-fermentation of acetate (Fig. 3d). Thus, our data leads to the hypothesis (Fig. 4) that alterations in the ensemble of mucin degraders, *Bacteroides* and *Akkermansia*, directly affect competition among hydrogenotrophic bacteria, which indirectly leaves an impact on butyrate production. Due to decreased available sulfate levels in *Akkermansia*-dominated communities, acetogens might have an indirect advantage which then leads to a butyrogenic effect via the but-pathway. Closing the argument towards an increased risk for anti-islet autoimmunity, there are several hypotheses currently discussed. Lower butyrate availability might impair gut integrity, thus allowing larger molecules to penetrate the epithelial barrier (leaky gut hypothesis). This line of thought frames within the context of the perfect storm hypothesis and hygiene hypothesis [12, 13]. On the other hand, butyrate might also directly modulate immune function, in particular inflammation [50]. A third option lies in the capability of co-evolved gut inhabitants, in particular *Bacteroides* spp. to trigger immune signals of the host in order to defend their ecological niche [54].

Considering the host, dietary patterns clearly impact the ecological balance just discussed. Breast-feeding, for example, prepares the gut ecosystem for later colonization with mucin-degrading specialists [55], since human milk oligosaccharides (HMO) are similar in protein structure to mucins produced by gut epithelial cells [56]. It therefore seems likely that through an early introduction of complex carbohydrates, the ecological advantage for *Akkermansia* provided by HMO might be compromised, in favor of other mucin degraders, which can also feed on alternative substrates as, e.g., resistant starch. Being the dominant end-product of lactate fermentation in the human intestinal communities [57], acetate provides a main source of butyrate production [58, 59]. Note that it was shown that milk-dominated diets support the butyrogenic effect of co-colonization of *Bifidobacterium* with acetate utilizers [60].

In silico analysis of 16S rRNA gene fragments derived from SRBs revealed a high number of unclassified sequences at genus level within the different groups of sulfate reducers deposited in the databases (data not shown). Thus, the majority of SRB could not be resolved on genus level due to under-representation in OTU databases. Thus, we can only provide indirect support concerning the role of sulfate in shaping the hydrogenotrophic community. A diet rich in meat has been shown to increase exogenous sulfide in feces [61]. Thus, our observation of an association between meat consumption and increased abundance of *Bacteroides* is in line with the part of the hypothesis claiming that increased abundances of *Bacteroides* may come along with higher levels of sulfate being available in the system, thereby shifting the hydrogenotrophic community towards sulfide-releasing SRBs. Note that the presence of *B. thetaiotaomicron* had significant impact on the growth of SRB in an animal study [62].

The observation of increased abundances of *Bacteroides* preceding anti-islet autoimmunity shows striking parallels with other autoimmune diseases, in particular Celiac disease (CD) [63]. De Palma et al. [64] associated an HLA-DQ2 genotype being associated with both, increased risk for CD and T1D with higher abundances in the *Bacteroides-Prevotella* group in stool samples of children of less than 1 month of age. Of interest for our work is the fact that individuals at higher risk have been reported to harbor significantly higher abundances of SRB [64]. Sánchez et al. [63] analyzed the diversity of *Bacteroides* spp. in greater detail showing that infants with high HLA risk for CD showed higher prevalence of *Bacteroides vulgatus*. Note that in samples from adults, Leitch et al. [65] found *B. vulgatus* to be the only *Bacteroides* species detected growing on mucin. Davis-Richardson et al. [15] also relate increased abundances of *B. dorei*, a close relative of *B. vulgatus*, with increased risk of islet-autoimmunity in the Finnish DIPP cohort. Along with observations made in the Finnish cohort [15], it seems likely that the period determining possible effects of the microbial community on pathogenesis of T1D falls within the first year of life. As can be seen from temporal data, alterations in abundances of *Akkermansia* fade within the second year of life (Fig. 3c). The same holds true for *Bacteroides* (Fig. 3b). It also appears likely that increased abundances of *Bacteroides* in G31 within the first year of life are due to other representatives of the genus than the abundances showing up later at the age of 2 years (Fig. 3b). A characteristic property of the genus *Bacteroides*, following from their long co-evolution with the human host, is the ability to switch from carbohydrate fermentation to digestion of endogenously-derived mucins [66]. Thus, depending on the availability of glycan sources in the lumen, *Bacteroides* community composition is expected to vary.

Overall, the combination of our results with an extensive literature search leads to a refined functional hypothesis explaining a possible role of the gut microbial community in pathogenesis of anti-islet autoimmunity. In summary, these observations support the hypothesis that increased availability of butyrate in the intestinal tract has a protective effect on development of autoimmunity and T1D. Concerning the risk of developing anti-islet autoantibodies and/or T1D several studies associated an increased risk with early introduction of complex food [10, 23–25] while protective effects of breast-feeding are discussed controversially [10, 22, 23]. The influence of diet, in particular breast-feeding on microbial composition in the large intestine is well documented. Basically, breast-feeding as well as formula-feeding seems to associate with higher abundances of lactic acid bacteria and *Bifidobacterium* spp. [67, 68]. Nevertheless, the overall net-effect with respect to the development of disease in our hypothesis depends on cross-feeding effects between mucin degraders, hydrogenotrophs, and butyrate producers. Thus, breast-feeding alone might or might not alter the risk, depending on the composition of the microbial ensemble. With the effect of diet on the gut microbial community being profound, evaluations of the role of the microbiome in host-microbial homeostasis and in particular its association with autoimmunity should indispensably take the nutritional habits of the subject, i.e., the dietary age into account. This conclusion is of peculiar importance for the period between birth and 3 years of age, where dramatic changes in diet leave their trace on the developing microbial ecosystem. The proposed approach using community detection in association networks of bacteria provides a basis for analyzing such effects on a level beyond the single bacterium and the whole community.

## Conclusions

Based on a novel approach for the identification of microbial communities from co-occurrence networks, we provide a functional model for the association of gut microbial communities and the development of early anti-islet cell autoantibodies. Compared to *Akkermansia*-dominated subgroups of children, *Bacteroides*-dominated subgroups were associated with early autoantibody development and decreased potential of butyrate production via the co-fermentation of acetate. The data suggest that differences in mucin degradation capabilities between the generalist *Bacteroides* and the specialist *Akkermansia* may lead to shifts in the abundances of acetogens vs. sulfate-reducing bacteria. Due to incomplete degradation of mucins in *Bacteroides*-dominated communities, sulfate-reducing bacteria might have a competitive advantage compared to acetogens regarding the removal of hydrogen. In contrast, in *Akkermansia*-dominated communities, abundance of acetogens might be increased due to lower levels of sulfate, leading to increased levels of butyrate via the co-fermentation of acetate. Additionally, early introduction of solid food components, such as meat can provide *Bacteroides* with a competitive advantage as *Bacteroides* is able to switch from endogenous to exogenous nutrient sources. Thus, we provide evidence that butyrate has a protective effect on the development of anti-islet cell autoimmunity and that this effect is associated with differences in composition of mucin-degrading bacteria and the early introduction of complex food. Our model provides a first step into the direction of a functional understanding of the role of the gut microbiome in the pathogenesis of type 1 diabetes. However, this model needs to be validated in larger sample size cohorts including metagenomics data.

### Ethics approval and consent to participate

The study was approved by the ethics committee of the Ludwig-Maximilian-University, Munich, Germany (Ethikkommission der Medizinischen Fakultät der Ludwig-Maximilians Universität No. 329/00).

### Consent to publish

Written informed consent was obtained from the parents.

### Availability of supporting data

The 16S sequences supporting the results of this article is available in NCBI’s short read archive (SRP063271; http://www.ncbi.nlm.nih.gov/Traces/study/?acc=SRP063271).

## Additional files

Additional file 1: Full documentation of all statistical analyses. (PDF 585 kb) Additional file 2: OTU abundance table of all 298 stool samples. (TXT 699 kb) Additional file 3: KO abundance table at age 0.5+/−0.25 years. (TXT 953 kb) Additional file 4: Figures S1–S4. (PDF 1876 kb) Additional file 5: Table S1, stratifications of children based on abundances in communities C1, C2 and C3. (PDF 27 kb)

## Abbreviations

buk: phosphotransbutyrylase and butyrate kinase
but: butyryl CoA:acetate CoA transferase
CD: celiac disease
HLA: human leukocyte antigen
HMO: human milk oligosaccharide
IQR: interquartile range
OTU: operational taxonomic unit
PCoA: principal coordinate analysis
rRNA: ribosomal ribonucleic acid
SCFA: short chain fatty acid
SRB: sulfate reducing bacteria
T1D: type 1 diabetes
