# Supplementary material for: Towards a functional hypothesis relating anti-islet cell autoimmunity to the dietary impact on microbial communities and butyrate production
Source: Microbiome. 2016 Apr 26;4:17. doi: 10.1186/s40168-016-0163-4 (PMC4845316; doi:10.1186/s40168-016-0163-4)
Supplement: Additional file 1: — Full documentation of all statistical analyses. (PDF 585 kb) [file 40168_2016_163_MOESM1_ESM.pdf]

Towards a functional hypothesis relating anti-islet cell autoimmunity to the dietary impact on microbial communities and butyrate production (knitr documentation)

David Endesfelder et al.

10th of March 2016

Load required R packages

Here we load all reqired R packages into R.

```
library(WriteXLS)
library(igraph)
library(gdata)
library(vegan)
library(ape)
library(cluster)
library(fpc)
library(survival)
library(beeswarm)
library(ccrepe)
```

Functions

The following function is used to calculate UniFrac distances. The function is a modified version of the R package GUniFrac.

```
unifrac2 <- function(otu.tab, tree)
{
    if (!is.rooted(tree))
        stop("Rooted phylogenetic tree required!")
    otu.tab <- as.matrix(otu.tab)
    n <- nrow(otu.tab)
    if (is.null(rownames(otu.tab))) {
        rownames(otu.tab) <- paste("comm", 1:n, sep = "_")
    }
    unifracs <- array(NA, c(n, n), dimnames = list(rownames(otu.tab),
        rownames(otu.tab)))
   for (j in 1:n) {
      unifracs[j, j] <- 0
        }

    if (sum(!(colnames(otu.tab) %in% tree$tip.label)) != 0) {
        stop("The OTU table contains unknown OTUs! OTU
              names\n\t\t\t\t\tin the OTU table
              and the tree should match!")
```

```
    }
    absent <- tree$tip.label[!(tree$tip.label %in% colnames(otu.tab))]
    if (length(absent) != 0) {
        tree <- drop.tip(tree, absent)
        warning("The tree has more OTU than the OTU table!")
    }
    tip.label <- tree$tip.label
    otu.tab <- otu.tab[, tip.label]
    ntip <- length(tip.label)
    nbr <- nrow(tree$edge)
    edge <- tree$edge
    edge2 <- edge[, 2]
    br.len <- tree$edge.length
    cum <- matrix(0, nbr, n)

    for (i in 1:ntip) {
        tip.loc <- which(edge2 == i)
        cum[tip.loc, ] <- cum[tip.loc, ] + otu.tab[, i]
        node <- edge[tip.loc, 1]
        node.loc <- which(edge2 == node)
        while (length(node.loc)) {
            cum[node.loc, ] <- cum[node.loc, ] + otu.tab[, i]
            node <- edge[node.loc, 1]
            node.loc <- which(edge2 == node)
        }
    }
    for (i in 2:n) {
        for (j in 1:(i - 1)) {
            cum1 <- cum[, i]
            cum2 <- cum[, j]
            ind <- (cum1 + cum2) != 0
            cum1 <- cum1[ind]
            cum2 <- cum2[ind]
            br.len2 <- br.len[ind]
            diff <- abs(cum1 - cum2)/(cum1 + cum2)
              w <- br.len2 * (cum1 + cum2)
            unifracs[i, j] <- unifracs[j, i] <- sum(diff * w)/sum(w)
        }
    }
    return(unifracs)
}
```

The following functions are used for Kaplan-Meier analyses.

```
survplot <- function (x, data = NULL, subset = NULL, snames, stitle, col,
    lty, lwd, show.n.risk = TRUE, hr.pos = "topright", legend.pos = "bottomleft",
    ...)
{
    eval(bquote(s <- survfit(x, data = data, subset = .(substitute(subset)))))
    if (missing(stitle))
        stitle <- strsplit(deparse(x), " ~ ")[[1]][2]
    if (missing(snames)) {
        snames <- names(s$strata)
```

```
        prefx <- paste(strsplit(deparse(x), " ~ ")[[1]][2], "=",
            sep = "")
        if (all(substr(snames, 1, nchar(prefx)) == prefx)) {
            snames <- substr(snames, nchar(prefx) + 1, 100)
        }
    }
    ns <- length(s$strata)
    stopifnot(length(snames) == ns)
    if (show.n.risk) {
        mar <- par("mar")
        mar[1] <- mar[1] + ns + 2
        opar <- par(mar = mar)
        on.exit(par(opar))
    }
    if (missing(col))
        col <- 1:ns
    if (missing(lty))
        lty <- par("lty")
    if (missing(lwd))
        lwd <- par("lwd")
    plot(s, col = col, lty = lty, lwd = lwd, ...)
    if (!is.na(legend.pos)) {
        legend(legend.pos, legend = snames, title = stitle, col = col,
            lty = lty, lwd = lwd, bty = "n")
    }
    if (ns == 2) {
        eval(bquote(cox <- summary(coxph(x, data = data, subset = .(substitute(subset))))))
        hr <- format(cox$conf.int[1, c(1, 3, 4)], digits = 2)
        p <- format(cox$sctest[3], digits = 2)
        txt1 <- paste("HR = ", hr[1], " (", hr[2], " - ", hr[3],
            ")", sep = "")
        txt2 <- paste("logrank P =", p)
        legend(hr.pos, legend = c(txt1, txt2), bty = "n")
    }
    if (show.n.risk) {
        xticks <- axTicks(1)
        m <- nrisk(s, xticks)
        for (i in 1:ns) {
            axis(1, at = xticks, labels = m[i, ], line = 4 +
                i, tick = FALSE, col.axis = col[i])
        }
        title(xlab = "number at risk", line = 5)
    }
}

########################################

censor <- function (x, at)
{
    stopifnot(class(x) == "Surv")
    stopifnot(attr(x, "type") == "right")
    wh <- x[, "time"] > at
    x[wh, "time"] <- at
```

```
    x[wh, "status"] <- 0
    x
}

#############################################

nrisk <- function (x, times = pretty(x$time))
{
    stopifnot(class(x) == "survfit")
    ns <- length(x$strata)
    idx <- rep.int(1:ns, x$strata)
    str.n.risk <- split(x$n.risk, idx)
    str.times <- split(x$time, idx)
    m <- sapply(times, function(y) {
        sapply(1:ns, function(i) {
            w <- which(str.times[[i]] >= y)[1]
            ifelse(is.na(w), 0, str.n.risk[[i]][w])
        })
    })
    rownames(m) <- names(x$strata)
    colnames(m) <- times
    m
}
```

The following function is used to calculate the running average for plotting of timeseries.

```
fun.get.data.window <- function(data, age, clusters, clust.num
                               , window = 0.5, sequence = seq(0.5, 3, 0.01)
                               , genus){

    samples <- names(clusters)[clusters == clust.num]
    data.cluster <- data[rownames(data) %in% samples, ]
    age.cluster <- age[rownames(data) %in% samples]
    mean.window <- rep(NA, length(sequence))
    sd.window <- rep(NA, length(sequence))
    bact <- data.cluster[, genus]
    count <- 1
    for(i in sequence){
        tmp.bact <- bact[age.cluster >= i-window/2 & age.cluster <= i+window/2]
        vect.window <- rep(NA, length = length(unique(names(tmp.bact))))
        names(vect.window) <- unique(names(tmp.bact))
        for(k in unique(names(tmp.bact))){
            sub.bact <- tmp.bact[names(tmp.bact) %in% k]
            if(length(sub.bact) > 1){
                vect.window[k] <- median(sub.bact)
            }else{
                vect.window[k] <- sub.bact
            }
        }
        if(length(vect.window) >= 3){
            mean.window[count] <- mean(vect.window, na.rm = TRUE)
            sd.window[count] <- sd(vect.window)/sqrt(length(vect.window))
        }else{
```

```
            mean.window[count] <- NA
            sd.window[count] <- NA
        }
        count <- count + 1
    }
    return(rbind(mean.window, sd.window))

}
```

The following function is used to obtain abundances for a specific age interval (e.g. 0.5+/-0.25 years).

```
fun.get.abundances.time <- function(bact.genus, year, thresh, metadata){

    mat.genus <- matrix(NA, nr = ncol(bact.genus), nc = length(unique(metadata[, "child_ID"])))
    rownames(mat.genus) <- colnames(bact.genus)
    colnames(mat.genus) <-  unique(metadata[, "child_ID"])
    for(i in unique(metadata[, "child_ID"])){
        sub.metadata <- metadata[metadata[, "child_ID"] %in% i, ]
        sub.time <- as.numeric(sub.metadata[, "stool_age"])
        sub.microbiome <- bact.genus[sub.metadata[, "sample_ID"], ]
        delta <- abs(year - sub.time)
        if(min(delta) <= thresh)
            mat.genus[, i] <- sub.microbiome[which.min(delta), ]


    }
    mat.genus <- mat.genus[, colSums(is.na(mat.genus)) != nrow(mat.genus)]
    return(mat.genus)
}
```

Data preparation

The Greengenes 13.8 tree has been updated for use on genus level. Here, we load the tree into R for the calculation of UniFrac distances on genus level.

```
load("H:/knitr/Data/tree.genus.RData")
```

In this section we read the full OTU table (see Additional file 4), the metadata (all required information can be found in Additional file 2, Supplementary Table 1) and KO table (see Additional file 5) at age 0.5+/-0.25 years and the taxonomic (Greengenes 13.8) information into R.

```
metadata.early <- as.matrix(read.table("H:/knitr/Data/metadata.early.txt"
                , sep = "\t", header = TRUE))

colnames(metadata.early) <- c("stool_age", "Case/Control", "seroconversion_age"
                              , "max_sample_age" , "Breast feeding(any)"
                              , "formula", "potato", "vegetable", "fruit",  "meat")

otu.data <- as.matrix(read.table("H:/knitr/Data/otu.data.txt", sep = "\t", header = TRUE)   )
metadata <- otu.data[, c("child_ID",   "stool_age")]
metadata <- cbind(rownames(metadata), metadata)
colnames(metadata)[1] <- "sample_ID"
```

```
otu.data <- otu.data[, -c(1, 2)]
colnames(otu.data) <- gsub("X", "", colnames(otu.data))
class(otu.data) <- "numeric"
otu.data <- t(otu.data)

taxonomy <- readLines("H:/knitr/Data/97_otu_taxonomy_13_8.txt")

ko.data.early <- as.matrix(read.table(file = "H:/knitr/Data/ko.data.early.txt"
                        , sep = "\t", header = TRUE))
```

In this section, the taxonomic information is pre-processed to obtain a matrix where columns are represented by taxonomic levels and rows represent OTUs from the Greengenes 13.8 database.

```
taxonomy <- sapply(taxonomy, function(x) strsplit(x, "\t"))
taxonomy <- matrix(unlist(taxonomy), nr = length(unlist(taxonomy))/2, nc = 2, byrow = TRUE)
taxonomy.table.13_8 <- t(sapply(taxonomy[, 2], function(x) strsplit(x, "; ")[[1]]))
rownames(taxonomy.table.13_8) <- taxonomy[, 1]
colnames(taxonomy.table.13_8) <- c("Root", "Phylum", "Class"
                                   , "Order", "Family", "Genus", "Species")

taxonomy.table.13_8 <- apply(taxonomy.table.13_8, 2, function(x, start = 4)
    return(substr(x, start, nchar(x))))
taxonomy.table.13_8 <- taxonomy.table.13_8[taxonomy.table.13_8[, "Genus"] != "", ]
taxonomy.table.13_8 <- taxonomy.table.13_8[, 1:6]
taxonomy.table.13_8[grep("]", taxonomy.table.13_8[, "Genus"]), "Genus"] <-
      substr(taxonomy.table.13_8[grep("]", taxonomy.table.13_8[, "Genus"]), "Genus"], 2,
             nchar(taxonomy.table.13_8[grep("]", taxonomy.table.13_8[, "Genus"]), "Genus"]) - 1)
taxonomy.table.13_8[grep("]", taxonomy.table.13_8[, "Family"]), "Family"] <-
    substr(taxonomy.table.13_8[grep("]", taxonomy.table.13_8[, "Family"]), "Family"], 2,
           nchar(taxonomy.table.13_8[grep("]", taxonomy.table.13_8[, "Family"]), "Family"]) - 1)
taxonomy.table.13_8[taxonomy.table.13_8 == ""] <- "unclassified"
```

In this section, the OTU table and the taxonomic information is used to obtain a matrix containing abundances on genus level.

```
genus <- taxonomy.table.13_8[, "Genus"]
mat.genus.all <- sapply(unique(genus), function(x){
            tt <- names(genus)[genus == x]
            tt <- intersect(tt, rownames(otu.data))
            if(length(tt) >= 1)
                return(colSums(otu.data[tt, ,drop = F]))
             })

mat.genus.all <- simplify2array(mat.genus.all[!sapply(mat.genus.all, is.null)])
mat.genus.timeseries <- sweep(mat.genus.all, MARGIN = 1
                              , FUN = "/", rowSums(mat.genus.all, na.rm = TRUE))
```

Here, we obtain the matrix with relative abundances on genus level for the age interval 0.5+/-0.25 years. For each child, at most one probe is used in this interval. If several probes of a child are available in this interval, the probe closest to 0.5 years is used. Low abundant reads are excluded as described in Materials and Methods.

```
mat.genus.early <- fun.get.abundances.time(mat.genus.all
                                         , year = 0.5, thresh = 0.25, metadata = metadata)
mat.genus.early <- t(mat.genus.early)
mat.genus.early.portion <-
  mat.genus.early[, colSums(mat.genus.all
                            , na.rm = TRUE)/sum(colSums(mat.genus.all, na.rm = TRUE))*100 > 0.01]
mat.genus.early.portion <- mat.genus.early.portion[, colSums(mat.genus.early.portion) != 0]
mat.genus.early.portion <- sweep(mat.genus.early.portion, MARGIN = 1
                                 , FUN = "/", rowSums(mat.genus.early.portion, na.rm = TRUE))
```

Estimation of the adjacency matrix

In this section, the adjacency matrix is estimated based on the CCREPE method. An edge is defined if P<0.05 and Spearman's rank correlation > 0.4. The resulting adjacency matrix is stored in an Excel file which is then imported into MATLAB for the estimation of microbial communities from co-occurrence networks. The required MATLAB code can be found at the end of this document.

```
ccrepe.output.13_8 <- ccrepe(mat.genus.early.portion
                           , iterations = 1000
                           , min.subj = 10
                           , sim.score.args = list(method = "spearman"
                                                   , use = "complete.obs")
                           , errthresh = 1e-2)

p.ccrepe.output.13_8 <- ccrepe.output.13_8$p.values
p.ccrepe.output.13_8[is.na(p.ccrepe.output.13_8)] <- 1
scores.ccrepe.output.13_8   <- ccrepe.output.13_8$sim.score
scores.ccrepe.output.13_8[is.na(scores.ccrepe.output.13_8)] <- 0

adj.ccrpe.13_8 <- matrix(0, nr = nrow(p.ccrepe.output.13_8)
                            , nc = ncol(p.ccrepe.output.13_8))
rownames(adj.ccrpe.13_8) <- colnames(adj.ccrpe.13_8) <- rownames(p.ccrepe.output.13_8)
adj.ccrpe.13_8[p.ccrepe.output.13_8 < 0.05 & scores.ccrepe.output.13_8 > 0.4] <- 1

tmp.adj <- adj.ccrpe.13_8
tmp.adj <- as.data.frame(tmp.adj)
# write adjacency matrix into excel file for community detection in MATLAB
WriteXLS("tmp.adj", "H:/knitr/Data/adj.matrix.ccrepe.13_8.xls")
```

Figure 1

Community membership has been estimated in MATLAB and stored in an Excel file ("network_combined_early__13_8.xls""). This file is now imported into R, to plot the network (Figure 1A) and to perform further statistical analysis based on microbial communities on genus level. For plotting, the R package igraph is used.

```
communities.schaub <- read.xls("H:/knitr/Data/network_combined_early_13_8.xls"
                              , header = F)
communities.schaub <- as.matrix(communities.schaub)
rownames(communities.schaub) <- rownames(adj.ccrpe.13_8)
tmp.communities <- communities.schaub
```

```r
communities.schaub[rowSums(adj.ccrpe.13_8) <= 1, 1 ] <- -1
graph.combined.early <- graph.adjacency(adj.ccrpe.13_8, mode = "undirected")
#layout.combined.early <- layout.fruchterman.reingold(graph.combined.early)
#save(layout.combined.early
#      , file = "H:/Microbiom/Picrust_Analysis/RObjects/layout.combined.early.RData")
load("H:/Microbiom/Picrust_Analysis/RObjects/layout.combined.early.RData")

nam <- rep(NA, nrow(communities.schaub))
nam[rownames(communities.schaub) == "Bacteroides"] <- "Bacteroides"
nam[rownames(communities.schaub) == "Veillonella"] <- "Veillonella"
nam[rownames(communities.schaub) == "Akkermansia"] <- "Akkermansia"
nam[rownames(communities.schaub) == "Ruminococcus"] <- "Ruminococcus"
nam[rownames(communities.schaub) == "Bifidobacterium"] <- "Bifidobacterium"
nam[rownames(communities.schaub) == "Streptococcus"] <- "Streptococcus"

plot.igraph(graph.combined.early, layout = layout.combined.early
              , vertex.size = 8, vertex.label = nam, main = "Figure 1A"
                        , vertex.color = c("white", "red"
                                                ,"yellow","blue")[communities.schaub[, 1] + 2]
                        , vertex.label.color = "black", vertex.label.cex = .7, margin = 0
                      , vertex.label.font=2)
```

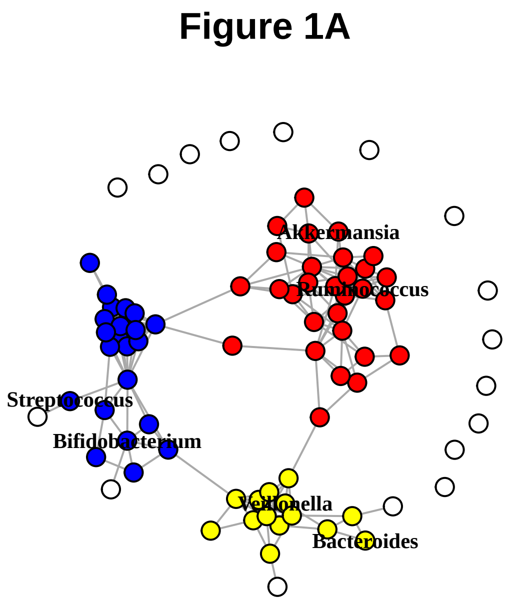

In this section, the taxonomic composition of the three microbial communities is plotted in pie charts (Figure 1B).

```r
genera.C1 <- rownames(communities.schaub)[communities.schaub == 2]
taxonomy.C1 <- unique(taxonomy.table.13_8[taxonomy.table.13_8[, "Genus"] %in% genera.C1, ])
tab.orders.C1 <- table(taxonomy.C1[, "Order"])
tab.phyla.C1 <- table(taxonomy.C1[, "Phylum"])

genera.C2 <- rownames(communities.schaub)[communities.schaub == 0]
taxonomy.C2 <- unique(taxonomy.table.13_8[taxonomy.table.13_8[, "Genus"] %in% genera.C2, ])
tab.orders.C2 <- table(taxonomy.C2[, "Order"])
tab.phyla.C2 <- table(taxonomy.C2[, "Phylum"])

genera.C3 <- rownames(communities.schaub)[communities.schaub == 1]
taxonomy.C3 <- unique(taxonomy.table.13_8[taxonomy.table.13_8[, "Genus"] %in% genera.C3, ])
tab.orders.C3 <- table(taxonomy.C3[, "Order"])
tab.phyla.C3 <- table(taxonomy.C3[, "Phylum"])

all.orders <- unique(c(taxonomy.C1[, "Order"]
                       , taxonomy.C2[, "Order"], taxonomy.C3[, "Order"]))
all.phyla <- unique(c(taxonomy.C1[, "Phylum"]
                      , taxonomy.C2[, "Phylum"], taxonomy.C3[, "Phylum"]))

col.phyla <-  c("red", "magenta","green",  "cyan", "black")
col.tmp <- cbind(col.phyla, all.phyla)
map.order.phylum <- unique(taxonomy.table.13_8[taxonomy.table.13_8[, "Order"] %in%
                                                   all.orders, c("Phylum", "Order")])
map.order.phylum <- map.order.phylum[match(all.orders, map.order.phylum[, "Order"]), ]
col.tmp <- col.tmp[match(map.order.phylum[, "Phylum"], col.tmp[, 2]), ]

par(mfrow = c(2, 2))
par(cex=0.7)
par(mar = c(1, 1, 1, 1))
pie(tab.orders.C1, col = col.tmp[match(names(tab.orders.C1), all.orders), 1]
      , main = "C1", radius = 0.6)
pie(tab.orders.C2, col = col.tmp[match(names(tab.orders.C2), all.orders), 1]
      , main = "C2", radius = 0.6)
pie(tab.orders.C3, col = col.tmp[match(names(tab.orders.C3), all.orders), 1]
      , main = "C3", radius = 0.6)
plot.new()
legend("center", all.phyla, col = col.phyla, pch = 15, title = "Phylum")
```

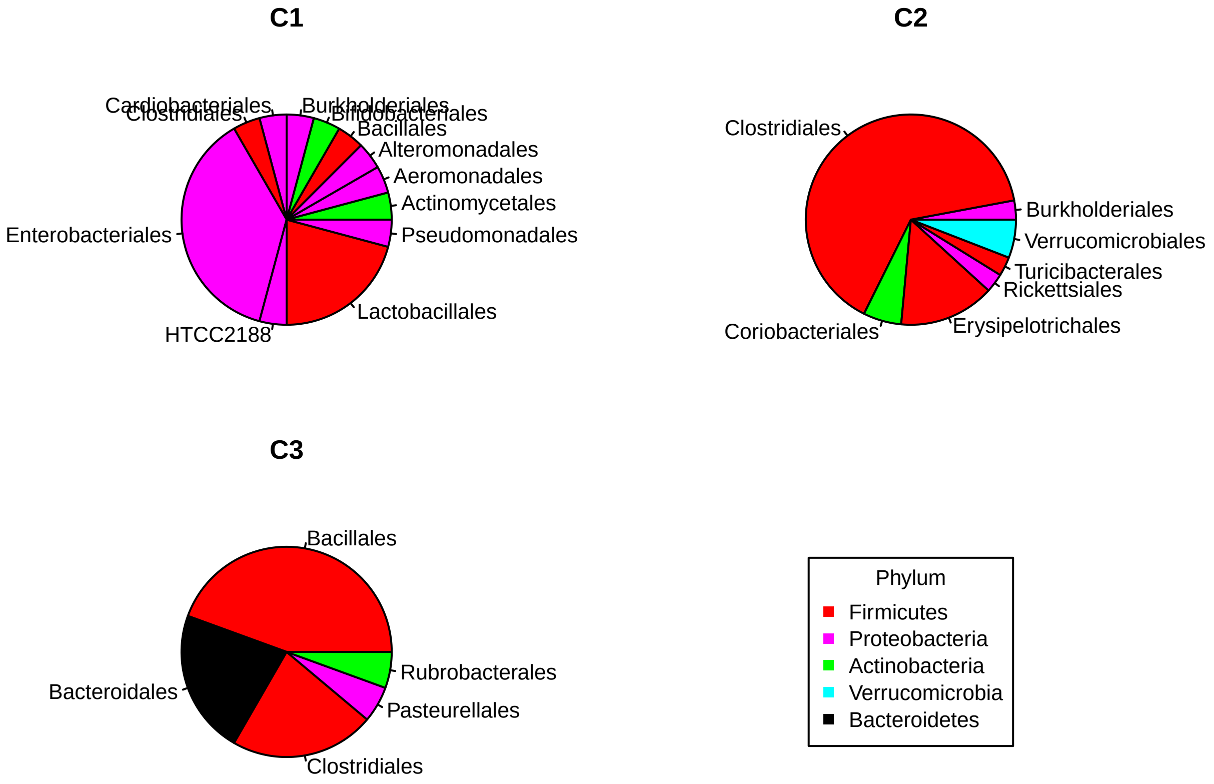

Here we calculate p-values, comparing the taxonomic composition between microbial communities. P-values are calculated based on two-sided Fisher's exact tests.

```
p.values.taxonomy.C1 <- rep(NA, length(all.orders))
names(p.values.taxonomy.C1) <- all.orders

for(k in all.orders){
    num.orders <- c(sum(taxonomy.C1[, "Order"] == k), sum(taxonomy.C1[, "Order"] != k)
            , sum(taxonomy.C2[, "Order"] == k) + sum(taxonomy.C3[, "Order"] == k)
            , sum(taxonomy.C2[, "Order"] != k) + sum(taxonomy.C3[, "Order"] != k))
    p.values.taxonomy.C1[k] <- signif(fisher.test(matrix(num.orders,
                                                   2, 2, byrow = TRUE))$p.value, 3)

}
print(sort(p.values.taxonomy.C1[p.values.taxonomy.C1 < 0.05]))
```

```
## Enterobacteriales     Clostridiales   Lactobacillales
##          9.18e-06          6.46e-05          2.30e-03
```

```
p.values.taxonomy.C2 <- rep(NA, length(all.orders))
names(p.values.taxonomy.C2) <- all.orders

for(k in all.orders){
    num.orders <- c(sum(taxonomy.C2[, "Order"] == k), sum(taxonomy.C2[, "Order"] != k)
            , sum(taxonomy.C1[, "Order"] == k) + sum(taxonomy.C3[, "Order"] == k)
```

```r
            , sum(taxonomy.C1[, "Order"] != k) + sum(taxonomy.C3[, "Order"] != k))
    p.values.taxonomy.C2[k] <- signif(fisher.test(matrix(num.orders,
                                                         2, 2, byrow = TRUE))$p.value, 3)

}
print(sort(p.values.taxonomy.C2[p.values.taxonomy.C2 < 0.05]))
```

```
##      Clostridiales  Enterobacteriales         Bacillales
##           2.28e-06           3.50e-03           3.50e-03
## Erysipelotrichales
##           1.51e-02
```

```r
p.values.taxonomy.C3 <- rep(NA, length(all.orders))
names(p.values.taxonomy.C3) <- all.orders

for(k in all.orders){
    num.orders <- c(sum(taxonomy.C3[, "Order"] == k), sum(taxonomy.C3[, "Order"] != k)
            , sum(taxonomy.C1[, "Order"] == k) + sum(taxonomy.C2[, "Order"] == k)
            , sum(taxonomy.C1[, "Order"] != k) + sum(taxonomy.C2[, "Order"] != k))
    p.values.taxonomy.C3[k] <- signif(fisher.test(matrix(num.orders,
                                                         2, 2, byrow = TRUE))$p.value, 3)

}
print(sort(p.values.taxonomy.C3[p.values.taxonomy.C3 < 0.05]))
```

```
##    Bacillales Bacteroidales
##      1.82e-05      2.39e-03
```

Supplementary Figure S1

Here, we plot subnetworks for each microbial community separately.

```r
par(mar = c(2, 2, 2, 2))
    graph.C1 <- graph.adjacency(adj.ccrpe.13_8[tmp.communities[, 1] == 2
                                               , tmp.communities[, 1] == 2]
                                , mode = "undirected")
    plot.igraph(graph.C1, layout = layout.combined.early[tmp.communities[, 1] == 2, ]
                , vertex.size = 12, vertex.color = "blue", vertex.label.color = "black"
                , vertex.label.cex = .7, margin = 0, vertex.label.font=2
                , vertex.label = rownames(tmp.communities)[tmp.communities[, 1] == 2])
```

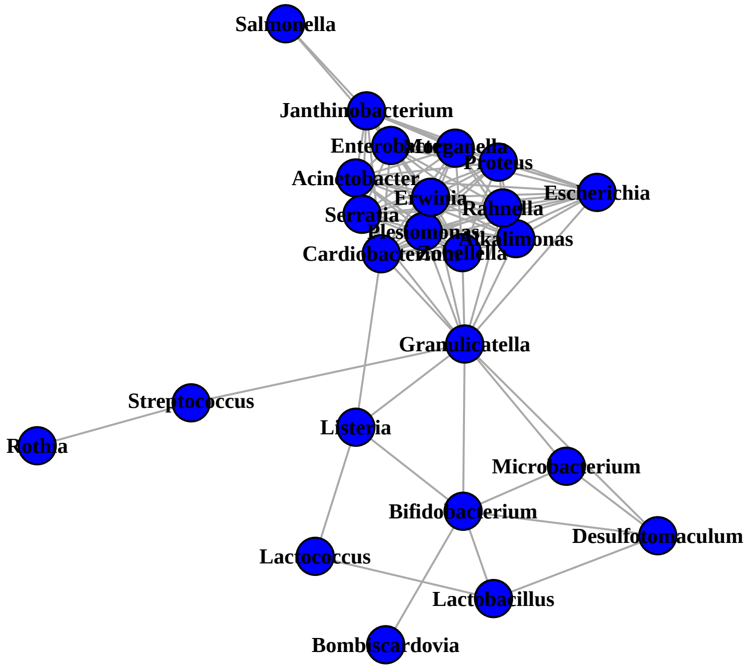


```r
graph.C2 <- graph.adjacency(adj.ccrpe.13_8[tmp.communities[, 1] == 0
                                           , tmp.communities[, 1] == 0]
                            , mode = "undirected")
plot.igraph(graph.C2, layout = layout.combined.early[tmp.communities[, 1] == 0, ]
            , vertex.size = 12, vertex.color = "red", vertex.label.color = "black"
            , vertex.label.cex = .7, margin = 0, vertex.label.font=2
            , vertex.label = rownames(tmp.communities)[tmp.communities[, 1] == 0])
```

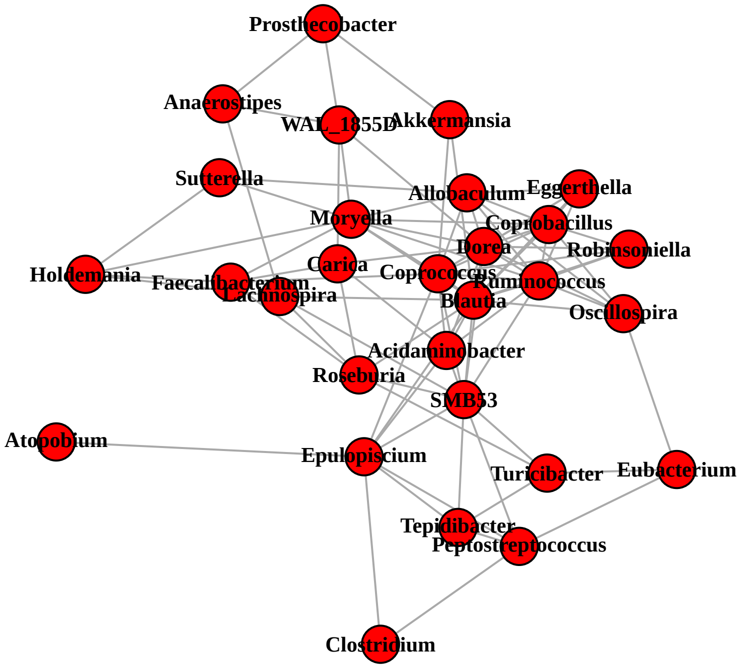


```r
graph.C3 <- graph.adjacency(adj.ccrpe.13_8[tmp.communities[, 1] == 1
                                           , tmp.communities[, 1] == 1]
                            , mode = "undirected")
plot.igraph(graph.C3, layout = layout.combined.early[tmp.communities[, 1] == 1, ]
            , vertex.size = 12, vertex.color = "yellow", vertex.label.color = "black"
            , vertex.label.cex = .7, margin = 0, vertex.label.font=2
            , vertex.label = rownames(tmp.communities)[tmp.communities[, 1] == 1])
```

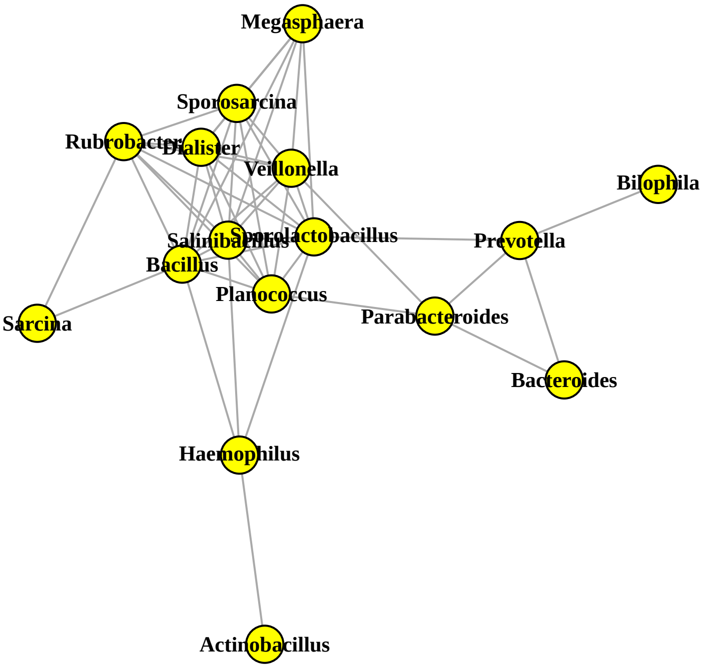

Supplementary Figure S2

Here, we plot the top 6 anundant genera in community C1 over time, using a running average with window size 0.5 years and step size 0.01 years.

```
age <- as.numeric(metadata[, "stool_age"])
genera.C1 <- c("Bifidobacterium", "Streptococcus", "Zobellella"
               , "Erwinia", "Lactobacillus", "Granulicatella")
ss <- seq(0.5, 2, 0.01)
clusters.tmp <- rep(1, length(unique(rownames(mat.genus.timeseries))))
names(clusters.tmp) <- unique(rownames(mat.genus.timeseries))
par(mfrow = c(1, 2))
par(las = 1)
for(genus in genera.C1){
  genus.window <- fun.get.data.window(data = mat.genus.timeseries
                                     , age = age
                                     , clusters = clusters.tmp
                                     , clust.num = 1
                                     , window = 0.5
                                     , sequence = ss
                                     , genus = genus)
  maxima <- max(genus.window[1, ] + genus.window[2, ], na.rm= TRUE)

  plot(ss, genus.window[1, ], type = "l"
       , ylab = "Mean Relative Abundance", xlab = "Age(years)"
```

```
      , col = "red", main = genus, ylim = c(0, maxima + maxima/100))

  for(kk in 2:length(ss))
          polygon(c(ss[kk-1], ss[kk-1], ss[kk], ss[kk])
                  , c(genus.window[1, kk-1] - genus.window[2, kk-1]
                      , genus.window[1, kk-1] + genus.window[2, kk-1]
                      , genus.window[1, kk-1] + genus.window[2, kk]
                    , genus.window[1, kk-1] - genus.window[2, kk])
                  , col = rgb(1, 0, 0, alpha = 0.2), border = NA)
}
```

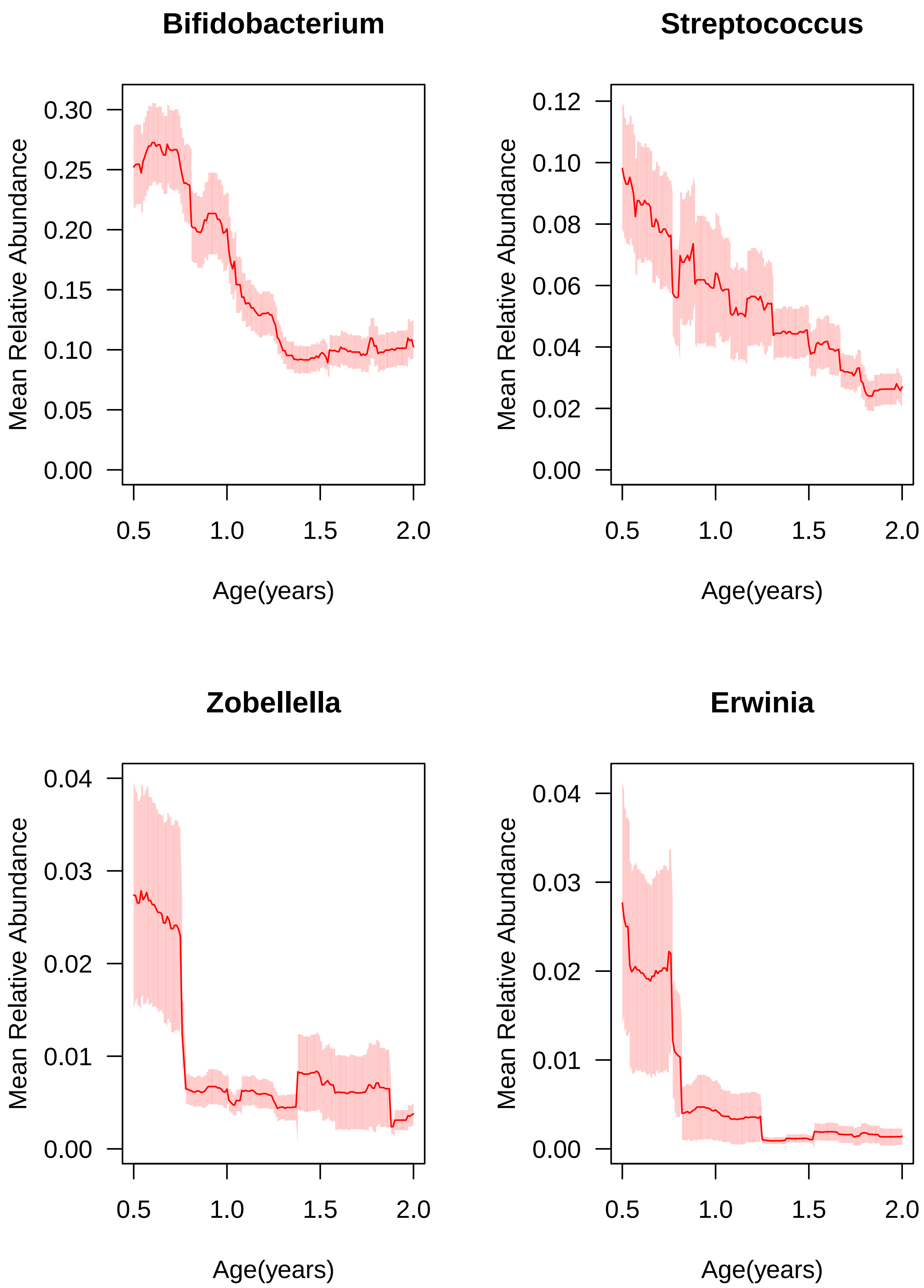
Bifidobacterium
Mean Relative Abundance
0.30
0.25
0.20
0.15
0.10
0.05
0.00
0.5
1.0
1.5
2.0
Age(years)
Streptococcus
Mean Relative Abundance
0.12
0.10
0.08
0.06
0.04
0.02
0.00
0.5
1.0
1.5
2.0
Age(years)
Zobellella
Mean Relative Abundance
0.04
0.03
0.02
0.01
0.00
0.5
1.0
1.5
2.0
Age(years)
Erwinia
Mean Relative Abundance
0.04
0.03
0.02
0.01
0.00
0.5
1.0
1.5
2.0
Age(years)

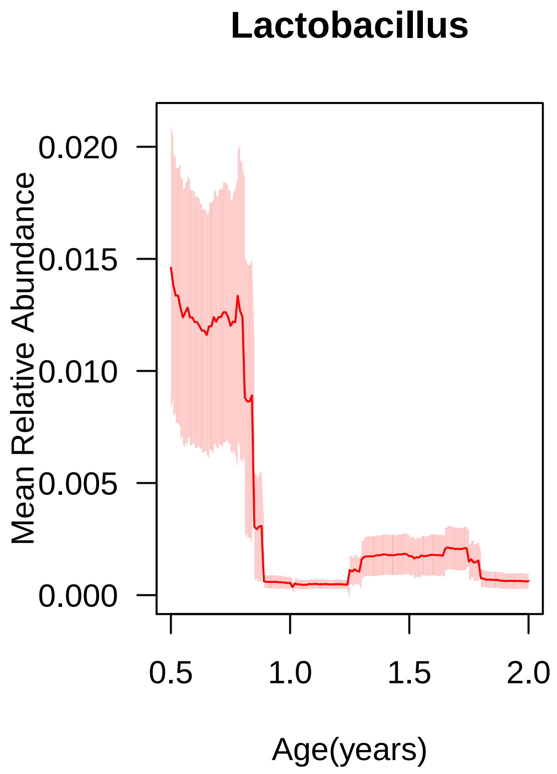

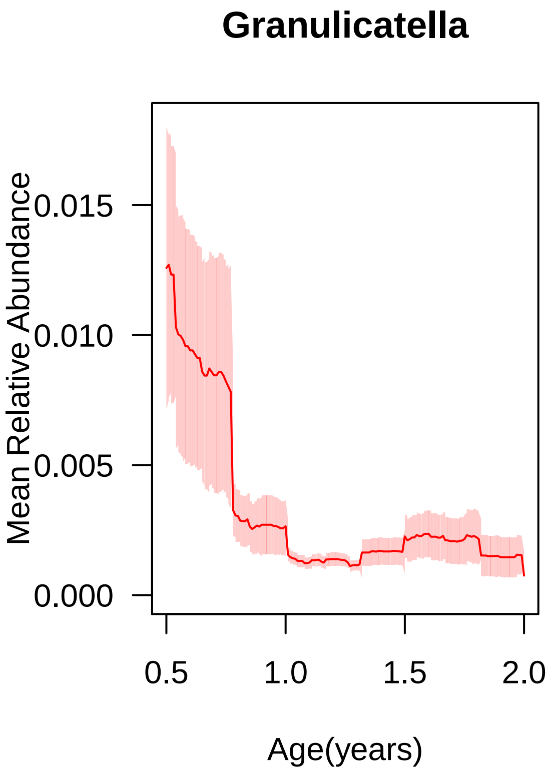

Here, we plot the top 6 anundant genera in community C2 over time, using a running average with window size 0.5 years and step size 0.01 years.

```
genera.C2 <- c("Akkermansia", "Ruminococcus", "Clostridium"
               , "Blautia", "Coprococcus", "Eubacterium")
ss <- seq(0.5, 2, 0.01)
clusters.tmp <- rep(1, length(unique(rownames(mat.genus.timeseries))))
names(clusters.tmp) <- unique(rownames(mat.genus.timeseries))
par(mfrow = c(1, 2))
par(las = 1)
for(genus in genera.C2){
  genus.window <- fun.get.data.window(data = mat.genus.timeseries
                                    , age = age
                                    , clusters = clusters.tmp
                                    , clust.num = 1
                                    , window = 0.5
                                    , sequence = ss
                                    , genus = genus)
  maxima <- max(genus.window[1, ] + genus.window[2, ], na.rm= TRUE)

  plot(ss, genus.window[1, ], type = "l"
       , ylab = "Mean Relative Abundance", xlab = "Age(years)"
       , col = "red", main = genus, ylim = c(0, maxima + maxima/100))

  for(kk in 2:length(ss))
          polygon(c(ss[kk-1], ss[kk-1], ss[kk], ss[kk])
```

```
          , c(genus.window[1, kk-1] - genus.window[2, kk-1]
              , genus.window[1, kk-1] + genus.window[2, kk-1]
              , genus.window[1, kk-1] + genus.window[2, kk]
            , genus.window[1, kk-1] - genus.window[2, kk])
          , col = rgb(1, 0, 0, alpha = 0.2), border = NA)
}
```

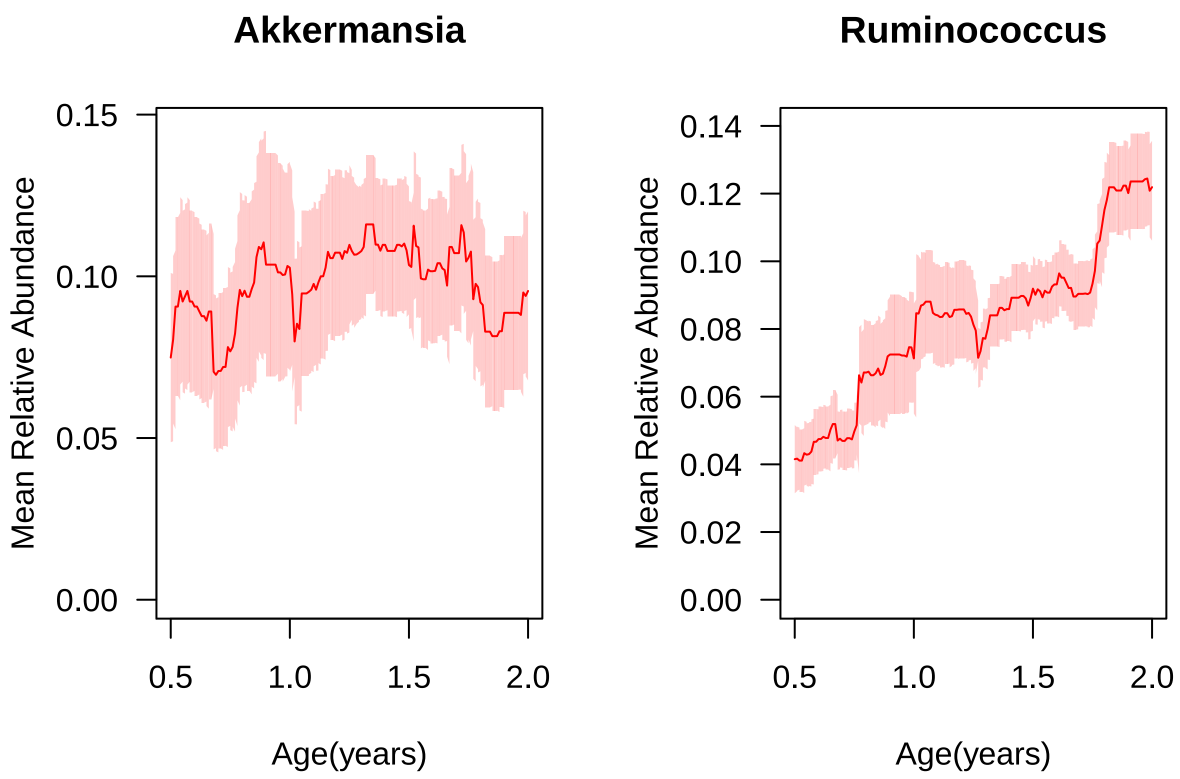

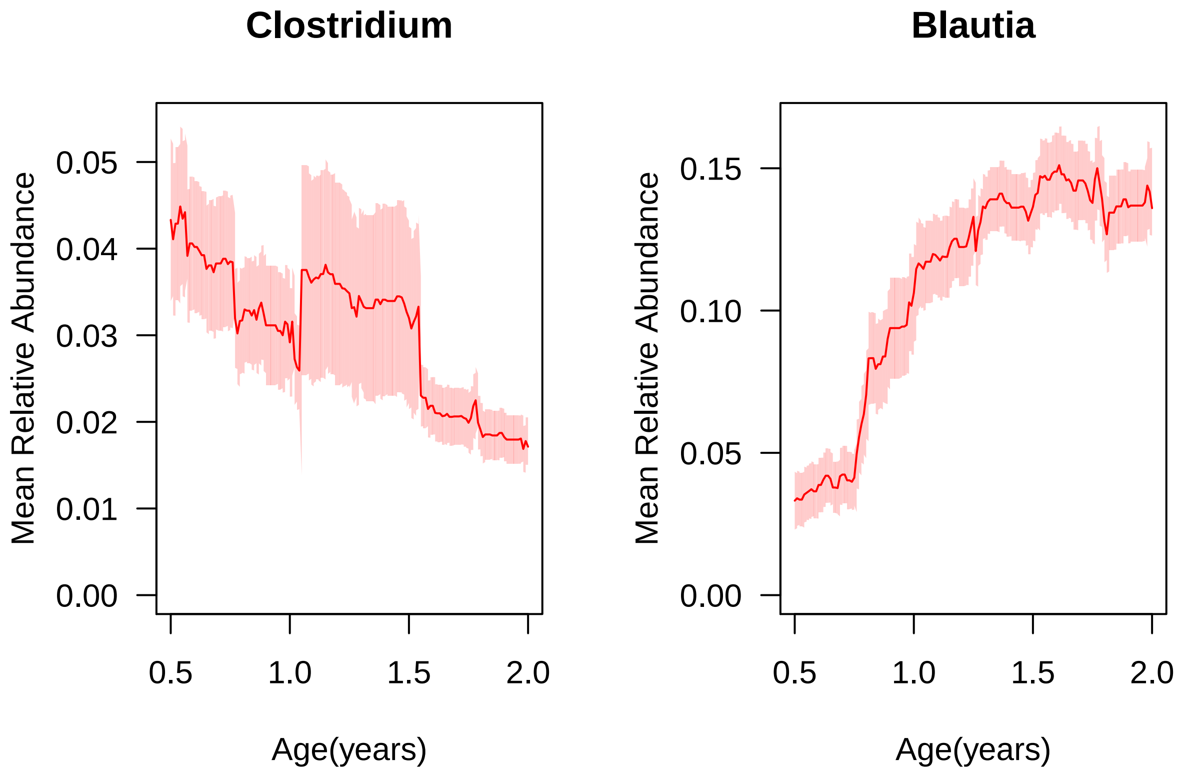
Clostridium
Mean Relative Abundance
0.00
0.01
0.02
0.03
0.04
0.05
0.5
1.0
1.5
2.0
Age(years)
Blautia
Mean Relative Abundance
0.00
0.05
0.10
0.15
0.5
1.0
1.5
2.0
Age(years)

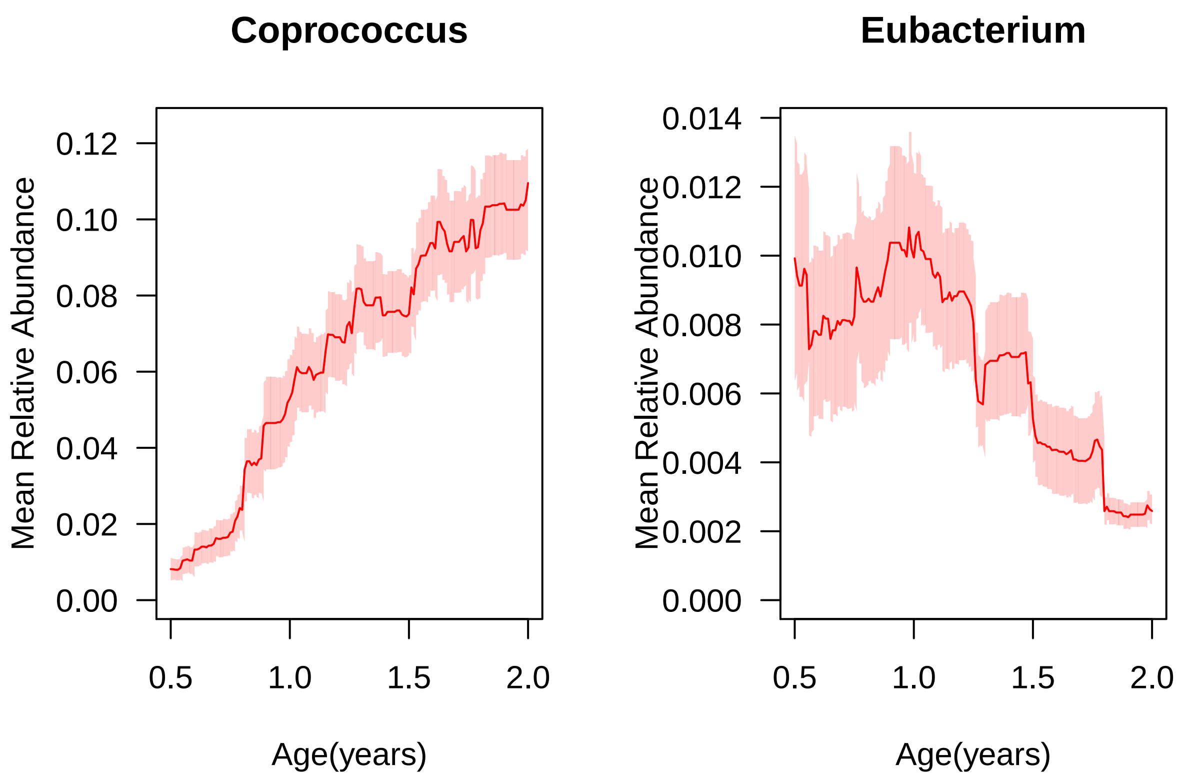

Here, we plot the top 6 anundant genera in community C3 over time, using a running average with window size 0.5 years and step size 0.01 years.

```
genera.C3 <- c("Bacteroides", "Veillonella", "Haemophilus"
               , "Salinibacillus", "Megasphaera", "Prevotella")
ss <- seq(0.5, 2, 0.01)
clusters.tmp <- rep(1, length(unique(rownames(mat.genus.timeseries))))
names(clusters.tmp) <- unique(rownames(mat.genus.timeseries))
par(mfrow = c(1, 2))
par(las = 1)
for(genus in genera.C3){
  genus.window <- fun.get.data.window(data = mat.genus.timeseries
                                     , age = age
                                     , clusters = clusters.tmp
                                     , clust.num = 1
                                     , window = 0.5
                                     , sequence = ss
                                     , genus = genus)
  maxima <- max(genus.window[1, ] + genus.window[2, ], na.rm= TRUE)

  plot(ss, genus.window[1, ], type = "l"
       , ylab = "Mean Relative Abundance", xlab = "Age(years)"
       , col = "red", main = genus, ylim = c(0, maxima + maxima/100))

  for(kk in 2:length(ss))
            polygon(c(ss[kk-1], ss[kk-1], ss[kk], ss[kk])
```

```
                                     , c(genus.window[1, kk-1] - genus.window[2, kk-1]
                                         , genus.window[1, kk-1] + genus.window[2, kk-1]
                                         , genus.window[1, kk-1] + genus.window[2, kk]
                                       , genus.window[1, kk-1] - genus.window[2, kk])
                                     , col = rgb(1, 0, 0, alpha = 0.2), border = NA)
}
```

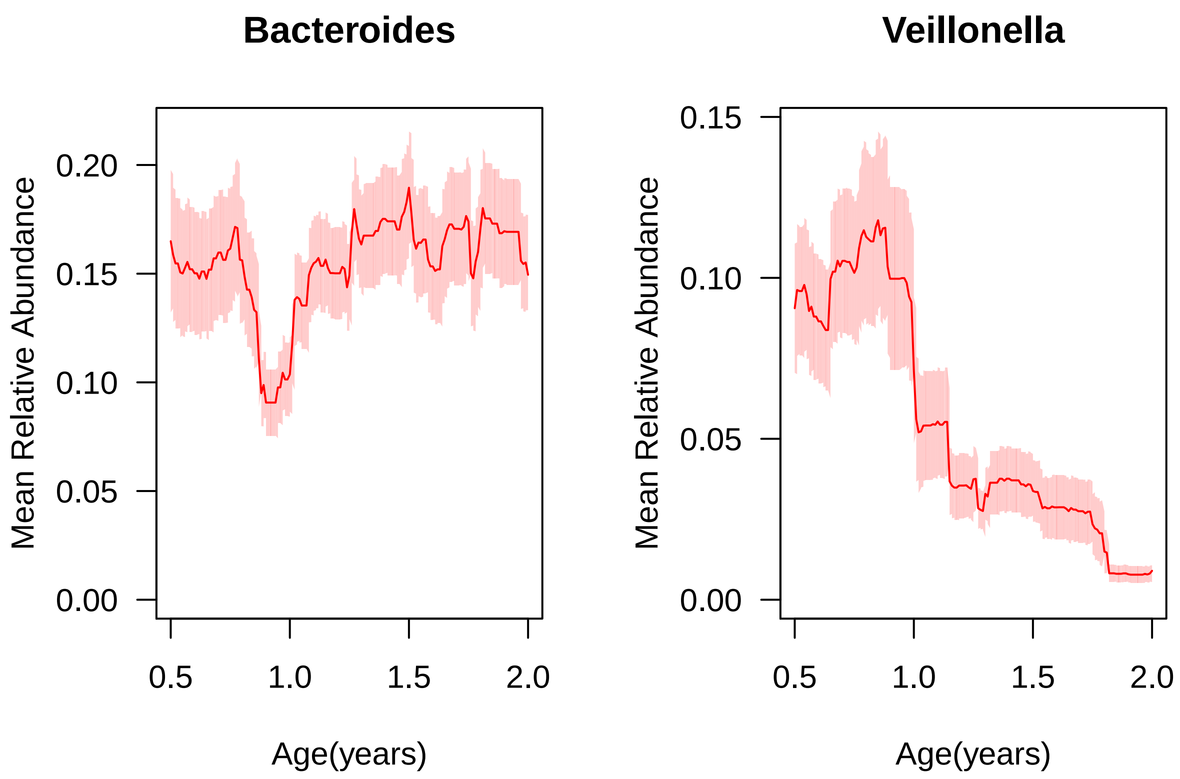

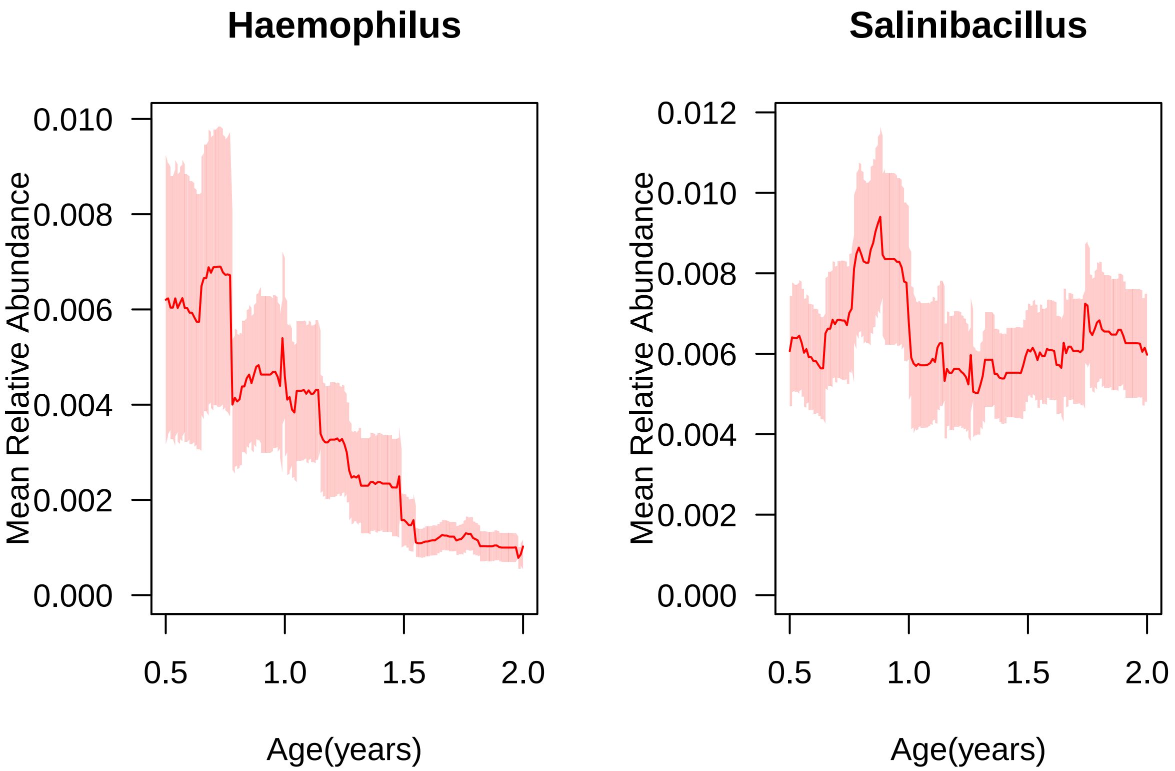

Haemophilus
Salinibacillus
Mean Relative Abundance
0.000
0.002
0.004
0.006
0.008
0.010
0.012
0.5
1.0
1.5
2.0
Age(years)

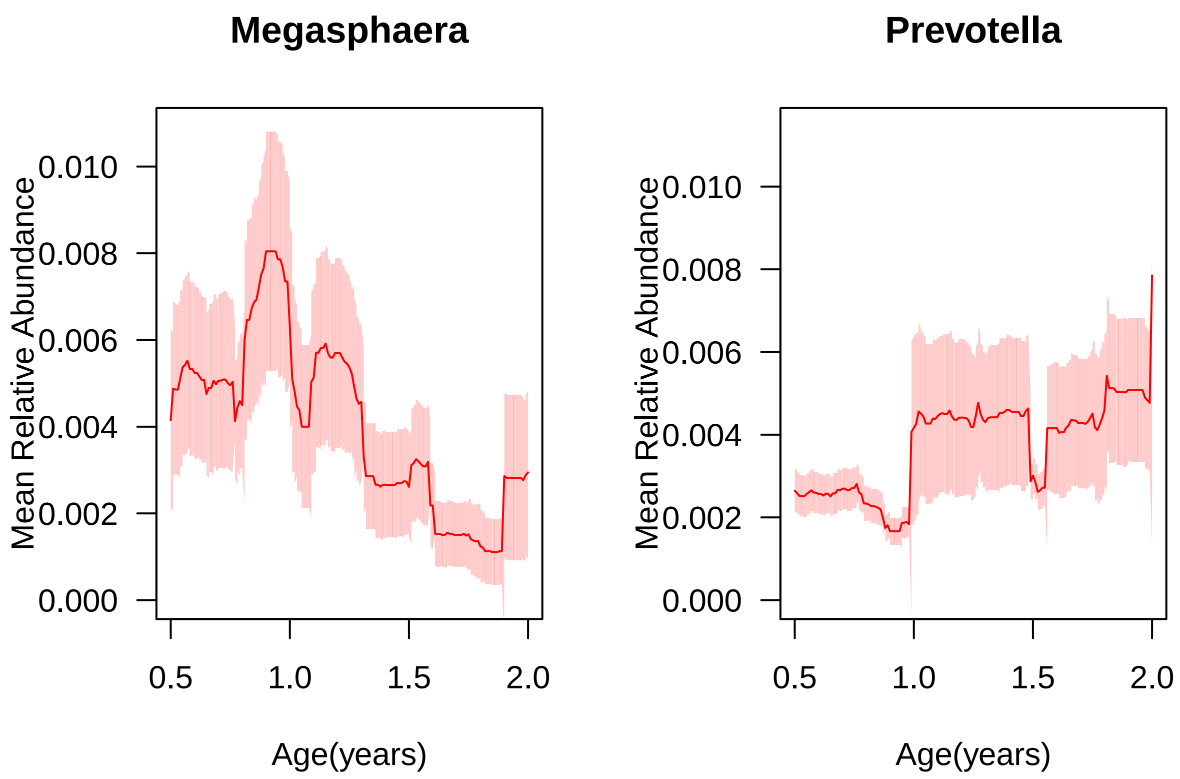

Cluster children with UniFrac distance based on abundances in communities C1, C2 and C3

In this section, the Greengenes 13.8 tree is used to estimate UniFrac distances on genus level for each community separately. For each community, a stratification of children is obtained based on abundances of genera in each community. Clustering is performed with the pam method in the R package cluster and the number of clusters is determined with the Calinski-Harabasz method from the R package fpc.

```
mat.genus.early.portion <- mat.genus.early.portion[, tree.genus$tip.label]
communities.schaub <- communities.schaub[tree.genus$tip.label, ]
list.clusters.communities <- vector(mode = "list", length = 3)
list.genus.communities <- vector(mode = "list", length = 3)
list.unifrac.communities <- vector(mode = "list", length = 3)

for(kk in unique(communities.schaub[communities.schaub != -1])){
  tree.genus.community <- drop.tip(tree.genus
                                , tree.genus$tip.label[!(tree.genus$tip.label %in%
                                  names(communities.schaub)[communities.schaub == kk])])
  mat.genus.community <- mat.genus.early.portion[, communities.schaub == kk]
  unifrac.genus.community <- unifrac2(mat.genus.community, tree.genus.community)
  num.clust <- pamk(as.dist(unifrac.genus.community), krange=1:6, criterion = "ch")$nc
  pam.community <- pam(as.dist(unifrac.genus.community), k = num.clust)$cluster
  list.clusters.communities[[kk + 1]] <- pam.community
  list.genus.communities[[kk + 1]] <- mat.genus.community
  list.unifrac.communities[[kk + 1]] <- unifrac.genus.community
```

```
}
names(list.clusters.communities) <- c("C2", "C3", "C1")
names(list.genus.communities) <- c("C2", "C3", "C1")
names(list.unifrac.communities) <- c("C2", "C3", "C1")
```

Figure 2

Here, the clustering of children based on abundances in community C3 is plotted for the first two principal coordinates obtained from PCoA analysis (Figure 2A).

```
cluster.C3 <- list.clusters.communities[["C3"]]
genus.pcoa.community <- cmdscale(list.unifrac.communities[["C3"]]
                                 , eig = TRUE, add = TRUE)
x <- genus.pcoa.community$points[, 1]
y <- genus.pcoa.community$points[, 2]
perc.var <- eigenvals(genus.pcoa.community)/sum(eigenvals(genus.pcoa.community))
par(las = 1)
plot(x, y, xlab = paste("1st principal coordinate ("
                        , round(perc.var[1]*100) , "%)",sep = "")
              , ylab = paste("2nd principal coordinate ("
                             , round(perc.var[2]*100) , "%)",sep = "")
        , ylim = c(-0.9, 0.6), xlim = c(-1, 0.7), cex = 1.5, pch = 16
        , main = "Figure 2A")
ordihull(genus.pcoa.community, cluster.C3, col="red")
abline(h=0)
abline(v=0)
```

Figure 2A

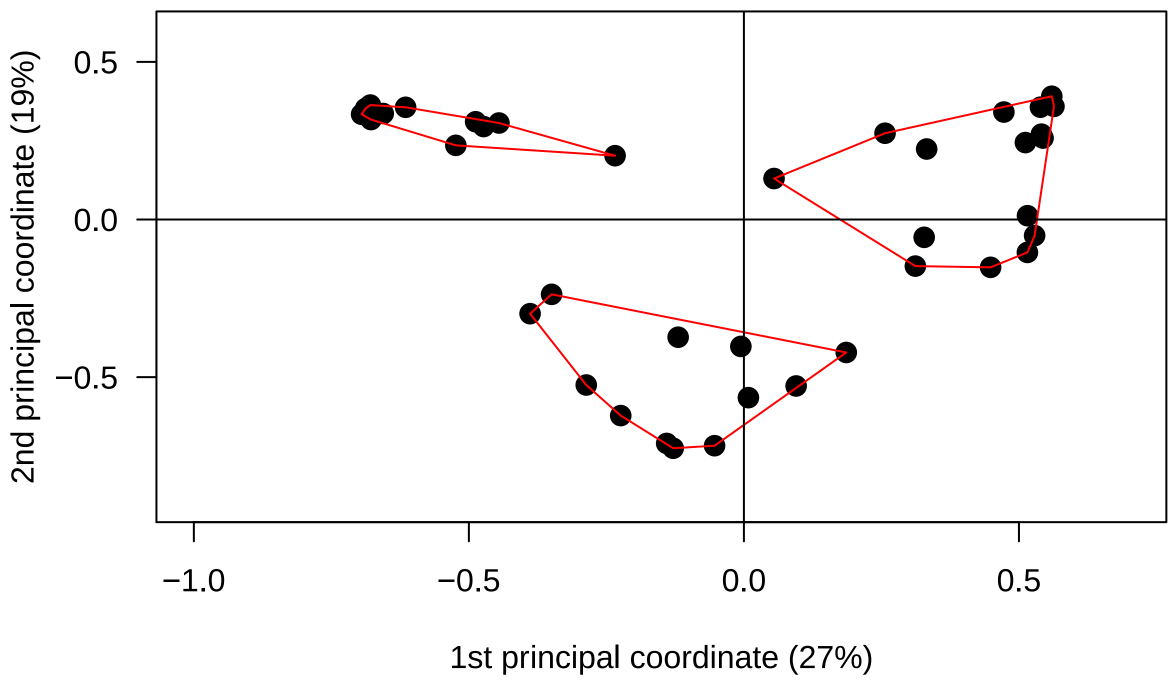

The metadata table is used for barplots of the percentage of breast/formula fed and case/control children based on the clustering in community C3 (Figure 2B).

```
breast.formula <- rep(NA, nrow(metadata.early))
names(breast.formula) <- rownames(metadata.early)
breast.formula[which(metadata.early[, "Breast feeding(any)"] == 1 &
                       metadata.early[, "formula"] == 1)] <-  "BF&Formula"
breast.formula[which(metadata.early[, "Breast feeding(any)"] == 1 &
                       metadata.early[, "formula"] == 0)] <-  "BF&noFormula"
breast.formula[which(metadata.early[, "Breast feeding(any)"] == 0 &
                       metadata.early[, "formula"] == 1)] <-  "noBF&Formula"

tab.case.control.C3 <- table(cluster.C3
                             , metadata.early[names(cluster.C3) , "Case/Control"])
tab.case.control.C3 <- tab.case.control.C3[c(3, 2, 1), ]
perc.case.control.C3 <- sweep(tab.case.control.C3, MARGIN = 1
                              , FUN = "/", rowSums(tab.case.control.C3))
tab.breast.formula.C3 <- cbind(table(cluster.C3,
                                     breast.formula[names(cluster.C3)]) ,
                               table(cluster.C3,
                                     is.na(breast.formula[names(cluster.C3)]))[, 2])
tab.breast.formula.C3 <- tab.breast.formula.C3[c(3, 2, 1), ]
perc.breast.formula.C3 <- sweep(tab.breast.formula.C3, MARGIN = 1
                                , FUN = "/", rowSums(tab.breast.formula.C3))
perc.breast.formula.C3 <- perc.breast.formula.C3[, c(2, 1, 3, 4)]
```

```
perc.combined.C3 <- matrix(0, nr = 6, nc = 6)
perc.combined.C3[1:3, 1:2] <- perc.case.control.C3
perc.combined.C3[4:6, 3:6] <- perc.breast.formula.C3
par(las = 1)
b <- barplot(100*t(perc.combined.C3), main = "Figure 2B"
             , col = c("black", "red", "blue", "lightblue", "red", "gray")
             , ylim = c(0, 130), space = c(rep(0.2, 3), 1, rep(0.2, 2))
             , yaxt = "n", ylab = "Percentage in Cluster", border = NA
                 , names.arg = rep(c("G31", "G32", "G33"), 2))
axis(2, seq(0, 100, 20), seq(0, 100, 20), las = 2)

legend(b[2], 100, c("Case", "Control"), fill = c("red", "black")
        , xjust = 0.5, yjust = 0, bty = "n", horiz = TRUE)
legend(b[5], 100, c("+/-", "+/+", "-/+", "NA")
       , fill = c("blue", "lightblue", "red", "gray"), xjust = 0.5
       , yjust = 0, bty = "n", horiz = TRUE)
```

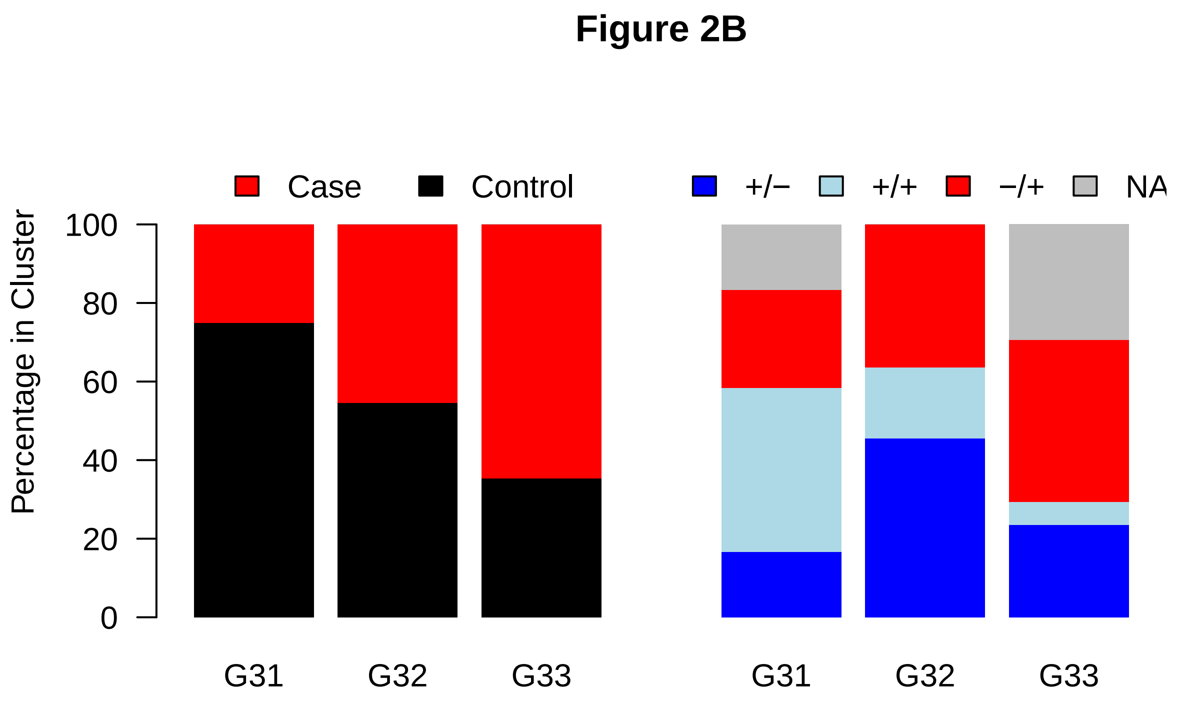

P-values of children who are still breast fed vs children who are no longer breast fed at the time of sampling are calculated between cluster G33 vs clusters G31 and G32 based on two-sided Fisher's exact test.

```
tab.breast.C3 <- table(cluster.C3
                       , metadata.early[names(cluster.C3)
                                  , "Breast feeding(any)"])[c(3, 2, 1), ]
fisher.test(rbind(colSums(tab.breast.C3[1:2, ]), tab.breast.C3[3, ]))
```

```
##
```

```
##  Fisher's Exact Test for Count Data
##
## data:  rbind(colSums(tab.breast.C3[1:2, ]), tab.breast.C3[3, ])
## p-value = 0.483
## alternative hypothesis: true odds ratio is not equal to 1
## 95 percent confidence interval:
##  0.1009235 2.4718277
## sample estimates:
## odds ratio
##  0.5103078
```

P-values of case vs control children are calculated between cluster G33 vs clusters G31 and G32 based on one-sided Fisher's exact test.

```
tab.case.control.C3 <- table(cluster.C3
                             , metadata.early[names(cluster.C3)
                                        , "Case/Control"])[c(3, 2, 1), ]
fisher.test(rbind(tab.case.control.C3[1, ], tab.case.control.C3[3, ])
            , alternative = "greater")
```

```
##
##  Fisher's Exact Test for Count Data
##
## data:  rbind(tab.case.control.C3[1, ], tab.case.control.C3[3, ])
## p-value = 0.04075
## alternative hypothesis: true odds ratio is greater than 1
## 95 percent confidence interval:
##  1.073579      Inf
## sample estimates:
## odds ratio
##   5.154952
```

Food complexity is estimated by adding the number of food components (formula, potato, vegetable, fruit and meat) already introduced at the time of sampling. Food complexity is then plotted based on the clustering of community C3 (Figure 2C).

```
food.complexity <- rowSums(metadata.early[
                      , c("formula", "potato", "vegetable", "fruit",  "meat")])
tab.food.C3 <- table(cluster.C3, food.complexity[names(cluster.C3)])
tab.food.C3 <- tab.food.C3[c(3, 2, 1), ]
perc.food.C3 <- sweep(tab.food.C3, MARGIN = 1, FUN = "/", rowSums(tab.food.C3))

par(las = 1)
barplot(100*t(perc.food.C3), beside = TRUE, main = "Figure 2C"
        , col = c("white", rgb(1, 0, 0, alpha = c(0.1, 0.2, 0.4, 0.6, 1)))
              , ylab = "Percentage in Cluster", names = c("G31", "G32", "G33"))

legend("topleft", as.character(0:5), title = "Number food components"
        , bty = "n", horiz = TRUE
                , fill = c("white", rgb(1, 0, 0, alpha = c(0.1, 0.2, 0.4, 0.6, 1))))
```

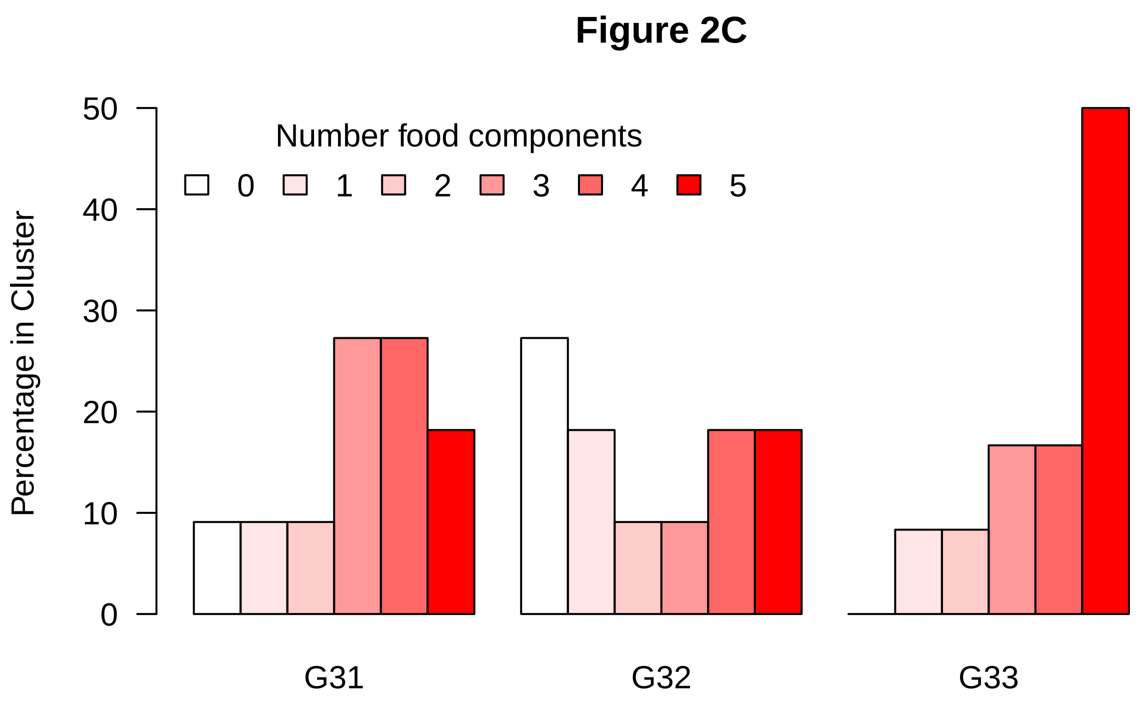

P-values of children who have >3 different food components vs children who have <= 3 food components are calculated between cluster G33 vs clusters G31 and G32 based on two-sided Fisher's exact test.

```
class.food.complexity <- food.complexity
class.food.complexity[which(food.complexity <= 3)] <- 0
class.food.complexity[which(food.complexity > 3)] <- 1
tab.food.C3 <- table(cluster.C3, class.food.complexity[names(cluster.C3)])[c(3, 2, 1), ]

fisher.test(rbind(colSums(tab.food.C3[1:2, ]), tab.food.C3[3, ]))
```

```
##
##  Fisher's Exact Test for Count Data
##
## data:  rbind(colSums(tab.food.C3[1:2, ]), tab.food.C3[3, ])
## p-value = 0.2818
## alternative hypothesis: true odds ratio is not equal to 1
## 95 percent confidence interval:
##   0.5423619 16.9012005
## sample estimates:
## odds ratio
##   2.797265
```

Here, we plot the distribution of the mean relative abundance of the top 6 abundant genera in each of the three communities based on the clustering of children from abundances of genera in community C3 (Figure 2D-2F).

```
top <- 6
mat.genus.C3 <- list.genus.communities[["C3"]]
mat.genus.C2 <- list.genus.communities[["C2"]]
mat.genus.C1 <- list.genus.communities[["C1"]]

top6 <- names(sort(colSums(mat.genus.C1), decreasing = TRUE))[1:top]
top6.genus.C1 <- mat.genus.C1[, top6]
others.genus.C1 <- mat.genus.C1[, !(colnames(mat.genus.C1) %in% top6)]
mat.genus.C1 <- cbind(top6.genus.C1, rowSums(others.genus.C1))

top6 <- names(sort(colSums(mat.genus.C2), decreasing = TRUE))[1:top]
top6.genus.C2 <- mat.genus.C2[, top6]
others.genus.C2 <- mat.genus.C2[, !(colnames(mat.genus.C2) %in% top6)]
mat.genus.C2 <- cbind(top6.genus.C2, rowSums(others.genus.C2))

top6 <- names(sort(colSums(mat.genus.C3), decreasing = TRUE))[1:top]
top6.genus.C3 <- mat.genus.C3[, top6]
others.genus.C3 <- mat.genus.C3[, !(colnames(mat.genus.C3) %in% top6)]
mat.genus.C3 <- cbind(top6.genus.C3, rowSums(others.genus.C3))

mat.genus.C1 <- mat.genus.C1[names(cluster.C3), ]
mat.genus.C2 <- mat.genus.C2[names(cluster.C3), ]
mat.genus.C3 <- mat.genus.C3[names(cluster.C3), ]

mean.clusters.genus.C3 <- rbind(colMeans(mat.genus.C3[cluster.C3 == 3, ])
                                , colMeans(mat.genus.C3[cluster.C3 == 2, ])
                                , colMeans(mat.genus.C3[cluster.C3 == 1, ]))

mean.clusters.genus.C2.by.C3 <- rbind(colMeans(mat.genus.C2[cluster.C3 == 3, ])
                                , colMeans(mat.genus.C2[cluster.C3 == 2, ])
                                , colMeans(mat.genus.C2[cluster.C3 == 1, ]))

mean.clusters.genus.C1.by.C3 <- rbind(colMeans(mat.genus.C1[cluster.C3 == 3, ])
                                , colMeans(mat.genus.C1[cluster.C3 == 2, ])
                                , colMeans(mat.genus.C1[cluster.C3 == 1, ]))

par(mfrow = c(1, 2))
barplot(t(mean.clusters.genus.C1.by.C3), las = 2, main = "Figure 2D"
        , col = c("darkblue", "royalblue4"
                  , rgb(0, 0, 1, alpha = seq(.9, .1, length = 4)), "gray")
                , ylab = "Mean relative abundance", las = 2
                , names = c("G31", "G32", "G33"))
plot.new()
legend("center", c(colnames(mean.clusters.genus.C1.by.C3)[1:top], "Other")
       , fill = c("darkblue", "royalblue4"
                  , rgb(0, 0, 1, alpha = seq(.9, .1, length = 4)), "gray"))
```

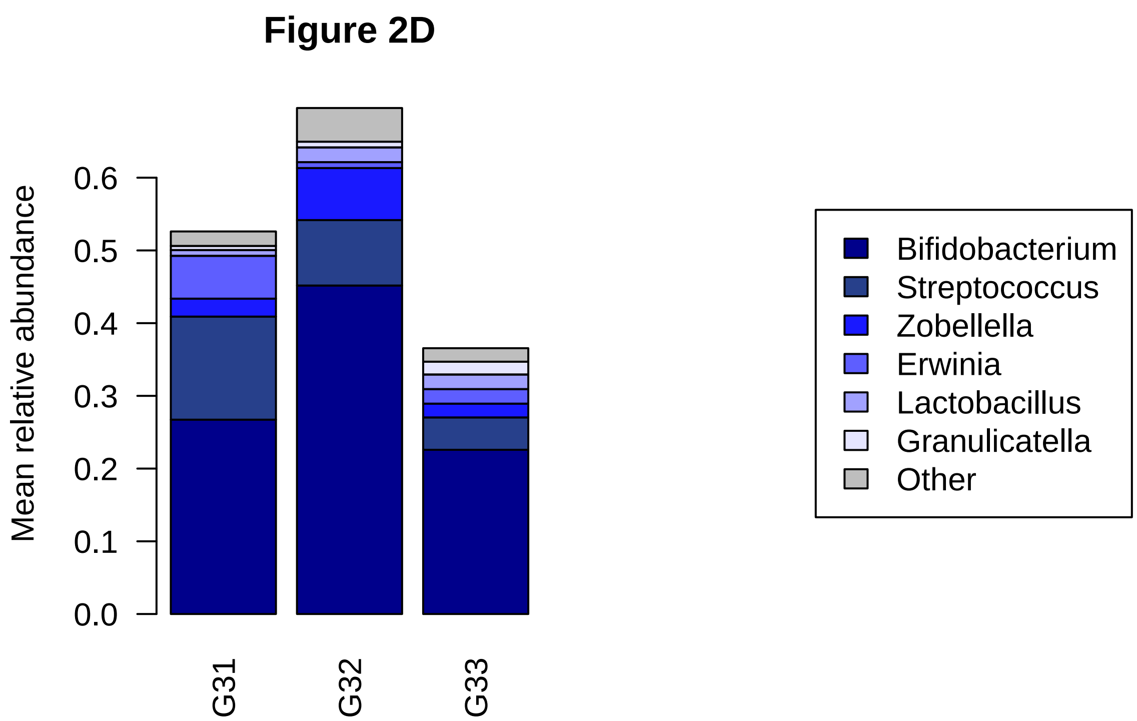


```
par(mfrow = c(1, 2))
barplot(t(mean.clusters.genus.C2.by.C3), las = 2, main = "Figure 2E"
        , col = c("darkred", "firebrick"
                  , rgb(1, 0, 0, alpha =  c(0.9, 0.6, 0.3, 0.1)) , "gray")
                , ylab = "Mean relative abundance", las = 2
                , names = c("G31", "G32", "G33"))

plot.new()
legend("center", c(colnames(mean.clusters.genus.C2.by.C3)[1:top], "Other")
           , fill = c("darkred", "firebrick"
                      , rgb(1, 0, 0, alpha =  c(0.9, 0.6, 0.3, 0.1)) , "gray"))
```

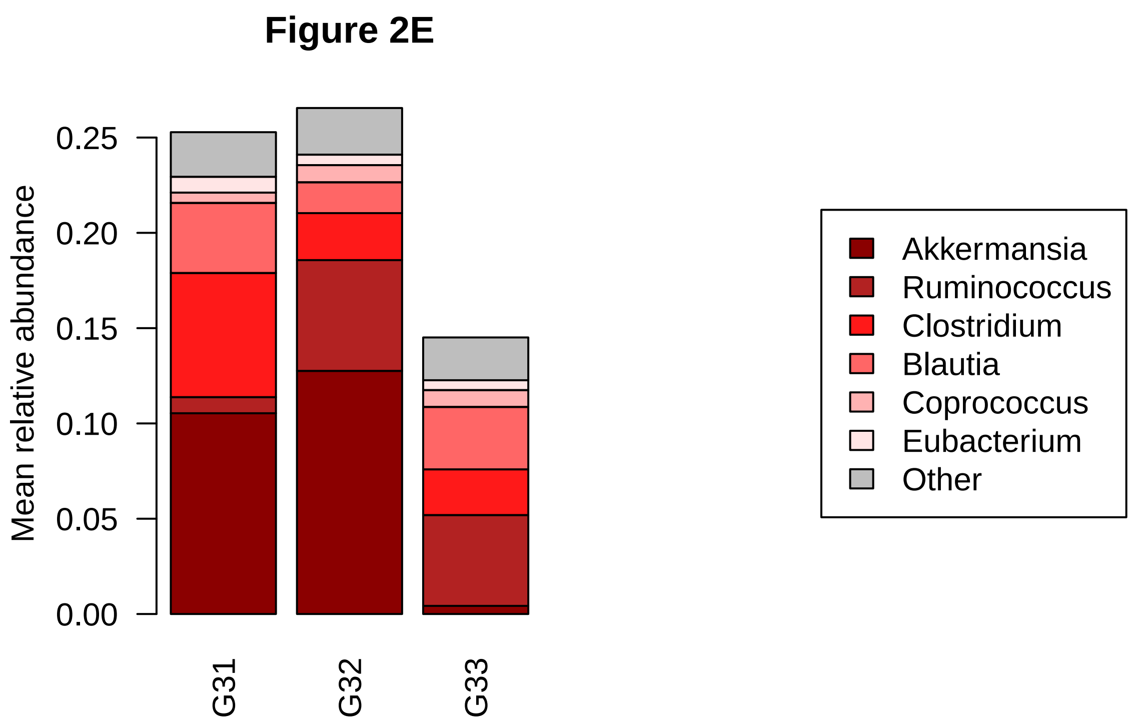


```
par(mfrow = c(1, 2))
barplot(t(mean.clusters.genus.C3), las = 2, main = "Figure 2F"
        , col = c(heat.colors(100)[seq(40, 80, lengt = 4)]
                  , "lightyellow", "white", "gray")
                , ylab = "Mean relative abundance", las = 2
                , names = c("G31", "G32", "G33"))

plot.new()
legend("center", c(colnames(mean.clusters.genus.C3)[1:top], "Other")
           , fill = c(heat.colors(100)[seq(40, 80, lengt = 4)]
                      , "lightyellow", "white", "gray"))
```

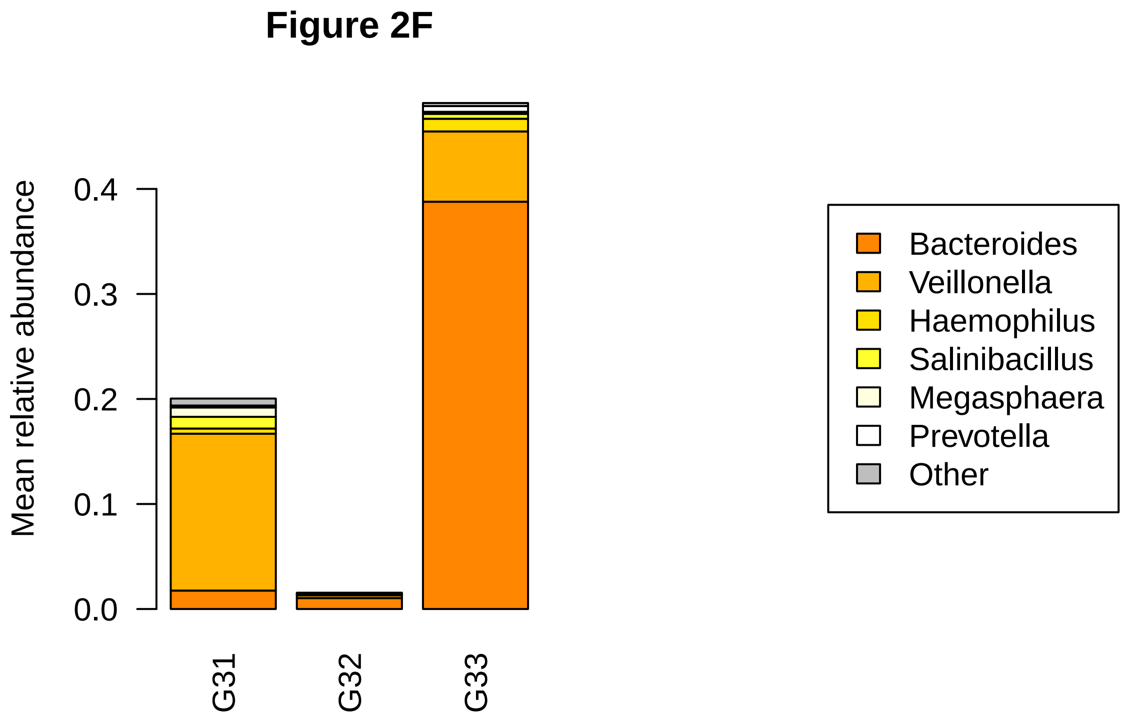

P-value Bacteroides vs meat introduction

Wilcoxon-Mann-Whitney test is used to calculate the p-value of Bacteroides abundances between children who were already fed meat and children who were not fed meat at the time of sampling.

```
wilcox.test(mat.genus.early.portion[, "Bacteroides"] ~
              metadata.early[rownames(mat.genus.early.portion), "meat"])
```

```
##
##  Wilcoxon rank sum test
##
## data:  mat.genus.early.portion[, "Bacteroides"] by metadata.early[rownames(mat.genus.early.portion),
## W = 89, p-value = 0.007073
## alternative hypothesis: true location shift is not equal to 0
```

Supplementary Figure S3

Here, the clustering of children based on abundances in community C1 is plotted for the first two principal coordinates obtained from PCoA analysis (Figure S3A).

```
cluster.C1 <- list.clusters.communities[["C1"]]
genus.pcoa.community <- cmdscale(list.unifrac.communities[["C1"]]
                                 , eig = TRUE, add = TRUE)
```

```
x <- genus.pcoa.community$points[, 1]
y <- genus.pcoa.community$points[, 2]
perc.var <- eigenvals(genus.pcoa.community)/sum(eigenvals(genus.pcoa.community))
par(las = 1)
plot(x, y, xlab = paste("1st principal coordinate ("
                        , round(perc.var[1]*100) , "%)",sep = "")
              , ylab = paste("2nd principal coordinate ("
                             , round(perc.var[2]*100) , "%)",sep = "")
        , ylim = c(-0.7, 0.7), xlim = c(-0.7, 0.6), cex = 1.5, pch = 16
        , main = "Figure S3A")
ordihull(genus.pcoa.community, cluster.C1, col="red")
abline(h=0)
abline(v=0)
```

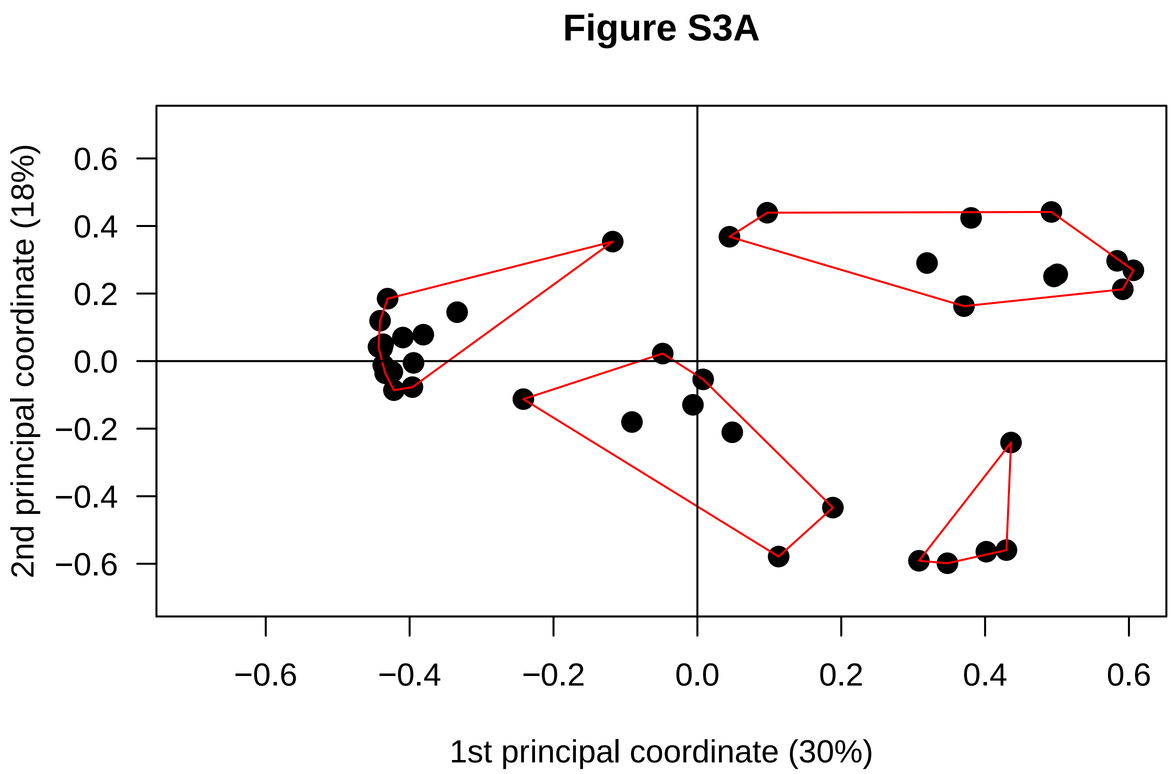

The metadata table is used for barplots of the percentage of breast/formula fed and case/control children based on the clustering in community C1 (Figure S3B).

```
tab.case.control.C1 <- table(cluster.C1
                             , metadata.early[names(cluster.C1) , "Case/Control"])
tab.case.control.C1 <- tab.case.control.C1[c(2, 3, 4, 1), ]
perc.case.control.C1 <- sweep(tab.case.control.C1, MARGIN = 1
                              , FUN = "/", rowSums(tab.case.control.C1))
tab.breast.formula.C1 <- cbind(table(cluster.C1,
                                     breast.formula[names(cluster.C1)]) ,
                               table(cluster.C1,
                                     is.na(breast.formula[names(cluster.C1)]))[, 2])
```

```r
tab.breast.formula.C1 <- tab.breast.formula.C1[c(2, 3, 4, 1), ]
perc.breast.formula.C1 <- sweep(tab.breast.formula.C1, MARGIN = 1
                                , FUN = "/", rowSums(tab.breast.formula.C1))
perc.breast.formula.C1 <- perc.breast.formula.C1[, c(2, 1, 3, 4)]

perc.combined.C1 <- matrix(0, nr = 8, nc = 6)
perc.combined.C1[1:4, 1:2] <- perc.case.control.C1
perc.combined.C1[5:8, 3:6] <- perc.breast.formula.C1
par(las = 1)
b <- barplot(100*t(perc.combined.C1)
             , col = c("black", "red", "blue", "lightblue", "red", "gray")
             , ylim = c(0, 130), space = c(rep(0.2, 4), 1, rep(0.2, 3))
             , yaxt = "n", ylab = "Percentage in Cluster", border = NA
                 , names.arg = rep(c("G11", "G12", "G13", "G14"), 2)
                 , main = "Figure S3B")
axis(2, seq(0, 100, 20), seq(0, 100, 20), las = 2)

legend(mean(b[2:3]), 100, c("Case", "Control"), fill = c("red", "black")
        , xjust = 0.5, yjust = 0, bty = "n", horiz = TRUE)
legend(mean(b[6:7]), 100, c("+/-", "+/+", "-/+", "NA")
       , fill = c("blue", "lightblue", "red", "gray"), xjust = 0.5
       , yjust = 0, bty = "n", horiz = TRUE)
```

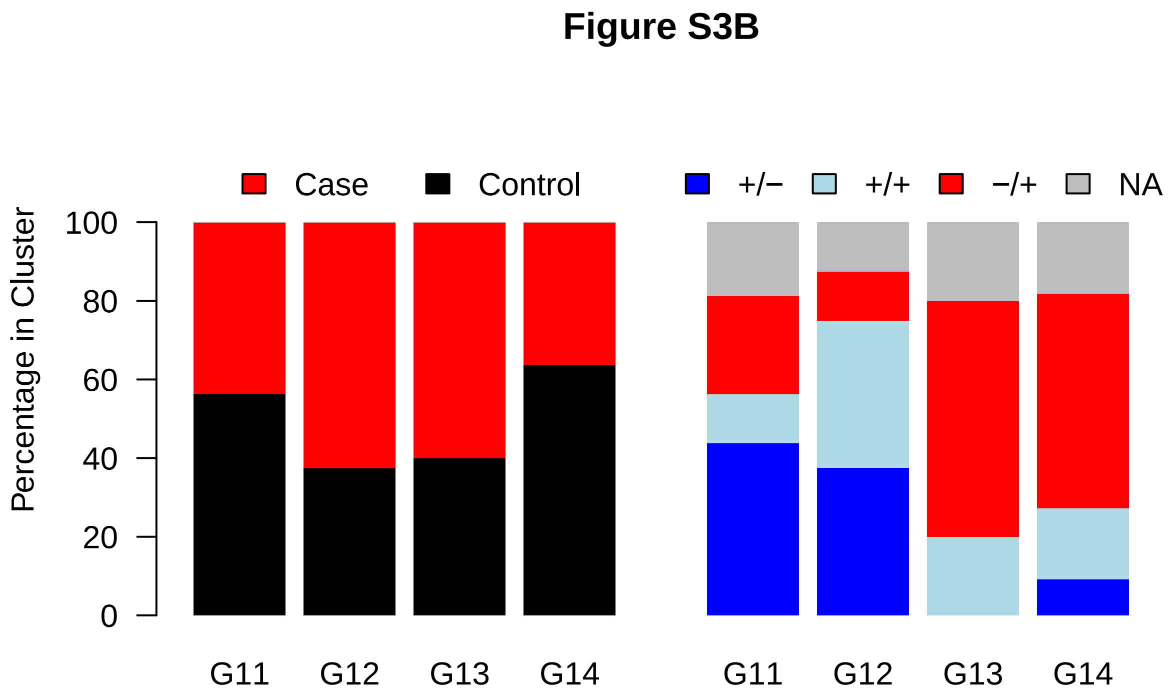

P-values of children who are still breast fed vs children who are no longer breast fed at the time of sampling are calculated between clusters G11 and G12 vs clusters G13 and G14 based on two-sided Fisher's exact test.

```
tab.breast.C1 <- table(cluster.C1
                       , metadata.early[names(cluster.C1)
                                  , "Breast feeding(any)"])[c(2, 3, 4, 1), ]
fisher.test(rbind(colSums(tab.breast.C1[1:2, ]), colSums(tab.breast.C1[3:4, ])))
```

```
##
##  Fisher's Exact Test for Count Data
##
## data:
## p-value = 0.012
## alternative hypothesis: true odds ratio is not equal to 1
## 95 percent confidence interval:
##  0.02088598 0.75729716
## sample estimates:
## odds ratio
##  0.1402171
```

Food complexity is plotted based on the clustering of community C1 (Figure S3C).

```
tab.food.C1 <- table(cluster.C1, food.complexity[names(cluster.C1)])
tab.food.C1 <- tab.food.C1[c(2, 3, 4, 1), ]
perc.food.C1 <- sweep(tab.food.C1, MARGIN = 1, FUN = "/", rowSums(tab.food.C1))

par(las = 1)
barplot(100*t(perc.food.C1), beside = TRUE
        , col = c("white", rgb(1, 0, 0, alpha = c(0.1, 0.2, 0.4, 0.6, 1)))
              , ylab = "Percentage in Cluster", names = c("G11", "G12", "G13", "G14")
              , main = "Figure S3C")

legend("topleft", as.character(0:5), title = "Number food components"
        , bty = "n", horiz = TRUE
                , fill = c("white", rgb(1, 0, 0, alpha = c(0.1, 0.2, 0.4, 0.6, 1))))
```

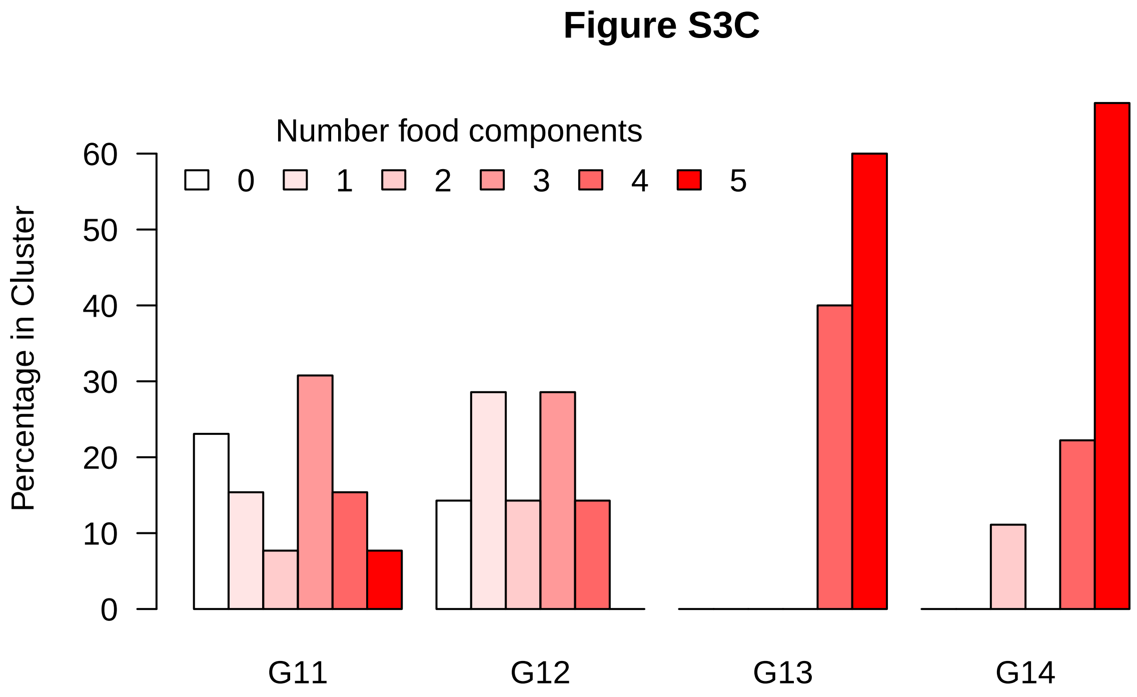

P-values of children who have >3 different food components vs children who have <= 3 food components are calculated between clusters G11 and G12 vs clusters G13 and G14 based on two-sided Fisher's exact test.

```
tab.food.C1 <- table(cluster.C1, class.food.complexity[names(cluster.C1)])[c(2, 3, 4, 1), ]

fisher.test(rbind(colSums(tab.food.C1[1:2, ]), colSums(tab.food.C1[3:4, ])))
```

```
##
##  Fisher's Exact Test for Count Data
##
## data:  rbind(colSums(tab.food.C1[1:2, ]), colSums(tab.food.C1[3:4, ]))
## p-value = 5.911e-05
## alternative hypothesis: true odds ratio is not equal to 1
## 95 percent confidence interval:
##     4.497542 2301.519392
## sample estimates:
## odds ratio
##   43.80884
```

Here, we plot the distribution of the mean relative abundance of the top 6 abundant genera in each of the three communities based on the clustering of children from abundances of genera in community C1 (Figure S3D-S3F).

```
mat.genus.C1 <- mat.genus.C1[names(cluster.C1), ]
mat.genus.C2 <- mat.genus.C2[names(cluster.C1), ]
```

```
mat.genus.C3 <- mat.genus.C3[names(cluster.C1), ]

mean.clusters.genus.C1 <- rbind(colMeans(mat.genus.C1[cluster.C1 == 2, ])
                                , colMeans(mat.genus.C1[cluster.C1 == 3, ])
                                , colMeans(mat.genus.C1[cluster.C1 == 4, ])
                                , colMeans(mat.genus.C1[cluster.C1 == 1, ]))

mean.clusters.genus.C2.by.C1 <- rbind(colMeans(mat.genus.C2[cluster.C1 == 2, ])
                                , colMeans(mat.genus.C2[cluster.C1 == 3, ])
                                , colMeans(mat.genus.C2[cluster.C1 == 4, ])
                                , colMeans(mat.genus.C2[cluster.C1 == 1, ]))

mean.clusters.genus.C3.by.C1 <- rbind(colMeans(mat.genus.C3[cluster.C1 == 2, ])
                                , colMeans(mat.genus.C3[cluster.C1 == 3, ])
                                , colMeans(mat.genus.C3[cluster.C1 == 4, ])
                                , colMeans(mat.genus.C3[cluster.C1 == 1, ]))

par(mfrow = c(1, 2))
barplot(t(mean.clusters.genus.C1), las = 2, main = "Figure S3D"
        , col = c("darkblue", "royalblue4"
                  , rgb(0, 0, 1, alpha = seq(.9, .1, length = 4)), "gray")
                , ylab = "Mean relative abundance", las = 2
                , names = c("G11", "G12", "G13", "G14"))
plot.new()
legend("center", c(colnames(mean.clusters.genus.C1)[1:top], "Other")
       , fill = c("darkblue", "royalblue4"
                  , rgb(0, 0, 1, alpha = seq(.9, .1, length = 4)), "gray"))
```

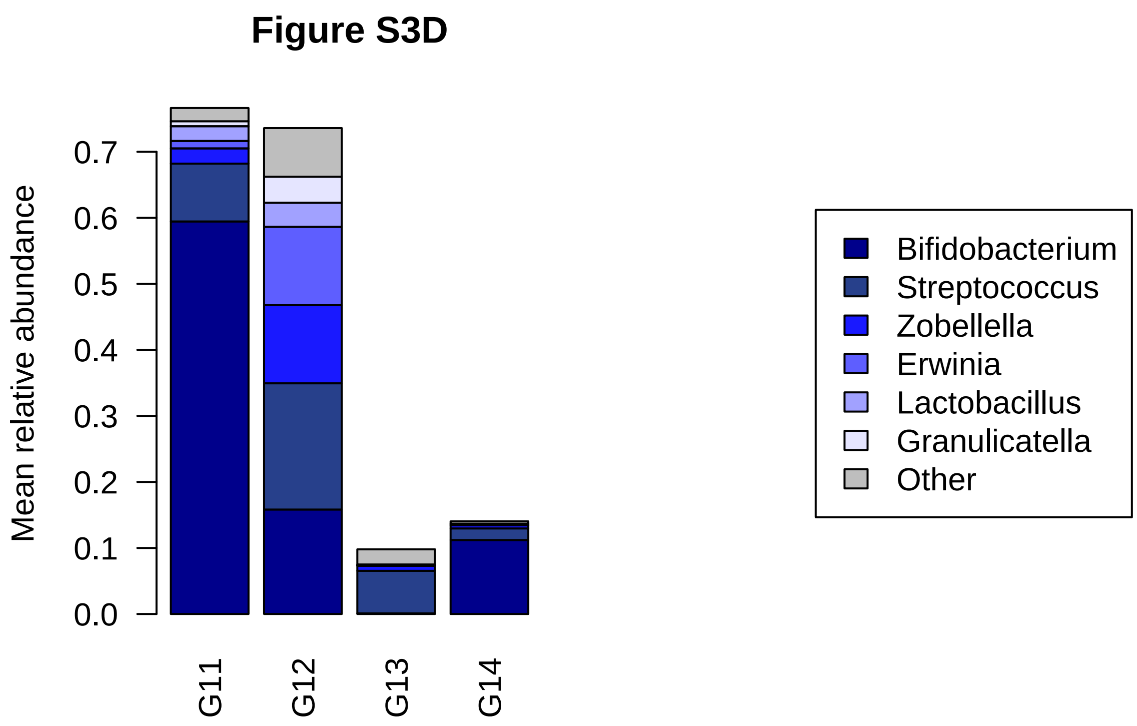


```r
par(mfrow = c(1, 2))
barplot(t(mean.clusters.genus.C2.by.C1), las = 2, main = "Figure S3E"
        , col = c("darkred", "firebrick"
                  , rgb(1, 0, 0, alpha =  c(0.9, 0.6, 0.3, 0.1)) , "gray")
                , ylab = "Mean relative abundance", las = 2
                , names = c("G11", "G12", "G13", "G14"))

plot.new()
legend("center", c(colnames(mean.clusters.genus.C2.by.C1)[1:top], "Other")
           , fill = c("darkred", "firebrick"
                      , rgb(1, 0, 0, alpha =  c(0.9, 0.6, 0.3, 0.1)) , "gray"))
```

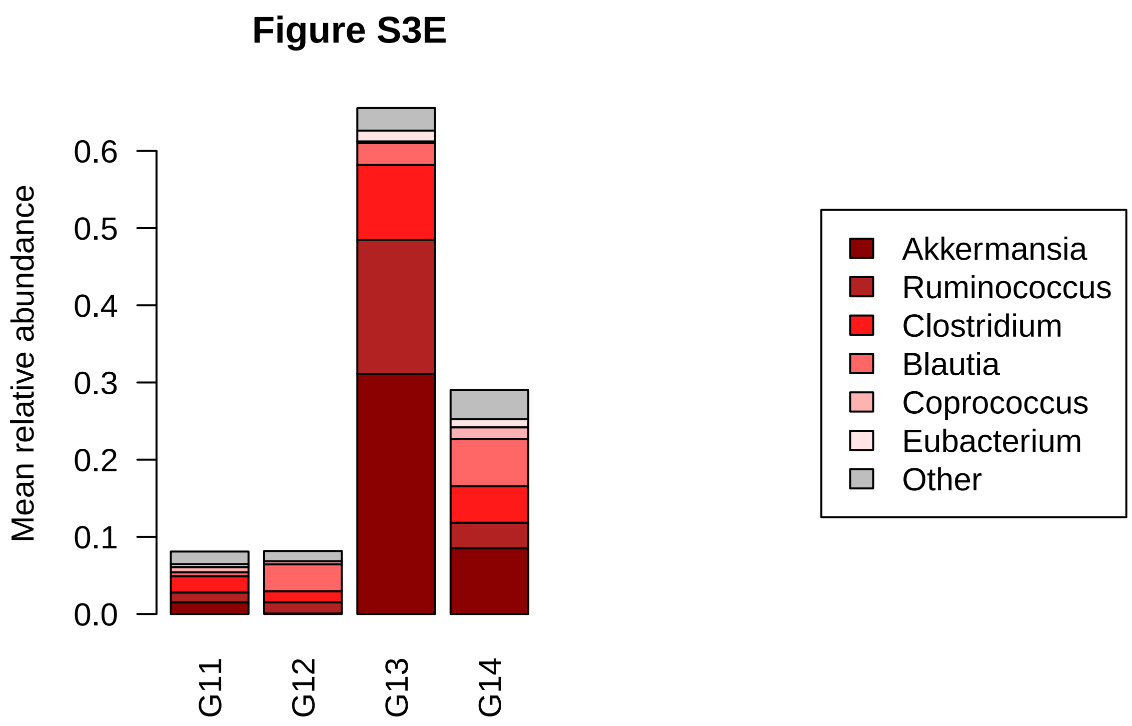


```r
par(mfrow = c(1, 2))
barplot(t(mean.clusters.genus.C3.by.C1), las = 2, main = "Figure S3F"
        , col = c(heat.colors(100)[seq(40, 80, lengt = 4)]
                  , "lightyellow", "white", "gray")
                , ylab = "Mean relative abundance", las = 2
                , names = c("G11", "G12", "G13", "G14"))

plot.new()
legend("center", c(colnames(mean.clusters.genus.C3.by.C1)[1:top], "Other")
           , fill = c(heat.colors(100)[seq(40, 80, lengt = 4)]
                      , "lightyellow", "white", "gray"))
```

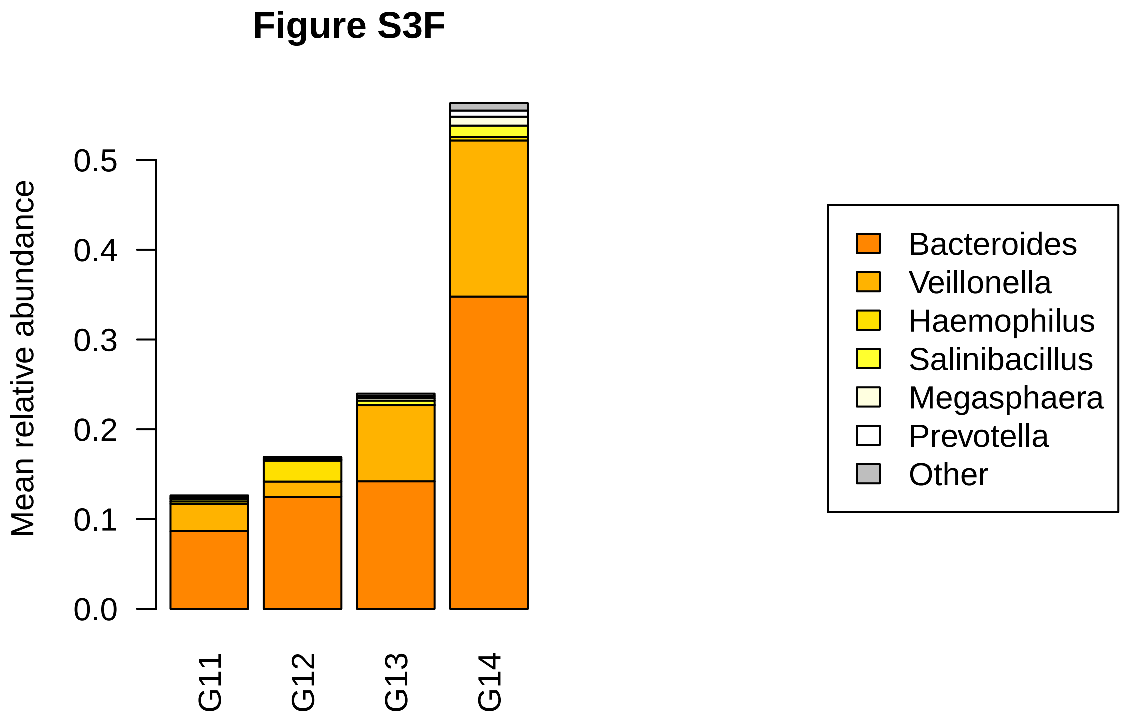

Supplementary Figure S4

Here, the clustering of children based on abundances in community C2 is plotted for the first two principal coordinates obtained from PCoA analysis (Figure S4A).

```
cluster.C2 <- list.clusters.communities[["C2"]]
genus.pcoa.community <- cmdscale(list.unifrac.communities[["C2"]]
                                  , eig = TRUE, add = TRUE)
x <- genus.pcoa.community$points[, 1]
y <- genus.pcoa.community$points[, 2]
perc.var <- eigenvals(genus.pcoa.community)/sum(eigenvals(genus.pcoa.community))
par(las = 1)
plot(x, y, xlab = paste("1st principal coordinate ("
                        , round(perc.var[1]*100) , "%)",sep = "")
              , ylab = paste("2nd principal coordinate ("
                             , round(perc.var[2]*100) , "%)",sep = "")
        , ylim = c(-0.4, 0.7), xlim = c(-0.9, 0.6), cex = 1.5, pch = 16
        , main = "Figure S4A")
ordihull(genus.pcoa.community, cluster.C2, col="red")
abline(h=0)
abline(v=0)
```

Figure S4A

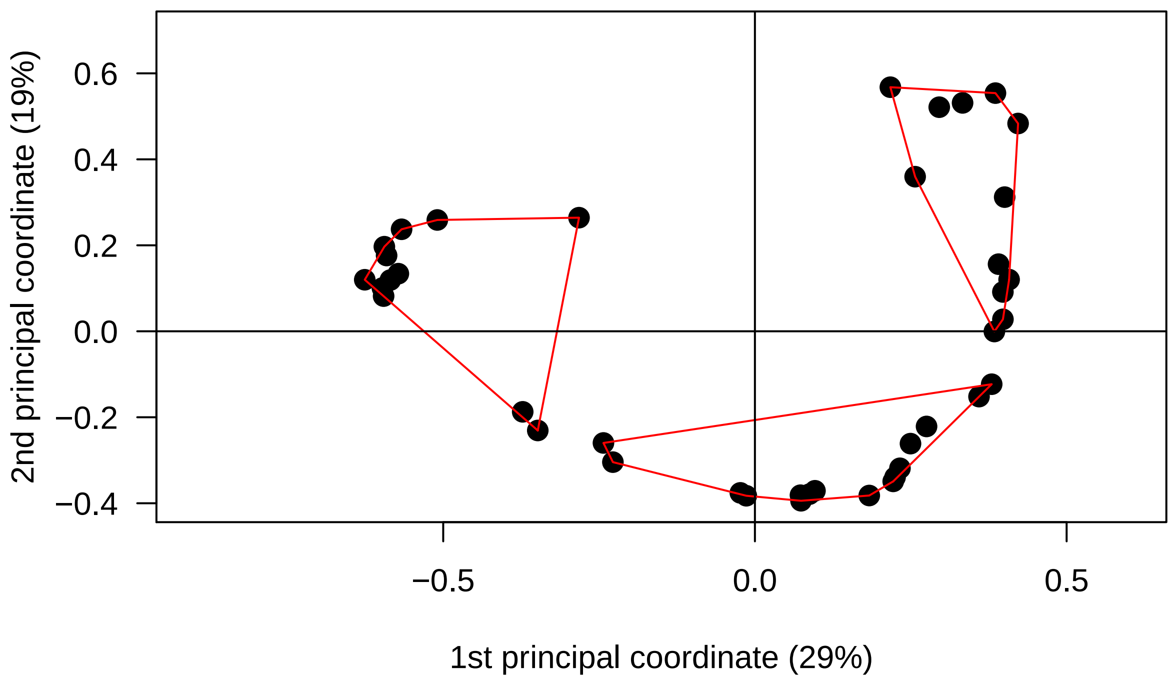

The metadata table is used for barplots of the percentage of breast/formula fed and case/control children based on the clustering in community C2 (Figure S4B).

```
tab.case.control.C2 <- table(cluster.C2
                             , metadata.early[names(cluster.C2) , "Case/Control"])
tab.case.control.C2 <- tab.case.control.C2[c(2, 3, 1), ]
perc.case.control.C2 <- sweep(tab.case.control.C2, MARGIN = 1
                              , FUN = "/", rowSums(tab.case.control.C2))
tab.breast.formula.C2 <- cbind(table(cluster.C2,
                                     breast.formula[names(cluster.C2)]) ,
                               table(cluster.C2,
                                     is.na(breast.formula[names(cluster.C2)]))[, 2])
tab.breast.formula.C2 <- tab.breast.formula.C2[c(2, 3, 1), ]
perc.breast.formula.C2 <- sweep(tab.breast.formula.C2, MARGIN = 1
                                , FUN = "/", rowSums(tab.breast.formula.C2))
perc.breast.formula.C2 <- perc.breast.formula.C2[, c(2, 1, 3, 4)]

perc.combined.C2 <- matrix(0, nr = 6, nc = 6)
perc.combined.C2[1:3, 1:2] <- perc.case.control.C2
perc.combined.C2[4:6, 3:6] <- perc.breast.formula.C2
par(las = 1)
b <- barplot(100*t(perc.combined.C2)
             , col = c("black", "red", "blue", "lightblue", "red", "gray")
             , ylim = c(0, 130), space = c(rep(0.2, 3), 1, rep(0.2, 2))
             , yaxt = "n", ylab = "Percentage in Cluster", border = NA
                , names.arg = rep(c("G21", "G22", "G23"), 2)
```

```
                  , main = "Figure S4B")
axis(2, seq(0, 100, 20), seq(0, 100, 20), las = 2)

legend(b[2], 100, c("Case", "Control"), fill = c("red", "black")
        , xjust = 0.5, yjust = 0, bty = "n", horiz = TRUE)
legend(b[5], 100, c("+/-", "+/+", "-/+", "NA")
       , fill = c("blue", "lightblue", "red", "gray"), xjust = 0.5
       , yjust = 0, bty = "n", horiz = TRUE)
```

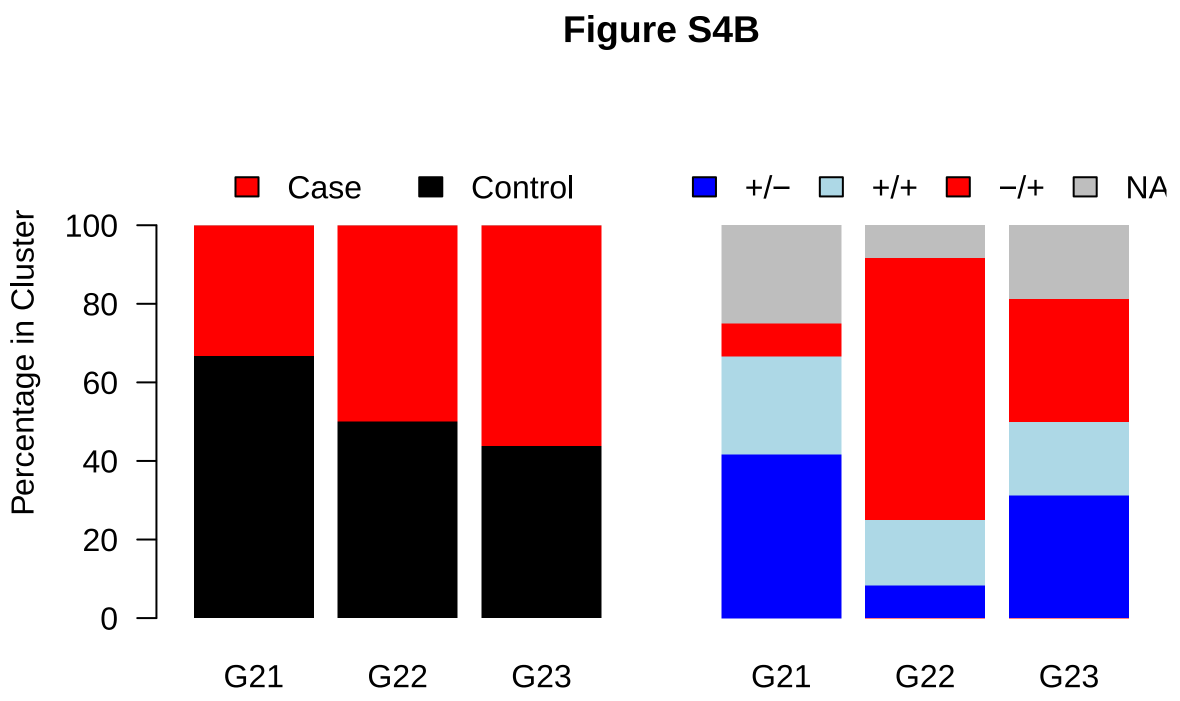

P-values of children who are still breast fed vs children who are no longer breast fed at the time of sampling are calculated between clusters G21 and G23 vs cluster G22 based on two-sided Fisher's exact test.

```
tab.breast.C2 <- table(cluster.C2
                       , metadata.early[names(cluster.C2)
                                  , "Breast feeding(any)"])[c(2, 3, 1), ]
fisher.test(rbind(colSums(tab.breast.C2[c(1, 3), ]), tab.breast.C2[2, ]))
```

```
##
##  Fisher's Exact Test for Count Data
##
## data:  rbind(colSums(tab.breast.C2[c(1, 3), ]), tab.breast.C2[2, ])
## p-value = 0.01148
## alternative hypothesis: true odds ratio is not equal to 1
## 95 percent confidence interval:
##  0.0170559 0.7826324
```

```
## sample estimates:
## odds ratio
##  0.1342398
```

Food complexity is plotted based on the clustering of community C2.

```r
tab.food.C2 <- table(cluster.C2,food.complexity[names(cluster.C2)])
tab.food.C2 <- tab.food.C2[c(2, 3, 1), ]
perc.food.C2 <- sweep(tab.food.C2, MARGIN = 1, FUN = "/", rowSums(tab.food.C2))

par(las = 1)
barplot(100*t(perc.food.C2), beside = TRUE
        , col = c("white", rgb(1, 0, 0, alpha = c(0.1, 0.2, 0.4, 0.6, 1)))
              , ylab = "Percentage in Cluster", names = c("G21", "G22", "G23")
              , main = "Figure S4C")

legend("topleft", as.character(0:5), title = "Number food components"
        , bty = "n", horiz = TRUE
                , fill = c("white", rgb(1, 0, 0, alpha = c(0.1, 0.2, 0.4, 0.6, 1))))
```

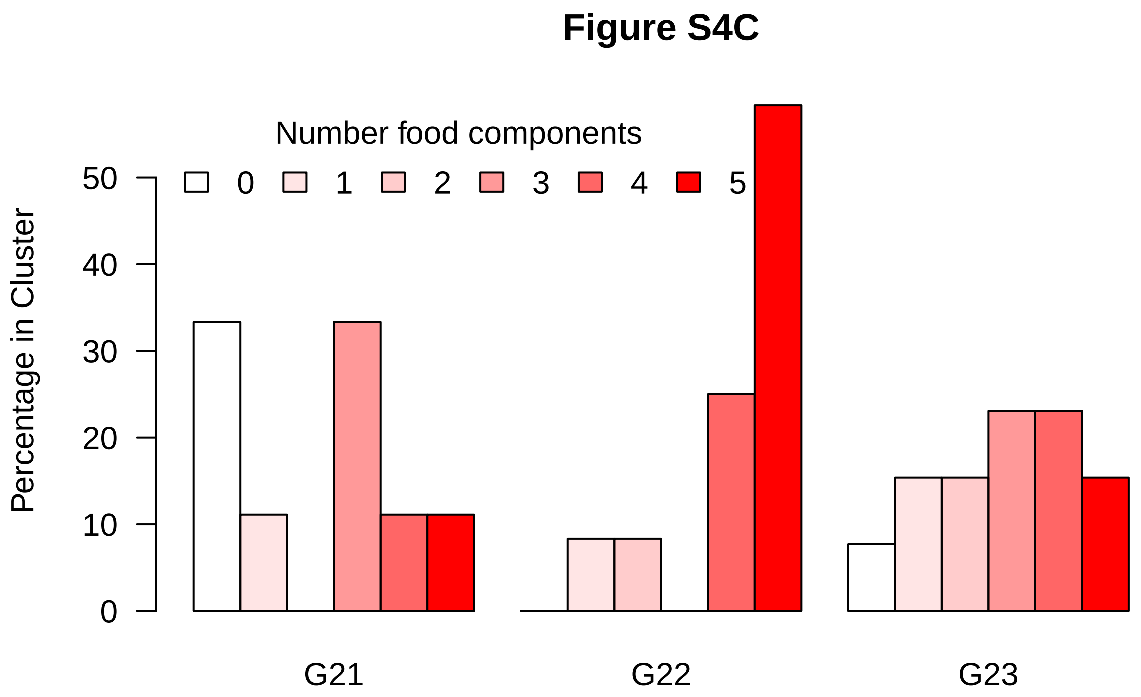

P-values of children who have >3 different food components vs children who have <= 3 food components are calculated between clusters G21 and G23 vs cluster G22 based on two-sided Fisher's exact test.

```r
tab.food.C2 <- table(cluster.C2, class.food.complexity[names(cluster.C2)])[c(2, 3, 1), ]

fisher.test(rbind(colSums(tab.food.C2[c(1, 3), ]), tab.food.C2[2, ]))
```

```
##
##  Fisher's Exact Test for Count Data
##
## data:  rbind(colSums(tab.food.C2[c(1, 3), ]), tab.food.C2[2, ])
## p-value = 0.01044
## alternative hypothesis: true odds ratio is not equal to 1
## 95 percent confidence interval:
##    1.532379 116.768730
## sample estimates:
## odds ratio
##   9.899265
```

Here, we plot the distribution of the mean relative abundance of the top 6 abundant genera in each of the three communities based on the clustering of children from abundances of genera in community C2 (Figure S4D-S4F).

```
mat.genus.C1 <- mat.genus.C1[names(cluster.C2), ]
mat.genus.C2 <- mat.genus.C2[names(cluster.C2), ]
mat.genus.C3 <- mat.genus.C3[names(cluster.C2), ]

mean.clusters.genus.C2 <- rbind(colMeans(mat.genus.C2[cluster.C2 == 2, ])
                                    , colMeans(mat.genus.C2[cluster.C2 == 3, ])
                                    , colMeans(mat.genus.C2[cluster.C2 == 1, ]))

mean.clusters.genus.C1.by.C2 <- rbind(colMeans(mat.genus.C1[cluster.C2 == 2, ])
                                    , colMeans(mat.genus.C1[cluster.C2 == 3, ])
                                    , colMeans(mat.genus.C1[cluster.C2 == 1, ]))

mean.clusters.genus.C3.by.C2 <- rbind(colMeans(mat.genus.C3[cluster.C2 == 2, ])
                                    , colMeans(mat.genus.C3[cluster.C2 == 3, ])
                                    , colMeans(mat.genus.C3[cluster.C2 == 1, ]))

par(mfrow = c(1, 2))
barplot(t(mean.clusters.genus.C1.by.C2), las = 2, main = "Figure S4D"
        , col = c("darkblue", "royalblue4"
                  , rgb(0, 0, 1, alpha = seq(.9, .1, length = 4)), "gray")
                , ylab = "Mean relative abundance", las = 2
                , names = c("G21", "G22", "G23"))
plot.new()
legend("center", c(colnames(mean.clusters.genus.C1.by.C2)[1:top], "Other")
       , fill = c("darkblue", "royalblue4"
                  , rgb(0, 0, 1, alpha = seq(.9, .1, length = 4)), "gray"))
```

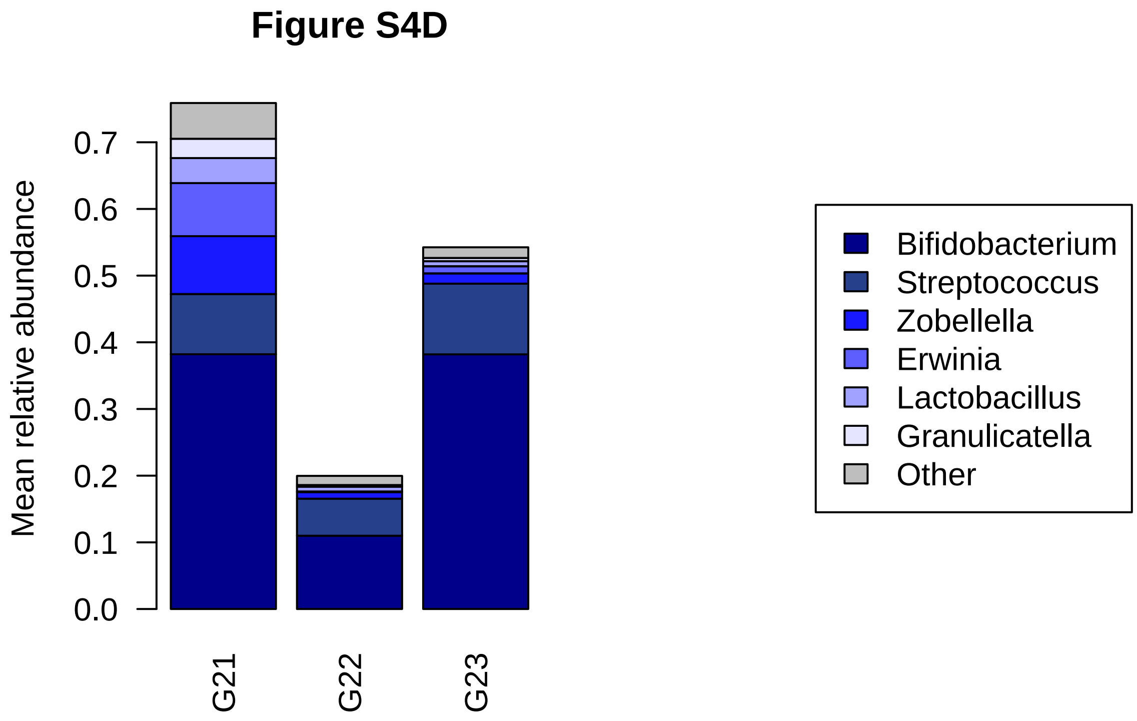


```r
par(mfrow = c(1, 2))
barplot(t(mean.clusters.genus.C2), las = 2, main = "Figure S4E"
        , col = c("darkred", "firebrick"
                  , rgb(1, 0, 0, alpha =  c(0.9, 0.6, 0.3, 0.1)) , "gray")
                , ylab = "Mean relative abundance", las = 2
                , names = c("G21", "G22", "G23"))

plot.new()
legend("center", c(colnames(mean.clusters.genus.C2)[1:top], "Other")
           , fill = c("darkred", "firebrick"
                      , rgb(1, 0, 0, alpha =  c(0.9, 0.6, 0.3, 0.1)) , "gray"))
```

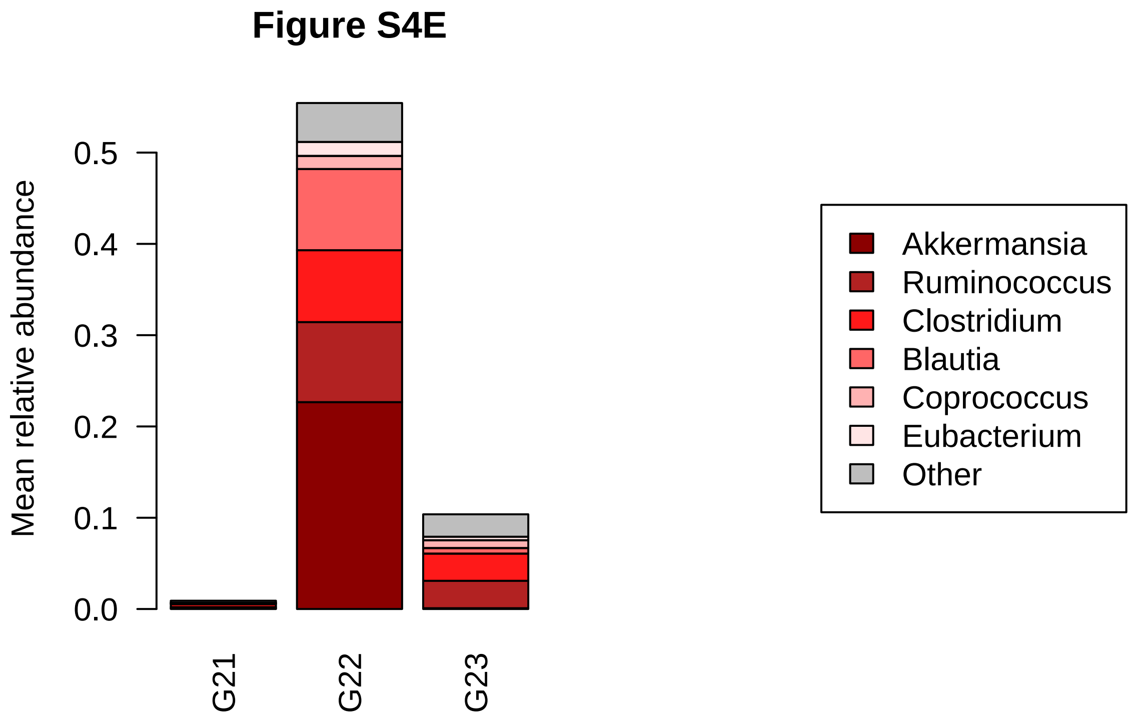


```r
par(mfrow = c(1, 2))
barplot(t(mean.clusters.genus.C3.by.C2), las = 2, main = "Figure S4F"
        , col = c(heat.colors(100)[seq(40, 80, lengt = 4)]
                  , "lightyellow", "white", "gray")
                , ylab = "Mean relative abundance", las = 2
                , names = c("G21", "G22", "G23"))

plot.new()
legend("center", c(colnames(mean.clusters.genus.C3.by.C2)[1:top], "Other")
           , fill = c(heat.colors(100)[seq(40, 80, lengt = 4)]
                      , "lightyellow", "white", "gray"))
```

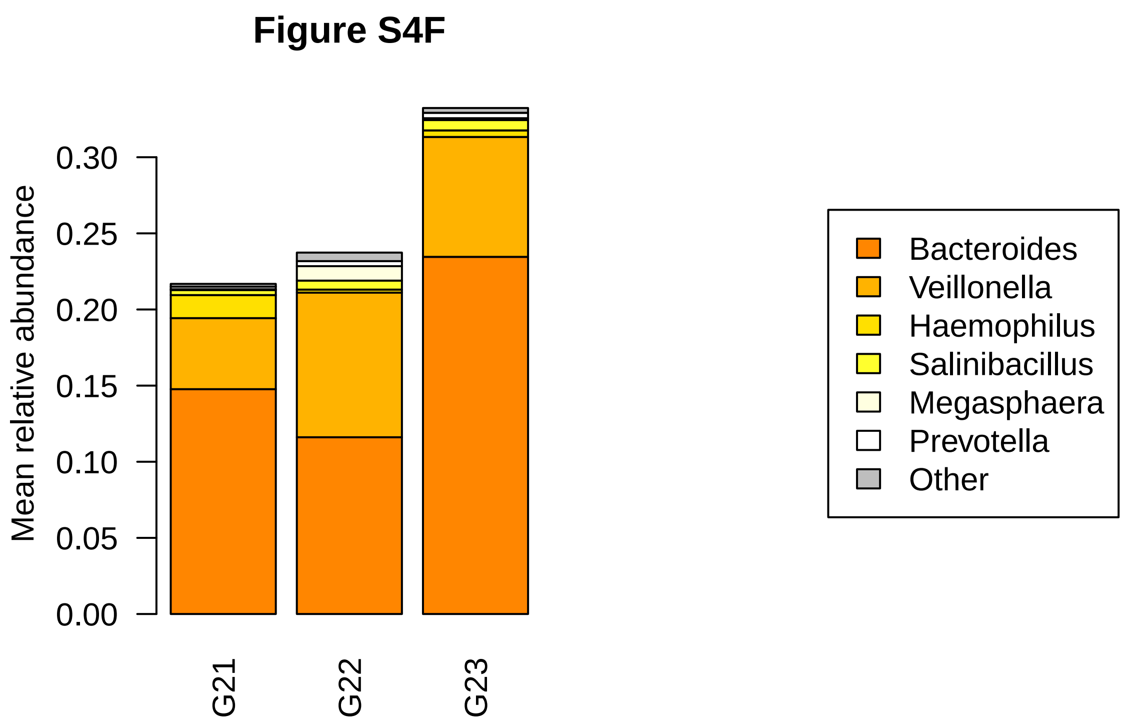

Figure 3

In this section, Kaplan-Meier curves and corresponding log-rank p-values are computed between subgroups G31 and G32 vs subgroup G33. The age of seroconversion is used as event time (Figure 3A).

```
seroconversion <- rep(1, nrow(metadata.early))
seroconversion[is.na(metadata.early[, "seroconversion_age"])] <- 0
time.seroconversion <- as.numeric(metadata.early[, "seroconversion_age"])
time.seroconversion[is.na(metadata.early[, "seroconversion_age"])] <-
  as.numeric(metadata.early[is.na(metadata.early[, "seroconversion_age"]), "max_sample_age"])
metadata.early.seroconversion <- cbind(seroconversion, time.seroconversion)
rownames(metadata.early.seroconversion) <- rownames(metadata.early)
metadata.early.seroconversion <- metadata.early.seroconversion[names(cluster.C3), ]
surv.obj <- censor(Surv(metadata.early.seroconversion[, 2]
                        , metadata.early.seroconversion[, 1]), 5)
par(las = 1)
survplot(surv.obj ~ cluster.C3 == 1, main = "Figure 3A"
         , snames = c("G31&G32", "G33"), col = c("blue", "red")
              , show.n.risk = T, stitle = "Cluster"
              , ylab = "1 - P(seroconversion)"
              , xlab = "Age (years)", lwd = 2)
```

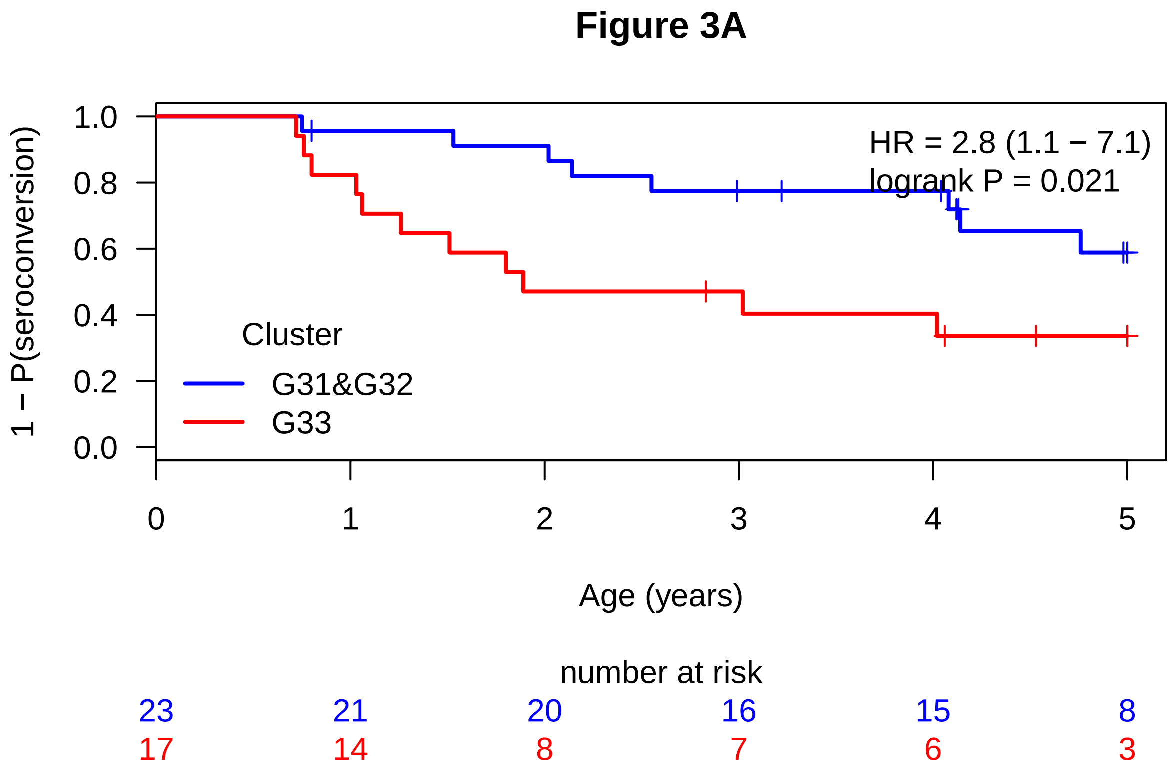

Here, a running average approach is used to plot Bacteroides and Akkermansia abundances in subgroups G31 and G32 vs subgroup G33 over time (Figure 3B and 3C).

```
tmp.C3 <- cluster.C3
tmp.C3[cluster.C3 == 3] <- 1
tmp.C3[cluster.C3 == 1] <- 3
tmp.C3[tmp.C3 == 2] <- 1
tmp.C3[tmp.C3 == 3] <- 2
ss <- seq(0.5, 2, 0.01)
clusters <- c(1, 2)
col <- c("blue", "red")
col.polygon <- c(rgb(0, 0, 1, alpha = 0.1)
                 , rgb(1, 0, 0, alpha = 0.1))

rownames(mat.genus.timeseries) <- metadata[ , "child_ID"]
genus <- c("Bacteroides", "Akkermansia")
fig <- c("Figure 3B", "Figure 3C")
names(fig) <- genus
maxima <- rep(NA, length(clusters))
list.data.clusters <- vector(mode = "list", length = 2)
for(z in genus){
  for(i in 1:length(clusters)){
    list.data.clusters[[i]] <- fun.get.data.window(data = mat.genus.timeseries, age = age
                                , clusters = tmp.C3, clust.num = clusters[i]
                                                           , window = 0.5, sequence = ss
                                                           , genus = z)
```

```
    maxima[i] <- max(list.data.clusters[[i]][1, ] + list.data.clusters[[i]][2, ])
  }

  for(i in 1:length(clusters)){
    par(las = 1)
    par(mar = c(5, 5, 4, 2))
    if(i == 1)
      plot(ss, list.data.clusters[[i]][1, ], type = "l"
           , ylab = "Mean Relative Abundance", xlab = "Age(years)"
           , col = col[i], ylim = c(0, max(maxima, na.rm = TRUE) + 0.05)
           , main = fig[z], lwd = 2)
    else
        points(ss, list.data.clusters[[i]][1, ], type = "l", col = col[i], lwd = 2)

    for(kk in 2:length(ss)){
    polygon(c(ss[kk-1], ss[kk-1], ss[kk], ss[kk])
            , c(list.data.clusters[[i]][1, kk-1] - list.data.clusters[[i]][2, kk-1]
                , list.data.clusters[[i]][1, kk-1] + list.data.clusters[[i]][2, kk-1]
                , list.data.clusters[[i]][1, kk-1] + list.data.clusters[[i]][2, kk]
                , list.data.clusters[[i]][1, kk-1] - list.data.clusters[[i]][2, kk])
            , col = col.polygon[i], border = NA)
    }
  }
  legend("topright", c("G31&G32", "G33"), col = c("blue", "red")
          , lty = 1, lwd = 2, bty = "n")
  legend("bottomright", z, bty = "n")
}
```

Figure 3B

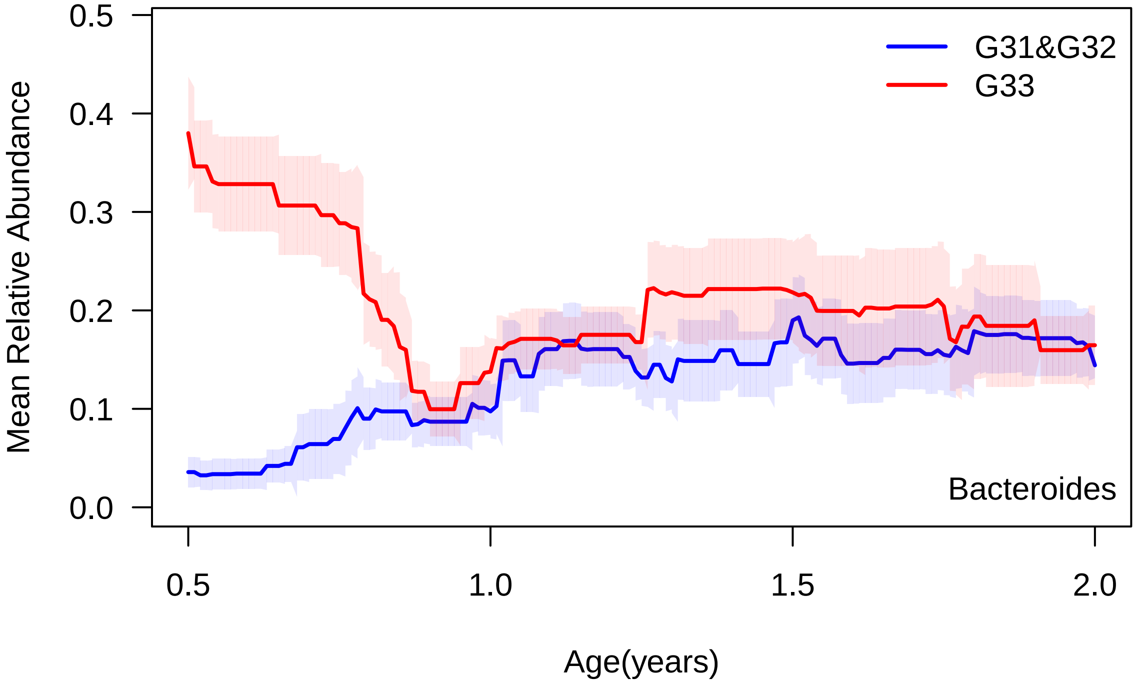
G31&G32
G33
Bacteroides
Mean Relative Abundance
0.0
0.1
0.2
0.3
0.4
0.5
0.5
1.0
1.5
2.0
Age(years)

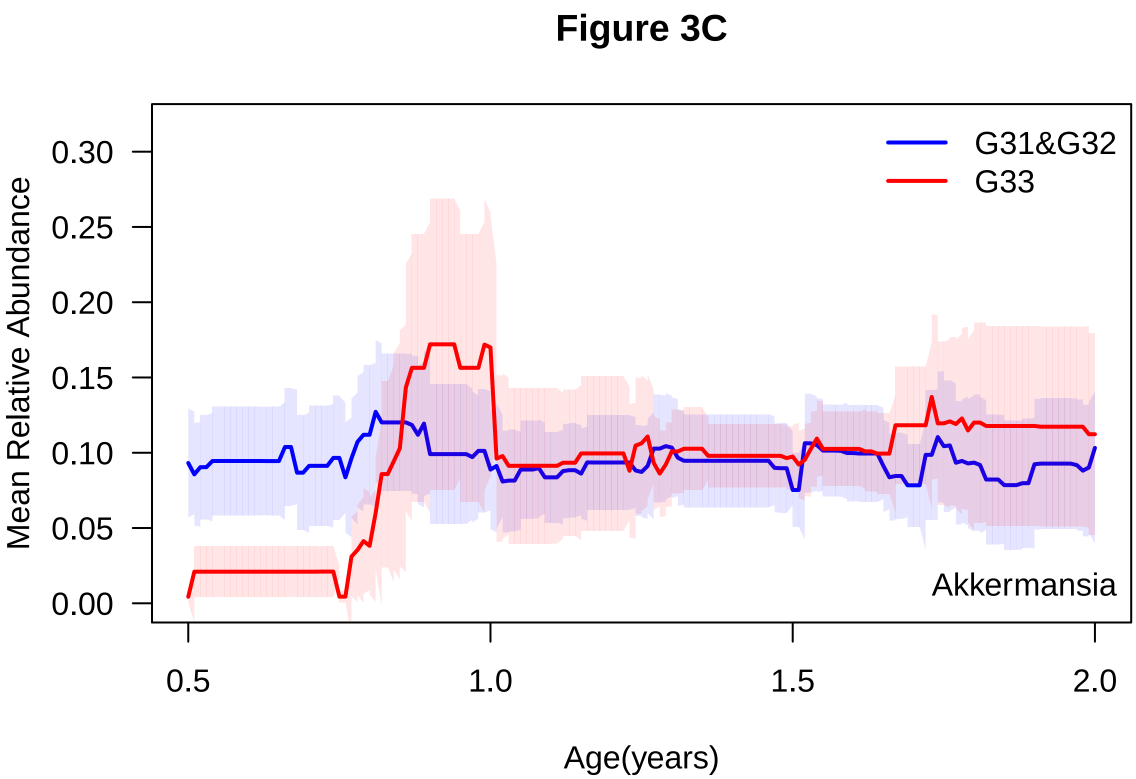

KO data estimated with the PICRUSt method is used to compare the genetic potential to produce butyrate for the last steps of the the but (K01034 and K01035) and buk (K00634 and K00929) pathways between subgroups G31 and G32 vs subgroup G33 (Figure 3D).

```
ko.data.early <- sweep(ko.data.early, MARGIN = 2, FUN = "/", colSums(ko.data.early))
ko.data.early <- ko.data.early[, names(tmp.C3)]
butyrate.proportion <- (ko.data.early["K01034", ] +
                          ko.data.early["K01035", ])/(ko.data.early["K00634", ] +
                                                          ko.data.early["K00929", ])

par(las = 1)
par(mar = c(5, 5, 4, 2))
p.butyrate <- wilcox.test(butyrate.proportion ~ cluster.C3 == 1)$p.value
boxplot(log(butyrate.proportion) ~ cluster.C3 == 1, names = c("G31&G32", "G33")
            , ylab = "log(Proportion): but vs buk", main = "Figure 3D")
beeswarm(log(butyrate.proportion) ~ cluster.C3 == 1, add = TRUE, pch = 16)
legend("topright", paste("P=", signif(p.butyrate, 1), sep = ""), bty = "n")
```

Figure 3D

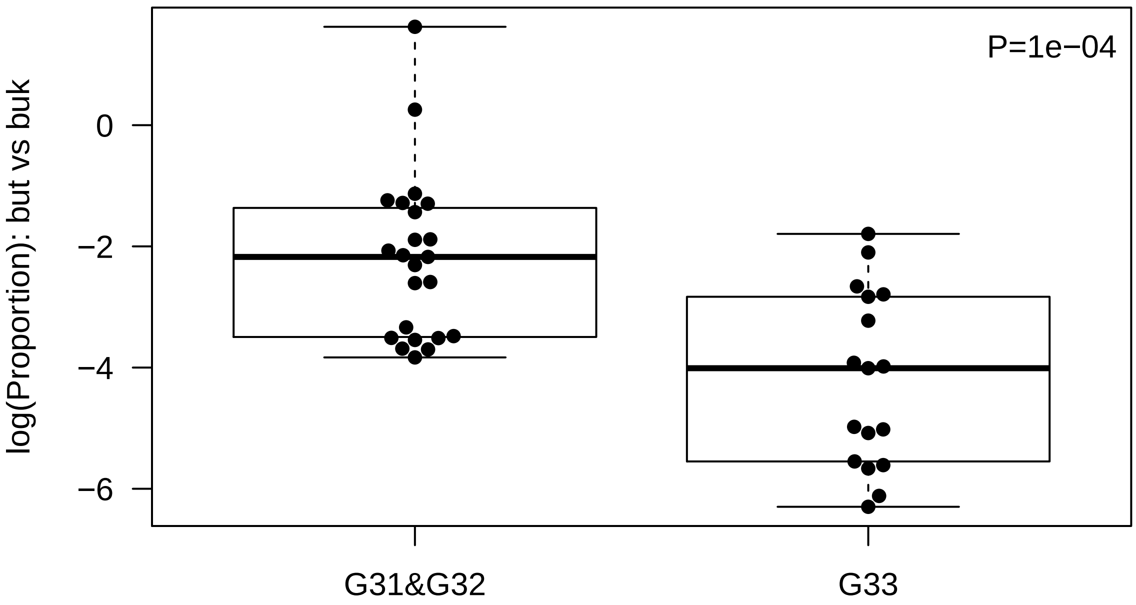

MATLAB Code

Required packages

Communities are estimated based on the MATLAB stability package by Schaub et al. The package has been downloaded and installed from

https://github.com/michaelschaub/PartitionStability.

Functions for the detection of the largest connected component have been obtained from the MATLAB package gaimc which has been downloaded from

http://www.mathworks.com/matlabcentral/fileexchange/24134-gaimc—graph-algorithms-in-matlab-code.

Estimation of communities based on Schaub et al.

In the first step, the adjacency matrix estimated based on the CCREPE method in R (see above) is loaded into MATLAB. Next, the largest connected component is calculated and the stability algorithm is applied to identify microbial communities. The number of communities is identified based on the resulting stability plot by choosing a number larger than two showing longest stability with respect to Markov time. The resulting vector of community memberships

is then stored in the file “network_combined_early_13_8.xls” which is then imported into R for all further analyses.

```
clear all;
close all;

[A1, TXT] = xlsread('H:/knitr/Data/adj.matrix.ccrepe.13_8.xls');
s= size(A1)

T = 10.^[-2:0.05:3];
[l_A1, p] = largest_component(A1);

h1 = figure;
[S, N, VI, C] = stability(l_A1, T,'plot','v')

index = 70
com = zeros(s(1), 1)
com(find(p)) = C(:, index)
com(find(¬p)) = -1

xlswrite('H:/knitr/Data/network_combined_early_13_8.xls', com)
```
