# Supplementary material for: Towards a functional hypothesis relating anti-islet cell autoimmunity to the dietary impact on microbial communities and butyrate production
Source: Microbiome. 2016 Apr 26;4:17. doi: 10.1186/s40168-016-0163-4 (PMC4845316; doi:10.1186/s40168-016-0163-4)
Supplement: Additional file 4: — Figures S1–S4. (PDF 1876 kb) [file 40168_2016_163_MOESM4_ESM.pdf]

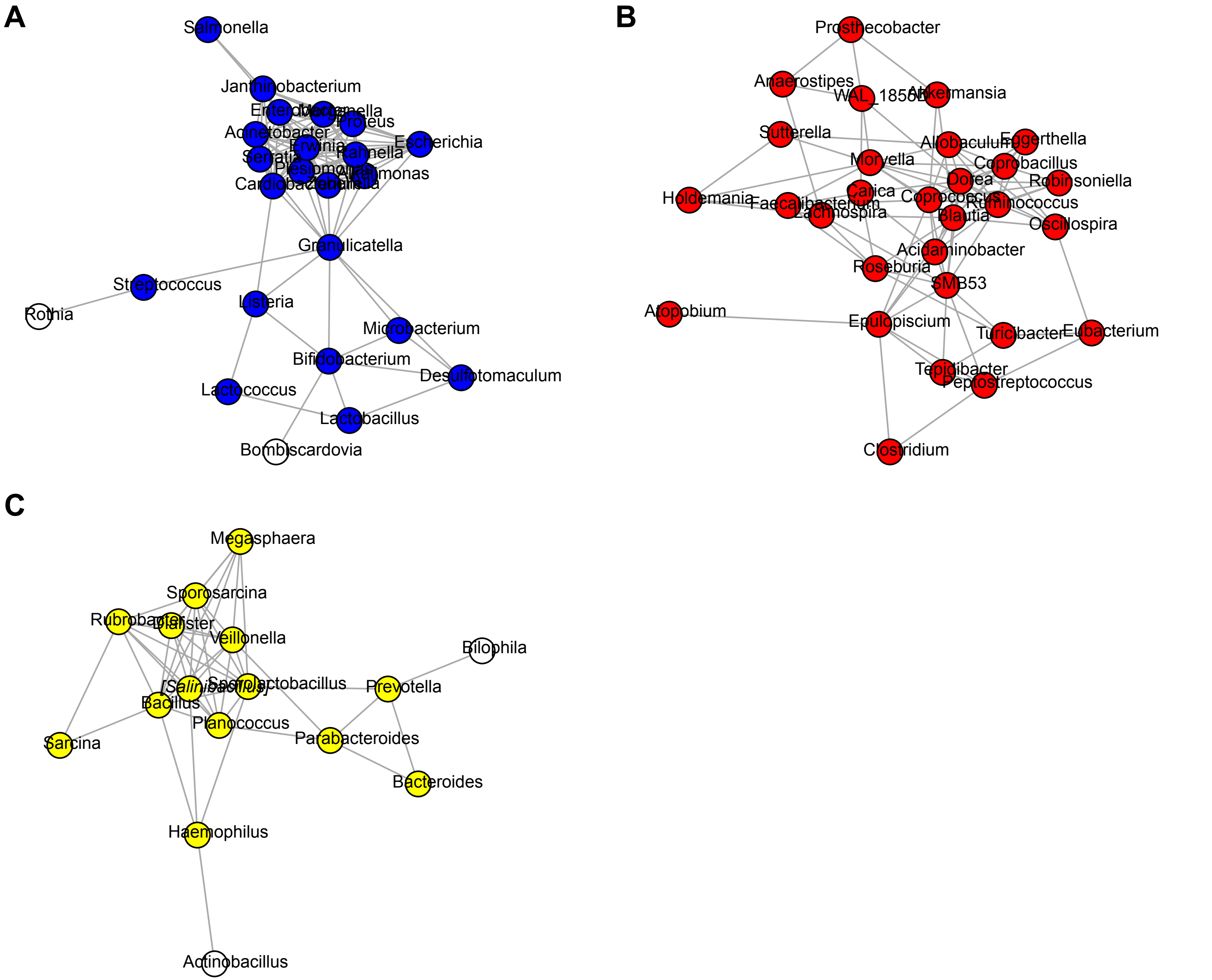


**Figure S1. Subnetworks of communities C1, C2 and C3.**
**(A)** Subnetwork for community C1 including names of the genera. **(B)** Subnetwork for community C2 including names of the genera. (**C**) Subnetwork for community C3 including names of the genera.

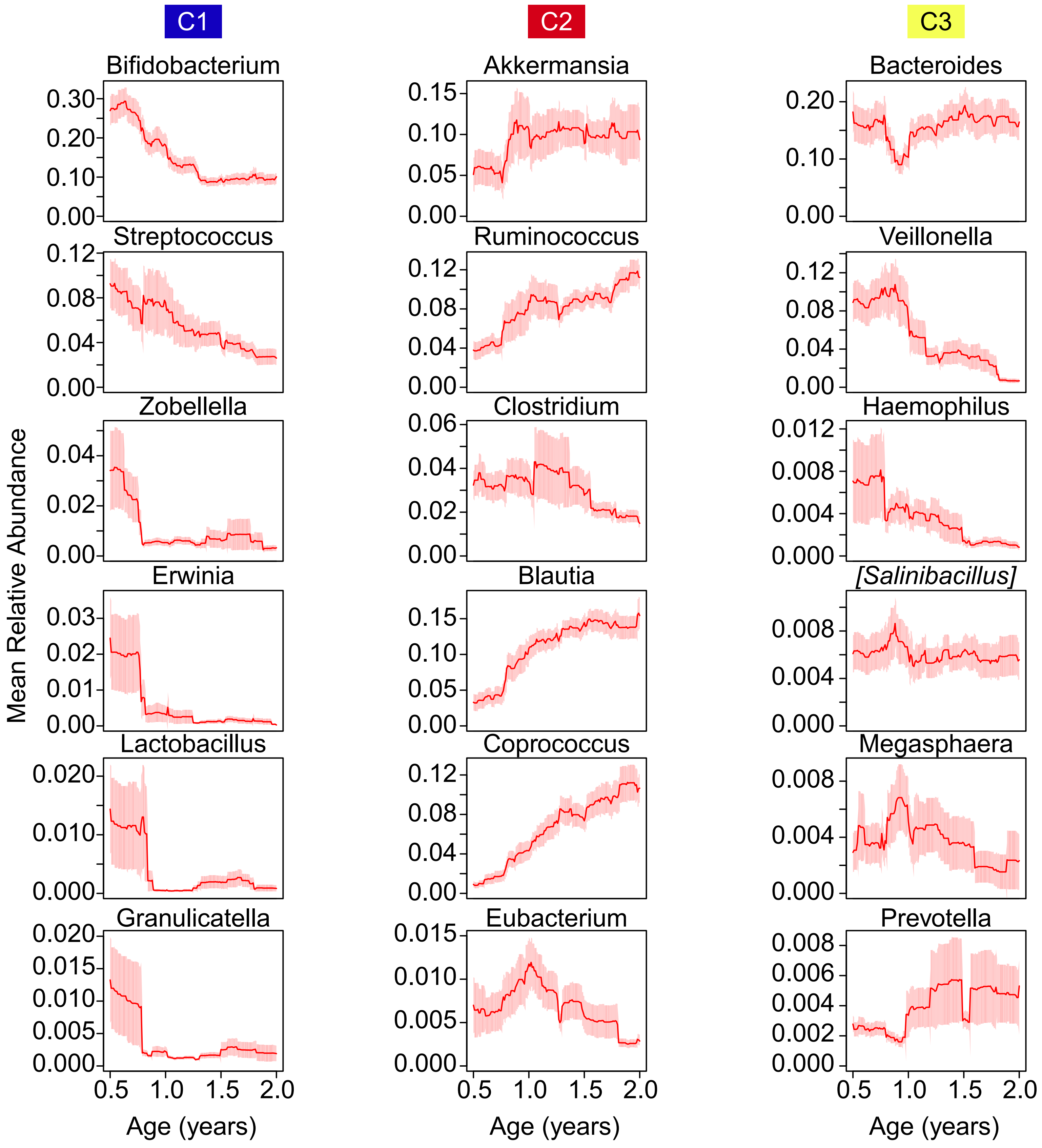


**Figure S2. Temporal development of bacteria in communities C1, C2 and C3.**
The figure shows the temporal development of the abundances of the six highest abundant genera. A moving window approach was used to estimate the mean (red line) and standard error of the mean (red band) in each window of size 0.5 years. The step size was chosen as 0.01 years.

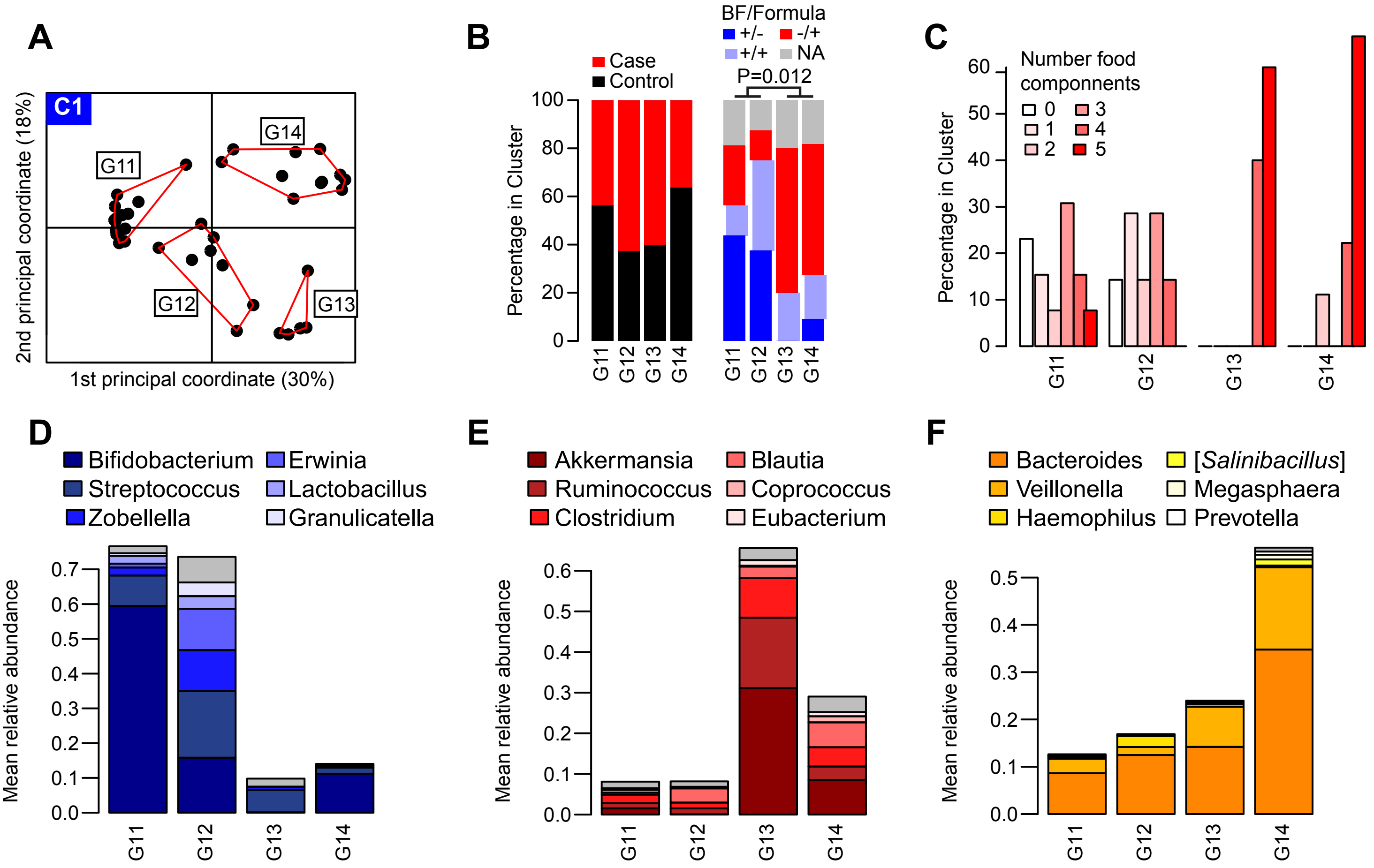


**Figure S3. Stratification of children based on genera in C1.**

**(A)** The PCoA plot shows four clusters (G11, G12, G13, G14) of children identified by PAM clustering of UniFrac distances from abundances of genera in C1. **(B)** Percentages of autoantibody positive and autoantibody negative, and breast fed vs formula fed infants in each subgroup. **(C)** Percentage of the number of solid food components (vegetables+potatos+fruits+meat+formula) in each subgroup.

**(D)** Mean relative abundance of the six dominant genera in community C1. **(E)** Mean relative abundance of the six dominant genera in community C2. **(F)** Mean relative abundance of the six dominant genera in community C3.

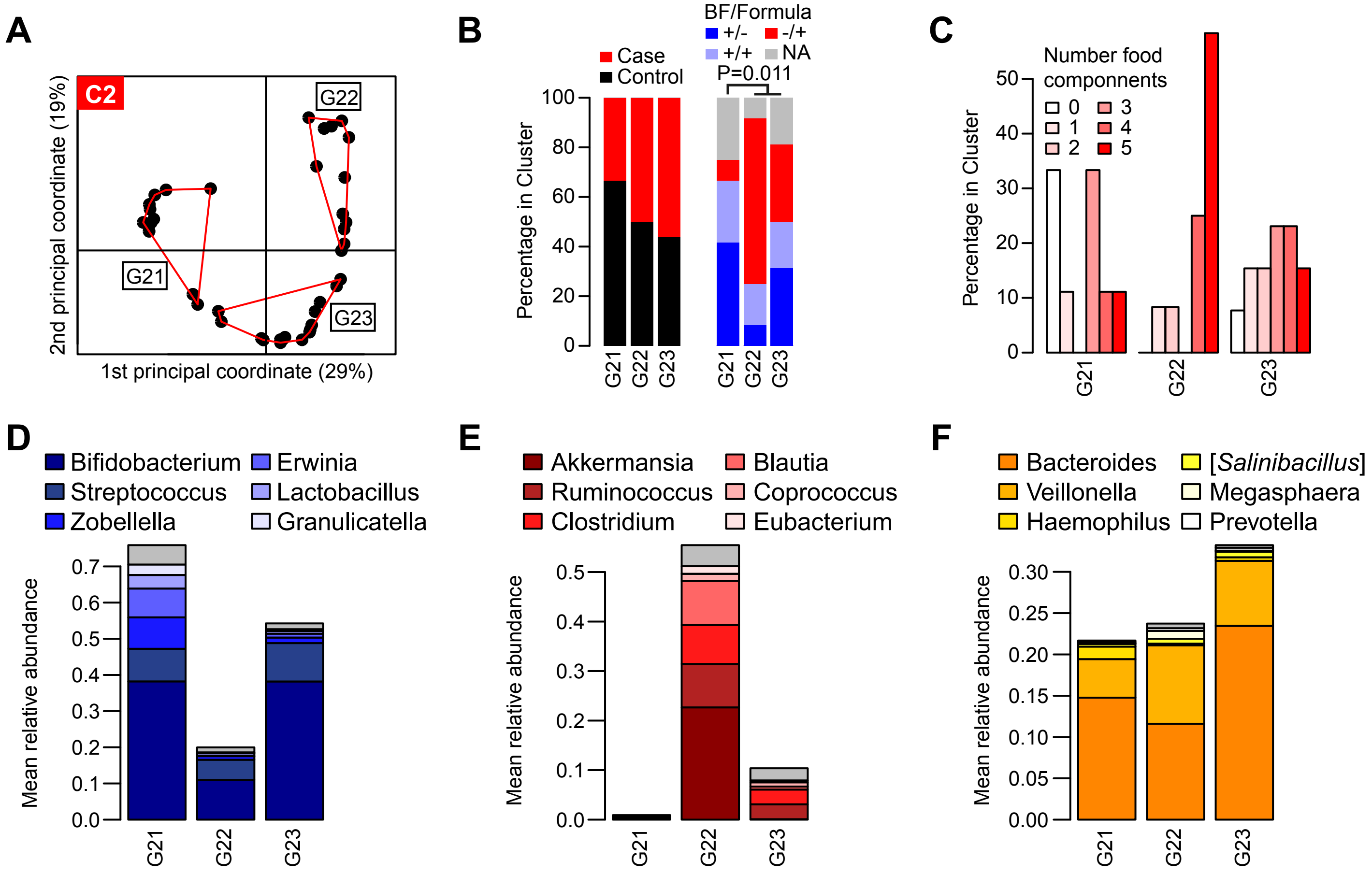


**Figure S4. Stratification of children based on genera in C2.**
**(A)** The PCoA plot shows three clusters (G21, G22, G23) of children identified by PAM clustering of UniFrac distances from abundances of genera in C2. **(B)** Percentages of autoantibody positive and autoantibody negative, and breast fed vs formula fed infants in each subgroup. **(C)** Percentage of the number of solid food components (vegetables+potatos+fruits+meat+formula) in each subgroup.
**(D)** Mean relative abundance of the six dominant genera in community C1. **(E)** Mean relative abundance of the six dominant genera in community C2. **(F)** Mean relative abundance of the six dominant genera in community C3.
