# Supplementary material for: Towards a functional hypothesis relating anti-islet cell autoimmunity to the dietary impact on microbial communities and butyrate production
Source: Microbiome. 2016 Apr 26;4:17. doi: 10.1186/s40168-016-0163-4 (PMC4845316; doi:10.1186/s40168-016-0163-4)
Supplement: Additional file 5: — Table S1, stratifications of children based on abundances in communities C1, C2 and C3. (PDF 27 kb) [file 40168_2016_163_MOESM5_ESM.pdf]

Supplementary Table 1

Child ID	Cluster C1	Cluster C2	Cluster C3	Age* (years)	Status	Seroconv. age (years)	Max age (years)	Breast feeding*	Formula**	Potato**	Vegetable**	Fruit**	Meat**
1	G11	G21	G31	0.55	Control	-	8.16	yes	no	no	no	no	no
2	G11	G21	G31	0.52	Control	-	3.22	yes	yes	yes	yes	no	no
3	G11	G21	G31	0.49	Control	-	0.80	-	-	no	yes	no	no
4	G11	G21	G32	0.4	Control	-	6.10	yes	no	no	no	no	no
5	G11	G21	G32	0.29	Control	-	5.55	no	yes	no	no	no	no
6	G11	G21	G33	0.52	Case	0.80	1.10	yes	-	no	yes	no	no
7	G11	G22	G32	0.55	Control	-	4.12	yes	no	yes	yes	no	no
8	G11	G23	G31	0.52	Control	-	6.12	yes	yes	yes	yes	no	no
9	G11	G23	G32	0.53	Case	1.53	5.73	yes	no	yes	yes	yes	yes
10	G11	G23	G32	0.53	Case	2.02	5.78	no	yes	yes	yes	yes	yes
11	G11	G23	G32	0.5	Case	2.55	6.02	yes	no	no	no	no	no
12	G11	G23	G32	0.52	Control	-	7.29	no	yes	no	yes	yes	no
13	G11	G23	G33	0.55	Case	3.02	8.17	no	yes	yes	yes	yes	no
14	G11	G23	G33	0.56	Case	1.80	5.67	yes	no	yes	yes	no	yes
15	G11	G23	G33	0.59	Case	1.89	4.05	-	-	yes	yes	yes	-
16	G11	G23	G33	0.52	Control	-	8.08	yes	no	no	yes	no	no
17	G12	G21	G31	0.52	Case	2.14	3.22	yes	yes	yes	yes	no	no
18	G12	G21	G32	0.5	Control	-	2.99	yes	no	no	no	no	no
19	G12	G21	G33	0.53	Case	1.26	5.99	yes	no	yes	yes	yes	yes
20	G12	G21	G33	0.59	Case	1.51	4.73	yes	no	yes	yes	no	yes
21	G12	G21	G33	0.56	Control	-	7.97	yes	-	yes	yes	yes	yes
22	G12	G22	G31	0.41	Case	4.08	8.45	no	yes	no	no	no	no
23	G12	G23	G31	0.52	Control	-	6.15	yes	yes	no	yes	no	no
24	G12	G23	G32	0.44	Case	4.76	7.62	yes	yes	no	no	no	no
25	G13	G22	G31	0.52	Case	0.75	0.75	-	no	yes	yes	yes	yes
26	G13	G22	G32	0.51	Case	4.14	5.23	yes	yes	yes	yes	yes	no
27	G13	G22	G32	0.43	Control	-	4.04	no	yes	yes	yes	yes	yes
28	G13	G22	G33	0.52	Case	1.06	6.53	no	yes	yes	yes	yes	yes
29	G13	G22	G33	0.53	Control	-	2.83	no	yes	yes	yes	yes	yes
30	G14	G21	G33	0.54	Control	-	7.13	yes	yes	yes	yes	yes	yes
31	G14	G22	G31	0.64	Control	-	8.09	no	yes	yes	yes	yes	yes
32	G14	G22	G31	0.52	Control	-	4.98	no	yes	yes	yes	yes	no
33	G14	G22	G31	0.62	Control	-	4.13	yes	yes	yes	yes	yes	yes
34	G14	G22	G33	0.59	Case	4.02	4.02	no	yes	yes	yes	yes	yes
35	G14	G22	G33	0.47	Case	0.72	6.46	no	yes	yes	yes	yes	yes
36	G14	G23	G31	0.63	Control	-	7.19	yes	no	yes	yes	yes	yes
37	G14	G23	G33	0.62	Case	0.76	0.99	-	no	yes	yes	no	no
38	G14	G23	G33	0.62	Case	1.03	5.28	no	yes	yes	yes	yes	-
39	G14	G23	G33	0.54	Control	-	4.53	-	-	yes	yes	no	yes
40	G14	G23	G33	0.65	Control	-	4.06	no	yes	yes	yes	yes	yes

(*) at sampling time
(**) introduced before sampling time
